# Persistent dopamine-dependent remodeling of the neural transcriptome in response to pregnancy and postpartum

**DOI:** 10.1101/2025.02.20.639313

**Published:** 2025-02-25

**Authors:** Jennifer C Chan, Giuseppina Di Salvo, Ashley M Cunningham, Sohini Dutta, Elizabeth A Brindley, Ethan Wan, Cindy Zhang, Ian Maze

**Affiliations:** 1 Nash Family Department of Neuroscience, Friedman Brain Institute, Icahn School of Medicine at Mount Sinai, New York, NY, 10029, USA; 2 Department of Psychiatry and Neuropsychology, School for Mental Health and Neuroscience (MHeNs), Maastricht University, Maastricht, The Netherlands; 3 Department of Pharmacological Sciences, Icahn School of Medicine at Mount Sinai, New York, NY, 10029, USA; 4 Department of Neurobiology, University of Alabama at Birmingham, Birmingham, AL 35294, USA.; 5 Howard Hughes Medical Institute, Icahn School of Medicine at Mount Sinai, New York, NY, 10029, USA

## Abstract

Pregnancy and postpartum experiences represent transformative physiological states that impose lasting demands on the maternal body and brain, resulting in lifelong neural adaptations. However, the precise molecular mechanisms driving these persistent alterations remain poorly understood. Here, we used brain-wide transcriptomic profiling to define the molecular landscape of parity-induced neural plasticity, identifying the dorsal hippocampus (dHpc) as a key site of transcriptional remodeling. Combining single-cell RNA sequencing with a maternal-pup separation paradigm, we additionally demonstrated that chronic postpartum stress significantly disrupts dHpc adaptations by altering dopamine dynamics, leading to dysregulated transcription, altered cellular plasticity, and impaired behavior. We further established the sufficiency of dopamine modulation in the regulation of these parity-induced adaptations via chemogenetic suppression of dopamine release into dHpc, which recapitulated key transcriptional and behavioral features of parity in virgin females. In sum, our findings establish dopamine as a central regulator of parity-induced neuroadaptations, revealing a fundamental transcriptional mechanism by which female reproductive experiences remodel the maternal brain to sustain long-term behavioral adaptations.

## INTRODUCTION

Matrescence – the physical, emotional, hormonal, and social transition to motherhood – is a period of profound transformation that reshapes the maternal body and brain to support pregnancy, birth, and offspring care. While extensive research has focused on acute maternal brain processes that are essential for the onset and maintenance of parenting, how precisely brain adaptations are sustained beyond the postpartum period to persistently influence behavior remains unclear. Recent human neuroimaging studies have revealed that pregnancy experience induces long-lasting alterations in brain connectivity and structure, persisting for years or even decades following birth^1–6^. Animal models examining parity – the state of previously carrying a pregnancy to term – similarly have demonstrated persistent alterations in synaptic remodeling, cell proliferation, and behavioral outcomes^7–14^. However, the specific mechanisms within the extensive repertoire of maternal physiological changes that drive these long-lasting brain adaptations remain poorly understood. With over 130 million pregnancies occurring globally each year, elucidating how female reproductive experiences shape long-term brain plasticity remains a fundamental question with widespread clinical implications. Given that parity may modulate risk and/or resilience to certain neurological and neuropsychiatric disorders^15–17^, addressing this gap in knowledge will be essential for advancing our understanding of maternal brain health.

Here, we began by employing brain-wide transcriptional profiling to systematically characterize the persistent impact of parity on the maternal brain in mice, which provided important insights into the regulation of signaling pathways and cellular compositions across maternal circuits. These analyses surprisingly revealed the dorsal hippocampus (dHpc) as a key site of sustained plasticity. We then leveraged single-cell RNA sequencing, a postpartum stress paradigm, behavioral assays, and chemogenetic approaches to identify and validate a dopamine-dependent neuromodulatory process that orchestrates these sustained maternal adaptations. Importantly, these brain-wide datasets will also serve as a critical resource for future investigations into the reproductive state-dependent adaptations that contribute to matrescence.

## RESULTS

### Parity promotes long-lasting transcriptional and behavioral adaptations in maternal brain

**Figure 1.**
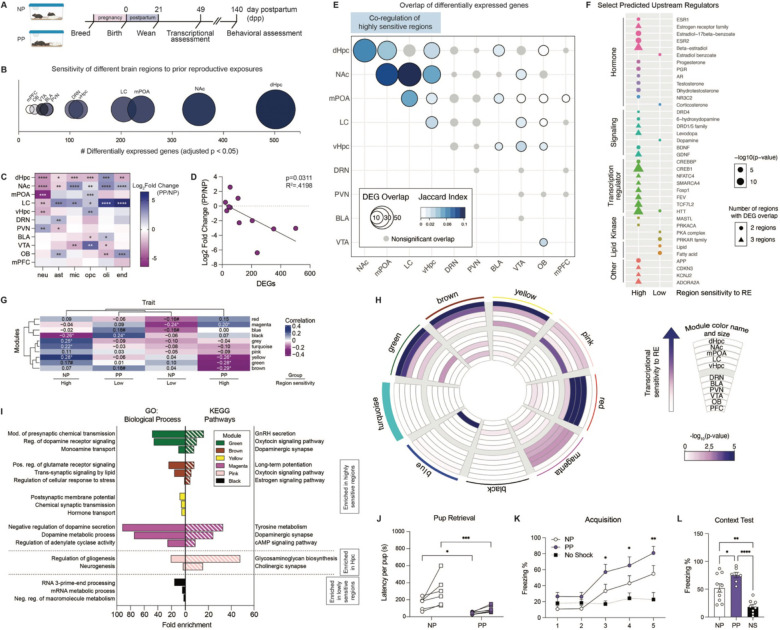
Pregnancy and postpartum states promote lasting transcriptional and behavioral adaptations in the maternal brain. **A)** Experimental timeline comparing primiparous (PP) *vs.* age-matched nulliparous (NP) female mice 1-month after offspring weaning (day postpartum, 49 dpp), and behavioral outcomes at 140 dpp. **B)** Differentially expressed genes (DEGs) per brain region. Bubble size represents total number of NP *vs.* PP DEGs (adj. p < 0.05). **C)** Cell-type deconvolution of bulk RNA-seq data (NP *vs.* PP normalized surrogate proportion variables, Student’s t-test with multiple comparisons correction; *FDR < 0.05, **FDR < 0.01, ***FDR < 0.001, ****FDR < 0.0001). **D)** Downregulation of neuron proportion significantly correlated with number of DEGs per brain region (R^2^ = 0.4198, p = 0.0311). **E)** Overlap of DEGs across brain regions (p < 0.05; gray indicates nonsignificant overlap). **F)** Select predicted upstream regulators of overlapping DEGs across regions with high (dHpc, NAc, mPOA, LC, vHpc) *vs.* low (DRN, PVN, BLA, VTA, OB, mPFC) sensitivity to parity. **G)** Trait heatmap correlating gene co-expression modules (arbitrary color names, identified by weighted gene correlation network analysis) with group × brain region classification (*p < 0.05, #p < 0.1). **H)** Circos plot for gene co-expression modules, with the size and arbitrary color name of each module indicated by the arc thickness along the perimeter. Enrichment for DEGs per brain region for each module is indicated by the inner rings, with color indicating significance of overlap. **I)** Select GO Biological Processes and KEGG Pathways significantly enriched from gene co-expression modules (FDR < 0.05). **J)** PP dams retrieved both pups significantly faster compared to NP females at 140 dpp (two-way rmANOVA, main effect of group (F(1,11) = 14.47, p = 0.0029), main effect of pup number (F(1,11) = 11.36, p = 0.0062)), with significantly reduced latency for retrieval of pup 1 (Holm-Šídák’s multiple comparisons test, t(22) = 2.301, *adj. p = 0.0312) and pup 2 (Holm-Šídák’s multiple comparisons test, t(22) = 4.098, ***adj. p = 0.0009). N = 6–7 animals/group. **K)** PP dams exhibited increased freezing behavior during the acquisition phase of contextual fear conditioning (two-way rmANOVA, main effect of group (F(2,21) = 4.259, p = 0.028), main effect of preshock (F(2.827, 59.36) = 26.59, p < 0.0001), interaction (F(8,84) = 5.091, p < 0.0001)), compared to no shock controls (Dunnett’s multiple comparisons test, *p £ 0.05, **p < 0.01). **L)** During the context recall test, PP dams froze significantly more compared to NP (one-way ANOVA, F(2,22) = 18.72, p < 0.0001; Tukey’s multiple comparisons test, t(22) = 3.640, *p = 0.044), with both groups demonstrating contextual learning compared to no shock controls (PP vs. NS, t(22) = 8.541, ****p < 0.0001; NP vs. NS, t(22) = 5.538, **p = 0.0021). N = 7–10 animals/group. Error bars represent mean ± SEM.

To first investigate the persistent effects of female reproductive experiences in maternal brain, we established a timeline to compare primiparous dams (PP), which experienced breeding, pregnancy, parturition, lactation, and pup interactions, to age-matched virgins that lacked reproductive experiences (nulliparous, NP; Fig. 1A). Given that reproductive hormones return to baseline levels ~7-weeks post-conception^18^, we then conducted brain-wide transcriptomic profiling 4-weeks following cessation of pup rearing (10-weeks post-conception; i.e. 49 days postpartum; dpp), as this time point would be predicted to reflect sustained changes. Following differential expression analyses of the 11 brain regions selected based on prior evidence supporting their involvement in maternal behaviors^19,20^ (Fig. S1A), our data revealed a wide-range in the number of differentially expressed genes (DEGs, adj. p < 0.05) across brain regions (Fig. 1B, Table S1). These differences indicated varying levels of transcriptomic sensitivity to parity brain-wide, with few changes observed in the medial prefrontal cortex (mPFC), for example, as compared to the dHpc, the region that was found to be most robustly regulated in our analyses.

To next assess whether these differential expression patterns could be attributed to changes in shared cell-types, we performed cell-type deconvolution using BRETIGEA, which compares whole transcriptome data to validated brain cell type-specific marker gene sets^21^. We observed changes in cell marker expression for neurons, astrocytes, microglia, oligodendrocyte precursor cells (OPCs), oligodendrocytes, and endothelial cells across brain regions, regardless of the number of DEGs (except for mPFC; Fig. 1C). These data suggested that cellular changes may occur across brain regions independently of the extent of transcriptomic alterations, potentially reflecting shifts in the proportion of neurons to non-neuronal/glial cells, modifications in synaptic plasticity, or changes in cell identity in response to prior reproductive experiences. Notably, the expression of neuronal markers was found to be downregulated in brain regions displaying the most DEGs (Fig. 1C). Furthermore, a significant inverse correlation between reduced neuronal marker expression and the number of DEGs across all brain regions implicated changes in neuronal populations that may underlie the observed transcriptional responses (Fig. 1D); note that this relationship was not observed for other cell-types (Fig. S1B–F). These findings nicely align with human neuroimaging studies, which have reported reduced gray matter in mothers even years after birth, a phenomenon that has been proposed to refine maternal neural circuits to optimize caregiving behavior^1,2,4,22^.

Based on these findings, we hypothesized that such shared neuronal marker alterations may indicate common upstream mechanisms driving regional-specific transcriptional responses. To test this, we first compared DEGs across brain regions to identify common gene sets that were altered by parity status, which led to the observation that the greatest extent of overlap occurs in those regions displaying the highest levels of transcriptional sensitivity (Fig. 1E). Based on these analyses, we then grouped the 11 brain regions according to the number of DEGs, the degree of overlap with other regions, and significant fold changes in neuronal marker expression, based on the premise that these regions may be co-regulated to elicit convergent transcriptional responses to parity (referred to as “high sensitivity” regions: dHpc, nucleus accumbens (NAc), medial preoptic area (mPOA), locus coeruleus (LC), ventral hippocampus (vHpc)). Brain regions lacking these criteria were classified as “low sensitivity” regions (dorsal raphe nucleus (DRN), paraventricular nucleus of the hypothalamus (PVN), basolateral amygdala (BLA), ventral tegmental area (VTA), olfactory bulb (OB), mPFC). Next, we explored the predicted upstream regulators of overlapping DEGs in “High” versus “Low” sensitivity brain regions, which revealed several upstream regulators that were grouped into common molecular classes, including hormones, neurotransmitter/neurotrophin signaling molecules, transcriptional regulators, and others (Fig. 1F). While both High and Low sensitivity regions were found to share upstream regulators within these molecular classes, High sensitivity regions displayed greater enrichment for estrogen, progesterone, testosterone, dopamine, and other ligands with similar receptor affinity or structural homology. In contrast, regulators that were classified within the lipid category were uniquely associated with Low sensitivity regions.

To further explore the regulatory networks that distinguish High versus Low sensitivity regions, we performed weighted gene co-expression network analysis (WGCNA) across all 11 brain regions^23^, resulting in nine co-expression modules (Fig. S1G–J). Module-trait correlations, in which the trait refers to a combination of group (NP *vs.* PP) and regional sensitivity (High *vs.* Low), identified significant correlations between High sensitivity regions in PP females and the brown, green, yellow, and magenta modules (Fig. 1G; henceforth labeled ‘parity-sensitive modules’). To identify the specific brain regions exhibiting changes in module gene expression, we then examined enrichment of DEGs within each module, with an extended focus on the parity-sensitive modules. Consistent with our module-trait correlational analysis, parity-sensitive modules displayed significant enrichment for DEGs from dHpc (Fig. 1H; Fisher’s exact tests: brown, p < 10^−16^; green, yellow, p < 10^−6^), NAc (brown, green, p < 10^−3^; magenta, p < 0.01; yellow, p < 0.05), mPOA (green, p < 0.05; magenta, p < 0.01), LC (magenta, p < 0.05), and vHpc (magenta, p < 0.05; yellow, p < 0.01). Functional annotation analysis of the genes from these modules highlighted their significant enrichment in signaling pathways related to neuromodulators, such as estrogen (yellow, brown) and dopamine (green, magenta), in agreement with our upstream regulator analyses (Fig. 1I, Fig. S1K–L). In contrast, Low sensitivity regions were found to be significantly associated with the black module, which was enriched for genes associated with RNA and macromolecule processing pathways (Fig. 1G, 1I).

**Figure 2.**
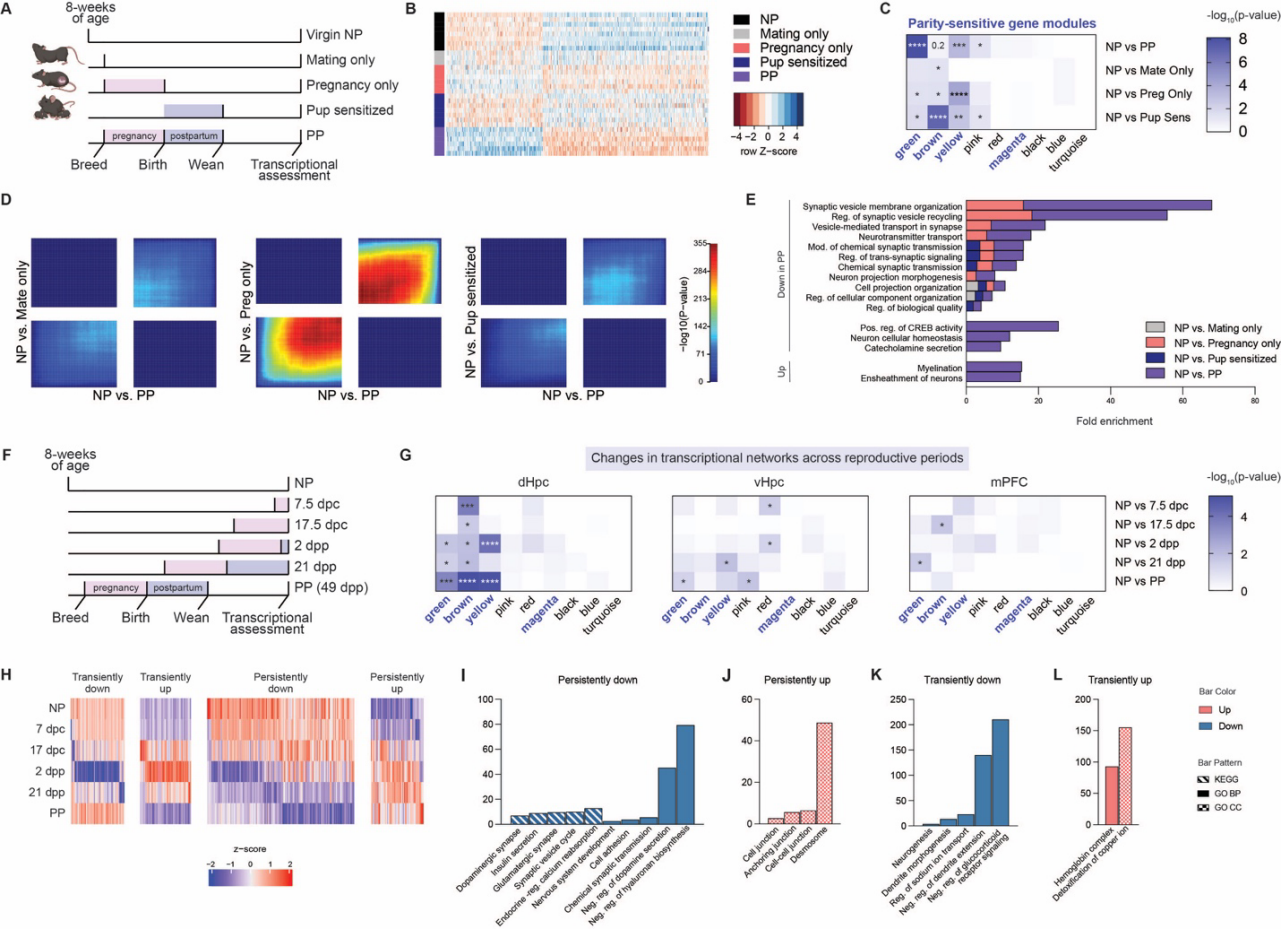
Parity adaptations in the dHpc transcriptome integrate pregnancy and postpartum experiences. **A)** Experimental design to examine the contribution of discrete female reproductive events. **B)** Heatmap of differential expression profiles of top 500 genes, by ascending p-values, between NP *vs.* PP (N=3–7/group). **C)** Overlap of DEGs (*vs.* NP, adj. p < 0.05) for each gene co-expression module, with color representing extent of significance (*p < 0.05, **p < 0.01, ***p < 0.001, ****p < 0.0001). High parity sensitivity modules are indicated in bold. **D)** Threshold-free comparison of indicated comparisons by rank-rank hypergeometric overlap. Pixels represent the overlap of differential expression profiles indicated, with color representing extent of significance. The lower left and upper right quadrants represent concordant gene regulation. **E)** Shared changes in biological processes (identified through GO term analysis) observed following individual reproductive exposures with PP, along with processes exclusive to the combined experiences in PP (FDR < 0.05). **F)** Experimental design to examine the trajectory of PP dHpc gene expression changes across pregnancy and postpartum periods (dpc: days post-conception; dpp: days postpartum). **G)** Enrichment of DEGs (*vs.* NP, adj. p < 0.05) with parity-sensitive gene co-expression modules across reproductive time points, examining extent of significant overlap in brain regions exhibiting high (left, dHpc), moderate (middle, vHpc), and low (right, mPFC) transcriptional sensitivity to parity status (*p < 0.05, **p < 0.01, ***p < 0.001, ****p < 0.0001). N=6/group. **H)** Impulse time course analysis of dHpc gene expression identified patterns of transient *vs.* persistent up- and downregulation across pregnancy and postpartum (FDR < 0.05). **I-L)** Select GO terms and KEGG pathways significantly enriched for dHpc genes exhibiting patterns of **(I)** persistent downregulation, **(J)** persistent upregulation, **(K)** transient downregulation, and **(L)** transient upregulation across reproductive stages (FDR < 0.05).

Building on these transcriptomic findings, we next investigated whether parity leads to sustained functional alterations in PP females. To evaluate the extent of such persistence, we performed behavioral assessments at 140 dpp, ~16-weeks after pup weaning (Fig. 1A). Behavioral tasks were selected to specifically assess functions associated with the brain regions displaying robust transcriptional changes, including maternal behavior, learning, and memory. To assess maternal behavior, we tested pup retrieval in the home cage by placing two donor pups in opposite corners away from the nest. PP dams were observed to retrieve pups with significantly reduced latency as compared to NP females (Fig. 1J), which is consistent with previous findings, though at a more extended timepoint in our studies^24^. Next, to investigate the functional impact of dHpc transcriptional changes, we utilized a contextual fear conditioning paradigm. Female mice were habituated to the training context before receiving five shocks (2-seconds each, 0.7 mA) with 90-second intertrial intervals. Both NP and PP females demonstrated significant training acquisition, as indicated by increased freezing compared to no-shock controls (Fig. 1K). Notably, PP females exhibited enhanced acquisition, with significantly increased freezing observed by the third shock, while NP females only displayed significant freezing at the fifth shock (Fig. 1K). During the context test conducted 24-hours later, both groups demonstrated recall, with PP females showing higher freezing responses than NP animals (Fig. 2L). Given the involvement of these brain regions in anxiety- and depressive-like behaviors, we also assessed affective phenotypes using the open field, light-dark box, and forced swim tests, however, no significant differences were observed (Fig. S2A–H). These results suggested that the transcriptional changes observed in parity-sensitive brain regions may promote maternal and dHpc-related behavioral outcomes, as has been reported previously^7,10,12,14,25–31^, though the mechanisms underlying such prolonged functional adaptations remained unclear. To account for the potential influence of estrous cycling on behavioral changes, vaginal swabs were collected immediately following testing, and behavioral analyses were stratified by estrous stage. No significant effects of estrous stage were observed (Fig. S2I–N), thereby supporting parity status as the primary driver of the observed behavioral differences.

### Pregnancy and postpartum experiences promote persistent maternal neuroplasticity

Since our paradigm comparing NP *vs.* PP females could not distinguish between the discrete reproductive event(s) that may be responsible for the sustained transcriptional alterations observed, we next sought to isolate those key events throughout the female reproductive window. To do so, we compared females that were successfully bred to males but did not become pregnant (Mating Only), females that experienced pregnancy and parturition but did not transition to postpartum due to pup removal on the day of birth (Pregnancy Only), and virgin females exposed to 21-days of donor pup interactions (Pup Sensitized). These groups were analyzed in parallel with age-matched NP and PP females to delineate the contributions of specific reproductive and maternal experiences (Fig. 2A). Consistent with previous studies^32^, four days of pup exposures sensitized virgin females to maternal behavior, as evidenced by increased crouching over pups and decreased pup avoidance by day 4 (Fig. S3A). To determine the processes conferring sustained transcriptional sensitivity, we focused our investigations on the dHpc due to its pronounced gene expression response to parity. When comparing NP *vs.* PP gene expression profiles, we observed that the Pregnancy Only group most closely resembled that of the expression profile of PP females, suggesting that pregnancy is a primary driver of dHpc neuroplastic changes (Fig. 2B, Fig. S3B). However, the magnitude of fold changes did not match that of the PP group, indicating that additional experiences, likely during postpartum, are also necessary to fully establish parity-induced changes in the dHpc.

While visualization of differential expression profiles in the Mating Only and Pup Sensitized groups did not mimic PP changes, comparisons to NP did identify significant DEGs across all groups, with the most substantial changes observed in the Pup Sensitized group, followed by the Pregnancy Only, and Mating Only groups (Fig. S3C–E, Table S2). Overlapping DEGs induced by discrete reproductive events significantly intersected with DEGs in PP animals, again suggesting that while pregnancy is the primary driver of parity-induced effects, each reproductive event contributes, in part, to this process (Fig. S3F). Next, we overlapped DEGs from each comparison with genes from parity-sensitive modules (from Fig. 1) to assess the contribution of each reproductive event to parity programming. Significant overlap was observed across all groups, with Pregnancy Only and Pup Sensitized groups displaying the strongest enrichment, indicating that these experiences play a key role in shaping persistent dHpc alterations (Fig. 2C). Thus, despite the lack of similarity between the overall expression profiles between Pup Sensitized *vs.* PP, gene expression changes induced by pup interactions likely converge on similar regulatory networks, albeit through different DEGs, which are critical for parity-driven transcriptional alterations. To further explore these relationships, we performed threshold-free Rank-Rank Hypergeometric Overlap (RRHO) analyses^33^, which demonstrated transcriptional concordance across experiences, with the strongest overlap observed in pregnancy (Fig. 2D). These findings suggest that reproductive exposures converge, or act additively, to drive the full extent of brain transcriptional changes. Furthermore, comparing GO term analyses of DEGs for each group revealed significant enrichment in overlapping biological processes, with many related to synaptic signaling and plasticity pathways (Fig. 2E). Notably, certain pathways were unique to the combination of reproductive exposures, including changes in CREB activity, catecholamine secretion, and myelination, indicating that these regulatory processes depend on multiple reproductive events to achieve full induction (Fig. 2E). Although this study did not address the specific contributions of parturition or lactation, our findings demonstrate that pregnancy establishes the foundation for parity-induced transcriptional alterations in dHpc, with additional postpartum exposures also required to fully promote these responses.

Given that postpartum experiences enhanced changes that were initiated during pregnancy, we conducted a controlled time course study to examine these transcriptional dynamics across gestational and postpartum periods in age-matched females (Fig. 2F). Pregnancy timepoints were selected to represent the anabolic and catabolic phases of gestation, corresponding to early (7.5 days post-conception, dpc) and late (17.5 dpc) stages, respectively. Postpartum timepoints were chosen to capture the immediate effects of hormonal withdrawal following parturition (2 dpp), as well as a later stage prior to pup weaning (21 dpp). Based on the hypothesis that parity-induced transcriptional alterations are progressively enhanced across reproductive stages, we first compared DEGs at each timepoint (*vs.* NP) to genes from parity-sensitive modules identified previously. DEGs from all comparisons significantly overlapped with the brown module (Fig. 2G, left; Table S3), suggesting that pathways enriched in this module are central to dHpc programming. Enrichment in the green and yellow modules was predominantly found to be induced by postpartum exposures, potentially amplifying the effects of the pathways driven by the brown module (Fig. 2G, left). To assess the regional specificity of these dynamic changes, we analyzed bulk RNA-seq data from the vHpc, a region that displayed moderate sensitivity to parity status (Fig. 2G, middle; Table S4), along with the mPFC, which exhibited few transcriptional alterations (Fig. 2G, right; Table S5). These regions exhibited limited overlap of DEGs with parity-sensitive module genes, further emphasizing the specificity of the pathways that drive long-term adaptations in brain regions displaying heightened sensitivity to parity.

To then characterize cumulative transcriptional changes that occur across reproductive stages, we performed a time course analysis using the ImpulseDE2 package to detect genes displaying sustained or transient expression alterations^34^. This analysis revealed a prominent set of genes that displayed persistent and progressive downregulation, with more pronounced alterations during postpartum (Fig. 2H). These genes were significantly enriched for processes related to glutamatergic synapses, dopaminergic signaling, and endocrine-related pathways (Fig. 2I). Conversely, genes with persistent upregulation were found to be enriched for pathways associated with cell junction dynamics (Fig. 2J), which is consistent with overall prolonged changes in synaptic plasticity and transmission. Additionally, we identified gene sets displaying transient alterations, including downregulation of neurogenesis-related pathways, and upregulation of copper ion metabolism (Fig. 2K–L); these data are consistent with other studies reviewed here^35,36^. In total, these findings highlight postpartum as a key window for reinforcing parity-induced alterations.

### Chronic maternal stress during postpartum disrupts dHpc adaptations

**Figure 3.**
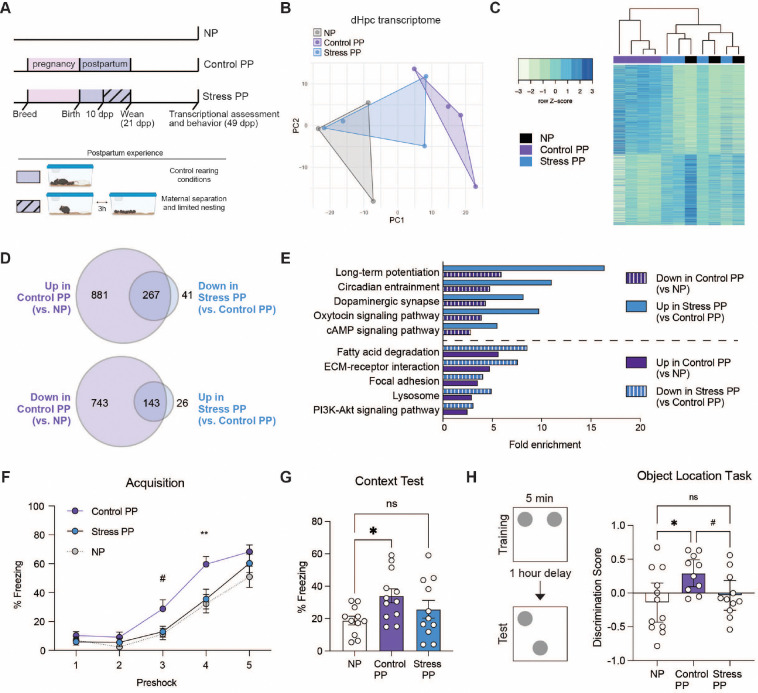
Postpartum stress disrupts long-term maternal dHpc adaptations. **A)** Postpartum stress paradigm, wherein dams were provided with limited nesting and subjected to pup separation 3 hours daily between 10–20 dpp, followed by behavioral and transcriptomic assessments at 49 dpp. **B)** Principal components analysis of dHpc transcriptomes, and **C)** heatmap of all significant DEGs (adj. p < 0.05) with hierarchical clustering. **D)** Venn Diagrams showing ~20% DEGs altered in Control PP (*vs.* NP) are significantly disrupted by postpartum stress (adj. p < 0.05). **E)** Parity-dependent enrichment in select KEGG pathways are significantly reversed in Stress PP (FDR < 0.05). **F)** Control PP dams froze more during the acquisition phase of contextual fear conditioning (two-way rmANOVA, main effect of group (F(2,31) = 4.662, p = 0.017), main effect of preshock (F(3.024,93.75) = 129.8, p < 0.0001), interaction (F(8,124) = 2.406, p = 0.091)), compared to NP and Stress PP (Tukey’s multiple comparisons test, #p ≤ 0.1, *p < 0.05, **p < 0.01). **G)** During the context recall test, NP froze significantly less than Control PP (one-way ANOVA, F(2,31) = 3.151, p = 0.05; Tukey’s multiple comparisons test, t(31) = 3.54, *p = 0.0456), but not Stress PP (ns, p > 0.05). N = 11–12 animals/group. **H)** Control PP, but not Stress PP, dams spent more time investigating the moved object in the object location task compared to NP (one way ANOVA, F(2,30) = 4.045, p = 2.0278; Holm-Šídák’s multiple comparisons test: NP vs. Control PP, t(30) = 2.764, *adj. p = 0.0288; Control PP vs. Stress PP, t(30) = 2.046, #adj. p < 0.1) N=10–12 animals/group. Error bars represent mean ± SEM.

We next examined whether postpartum perturbations may disrupt pathways that reinforce parity-induced alterations in dHpc. As becoming a new parent represents a significant source of stress, we implemented a maternal stress paradigm that robustly increases stress hormone levels and disrupts key postpartum experiences, including nursing and pup interactions, from 10–20 dpp (Fig. 3A, Fig. S3G). During this period, dams were separated from their pups for 3-hours daily and were provided with limited nesting materials until pup weaning (Stress PP). As litter size represents a potential confounding factor, the time period selected reduces the impact of maternal stress on pup mortality that can occur immediately following birth. By 10 dpp, pups were more durable, as reflected by the absence of litter size differences between Control and Stress dams (Fig. S3H). Accordingly, no differences in postpartum weights were observed, suggesting that this postpartum stress paradigm does not induce major metabolic alterations in the dams (Fig. S3I). To assess the extent of parity adaptations in Stress PP, we compared this group to NP and Control PP animals. Principal components analysis (PCA) of dHpc transcriptional profiles revealed distinct clustering of NP and Control PP groups (reproducing our prior analyses), while Stress PP samples were found to cluster intermediately between the two groups (Fig. 3B). Similarly, hierarchical clustering of all DEGs resulted in intermingling of NP and Stress PP samples (Fig. 3C, Table S6), suggesting that postpartum stress disrupts the extent of parity alterations in dHpc. To determine whether genes induced by parity are reversed by stress, we then compared DEGs with opposing directionality in NP *vs.* Control PP and Control PP *vs.* Stress PP groups (Fig. 3D). Approximately 85% of genes altered by maternal stress overlapped with parity-induced gene expression changes. KEGG pathway analysis of DEGs revealed shared enrichment across several pathways in both comparisons, with maternal stress reversing gene expression associated with long-term potentiation, dopaminergic synapses, oxytocin signaling, and other processes (Fig. 3E).

Since our pathway analyses indicated changes in long-term potentiation, a form of neuronal plasticity that is important for learning and memory behaviors^37^, we further focused our behavioral assessments on contextual fear conditioning. Consistent with prior results, Control PP females exhibited enhanced acquisition and context recall, as demonstrated by significantly increased freezing behavior compared to NP animals. In contrast, however, Stress PP females showed no significant differences *vs.* NP mice (Fig. 3F–G). To further evaluate dHpc-dependent function, we next assessed behavior using the object location task. In this test, animals were initially trained by allowing them to explore two identical objects, followed by one-hour of removal from the arena. During the test phase, one object was displaced, and the animals’ ability to identify the moved object was assessed. Given prior findings that adult females require 10 minutes of training for reliable discrimination^38^, we first used this duration to confirm that all groups could successfully identify the moved object (Fig. S3J). To next assess whether Control PP females exhibit enhancements in this task, we reduced the training time to 5 minutes, a duration that is sufficient for learning in adult males and preadolescent female mice, but not in adult females^38^. Under these conditions, Control PP females retained the ability to discriminate the novel location, whereas NP and Stress PP females did not, reflecting the same pattern observed in contextual fear conditioning, which was not attributed to estrous stage or locomotion (Fig. S3K–L).

### Cell-type specific transcriptional alterations reveal dopaminergic modulation underlying maternal dHpc plasticity

**Figure 4.**
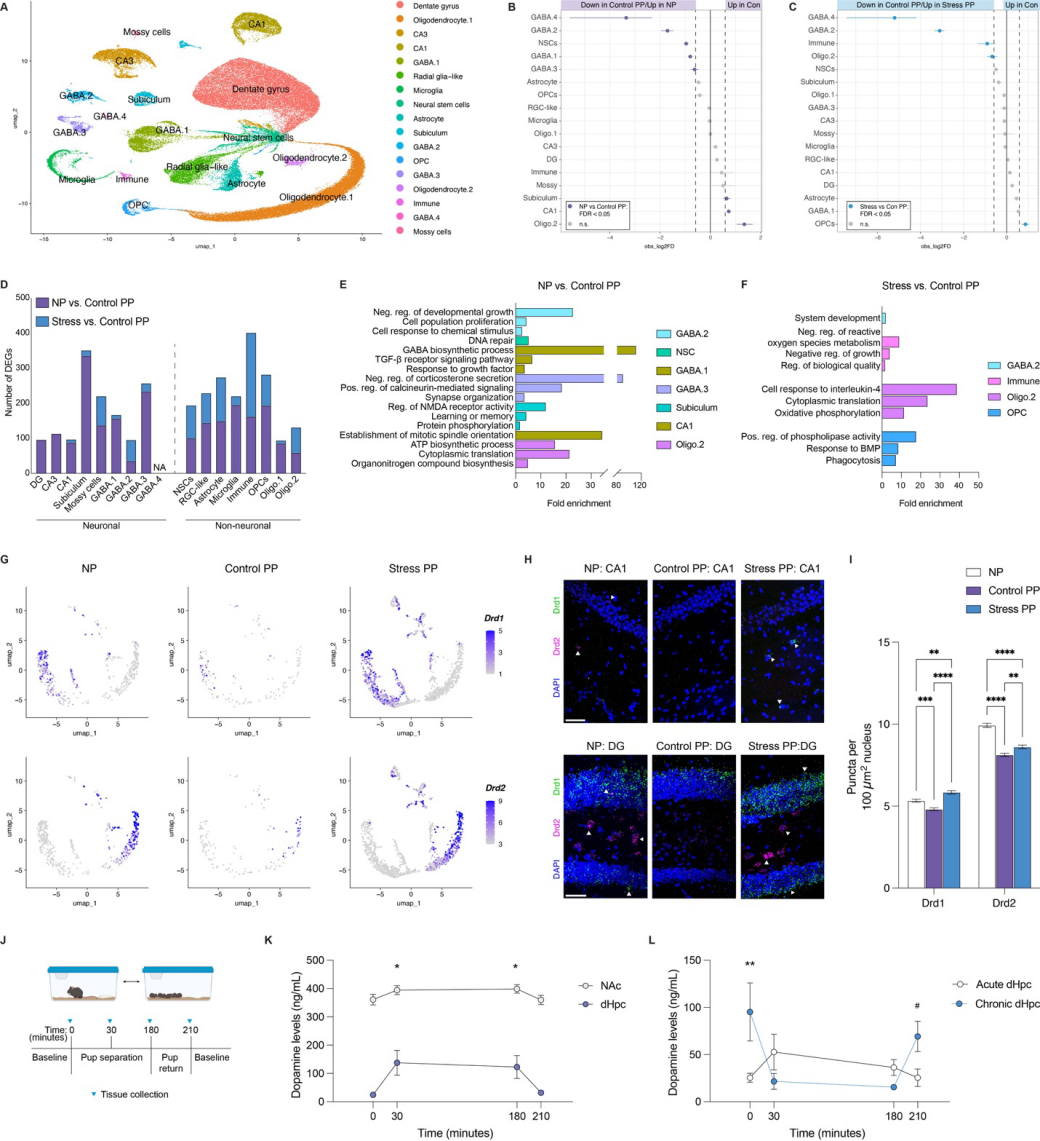
Parity-driven dHpc plasticity is suppressed by elevated dopamine signaling. **A)** UMAP representation of cell clusters (109,334 total cells) from dHpc tissues, colored by major cell types (N = 6 NP, 4 Control PP, 5 Stress PP). **B, C)** Point range plots depicting significant changes in proportions of major cell-type clusters between **(B)** NP vs. Control PP, and **(C)** Stress PP vs. Control PP (FDR < 0.05 and |log_2_Differerence| > log_2_(1.5)). **D)** Number of DEGs observed for each major cell-type following pseudobulk analysis (p < 0.05 and |log_2_(Fold Change)| > log_2_(1.5)). **E, F)** GO term analyses (Biological Process) for DEGs identified in cell clusters with significant proportional differences between **(E)** NP vs. Control PP, and **(F)** Stress vs. Control PP (FDR < 0.05). **G)** Subclustering of GABA.2 neuronal population revealed reductions in *Drd1* (top) and *Drd2* (bottom) expression in Control PP. **H)** Representative images of RNAScope for *Drd1* and *Drd2* mRNAs in dorsal CA1 (top) and dentate gyrus (DG, bottom). Scale bars, 50 μm. **I)** Quantification of *Drd1* and *Drd2* mRNA puncta in CA1 and DG nuclei (two-way ANOVA, main effect of group (F(2,82142) = 65.81, p < 0.0001), main effect of gene (F(1,82142) = 1771, p < 0.0001), interaction (F(2,82142) = 39.99, p < 0.0001); Tukey’s multiple comparisons test, **p < 0.01, ***p < 0.001, ****p < 0.0001). N = 5 animals/group. **J)** Experimental design of pup separation test to examine changes in brain tissue dopamine levels. **K)** Pup separation significantly increases dopamine levels from baseline in dHpc and NAc tissues (two-way ANOVA, main effect of time (F(3,52) = 4.287, p = 0.0089), main effect of brain region (F(1,52) = 225.7, p < 0.0001; interaction (F3,52) = 1.086, p = 0.3717), with significant increases in dHpc dopamine levels (Dunnett’s multiple comparisons test, *p < 0.05, **p < 0.01). N = 5–9 animals/time point. **L)** Chronic pup separations increases baseline dHpc dopamine concentrations (two-way ANOVA, interaction (F(3,53) = 4.822, p = 0.0048; group (1, 53) = 1.876), p = 0.1708, time (3, 53) = 1.736, p = 0.1706; Tukey’s multiple comparisons test, *p < 0.05, #p = 0.06). N = 5–12 animals/time point. Error bars represent mean ± SEM.

To elucidate the mechanisms underlying parity-induced dHpc plasticity, we next leveraged the transcriptomic and behavioral signatures shared between NP and Stress PP groups. This postpartum stress paradigm resolves key windows during which disruptions to parity adaptations occur, thereby providing greater temporal resolution of the mechanisms required to sustain these alterations. Building on our findings, we next performed single-nuclei (sn) RNA-sequencing on these three groups to identify the dHpc cell-types that integrate hormone and neurotransmitter signaling to drive parity-related plasticity. Following rigorous quality control assessments to remove confounding sources of variation, such as mitochondrial mapping percentage, doublets, and cell-type contamination (Fig. S4A–F), we retained 109,334 nuclei for downstream analysis (NP = 37,070; Control PP = 35,631; Stress PP = 36,633). Cell-type annotation was performed using both manual curation and label transfer from validated hippocampal datasets from the Broad Institute and Allen Brain Atlas^39,40^. This resulted in 16 distinct clusters (Fig. 4A, Fig. S4G–I), including excitatory neuron subtypes (dentate gyrus, CA1, CA3, subiculum, mossy cells; 43.3% of total), GABAergic neurons (GABA.1–GABA.4; 9.1% of total), neural progenitors (neural stem cells, radial glia-like cells; 9.7% of total), and non-neuronal/glial populations (astrocytes, oligodendrocytes, OPCs, microglia, immune cells; 37.9% of total).

Since our cell-type deconvolution analyses indicated downregulation of neuronal markers, we first assessed proportional differences across major cell-types. A Monte Carlo permutation test confirmed significant shifts in several populations beyond random variation, with GABA.2 and GABA.4 clusters being reduced in Control PP dHpc compared to both NP and Stress PP (Fig. 4B–C). We next used pseudobulk aggregation and differential expression analysis to identify DEGs within each annotated cluster (p < 0.05 and logFC > |1.5|). We observed transcriptional changes across both neuronal and non-neuronal subtypes, with excitatory neuron alterations predominantly observed between NP *vs.* Control PP, particularly within the subiculum (Fig. 4D). This pattern aligns nicely with enhanced contextual fear conditioning, as prior studies have implicated altered dHpc excitatory outputs in improved learning outcomes in dams^27^. In contrast, the most pronounced neuronal changes between Control and Stress PP were observed in the GABA.2 and mossy cell populations, alongside widespread DEGs across non-neuronal cell-types (Fig. 4D); note that differential expression analysis could not be performed for the GABA.4 cluster due to insufficient sample representation in multiple groups. GO term analysis of DEGs from cell-types displaying proportional differences revealed significant enrichment in pathways associated with responses to endogenous stimuli (GABA.2, GABA.1, GABA.3, OPC), cell proliferation (GABA.2, CA1, immune cells), synaptic function (GABA.3, subiculum), and metabolic processes (Oligo.2, GABA.1) across both comparisons (Fig. 4E–F).

Probing deeper into these altered cell-types, we focused on the GABA.2 cluster owing to shared proportional and transcriptional changes observed in comparison to both NP and Stress PP groups, which suggested a common mechanism limiting dHpc plasticity. Marker gene analysis revealed the significant enrichment of dopamine receptor D1 *(Drd1)* and D2 *(Drd2)* expression within this cluster compared to other cell-types (Fig. S4J), aligning with our prior bioinformatic analyses implicating dopaminergic regulation. To further resolve this population, we subclustered the GABA.2 cells and identified distinct subpopulations expressing *Drd1* and *Drd2*, both of which appeared to be altered in Control PP animals (Fig. 4G). In a separate cohort of mice, we validated these changes using RNA fluorescence in situ hybridization (FISH) in dorsal CA1 (Fig. 4H–I, Fig. S4K–L), where dopamine receptor-expressing interneurons have been previously described^41,42^. While dopaminoceptive inhibitory neurons were implicated in our data, we examined whether such changes extend to other dopamine-sensitive neuronal populations. RNA FISH in the dentate gyrus, which contains excitatory *Drd1* and *Drd2*-expressing neurons (in granule and hilar mossy cells, respectively; Fig. S7A), revealed significant group differences, alongside expected regional variations consistent with the sparser distribution of dopamine receptor-expressing neurons in CA1^43^ (Fig. 4H–I, Fig. S4M–N).

Since these findings established dopaminergic signaling as a potential key mediator of persistent parity-induced adaptations, this prompted us to examine whether maternal stress disrupts this process within its defined postpartum window. Given prior evidence that acute pup separation elevates dopamine levels in NAc^44,45^, we first investigated whether a similar response occurs in dHpc. Brain tissues were collected from 10 dpp dams at baseline, 30-minutes, and 3-hours post-separation, as well as 30-minutes post-reunion (Fig. 4J). We observed robust increases in dopamine levels in both NAc and dHpc during separation, with expected regional differences reflecting the strength of innervation to these regions (Fig. 4K). Next, we reasoned that this initial pup separation may reflect a normal, adaptive maternal response to unexpected offspring removal. Since acute and chronic stress produce different biological responses and behavioral outcomes, this prompted us to next assess the impact of repeated separation stress on dopamine modulation in these brain regions. Indeed, our data revealed distinct dopaminergic responses following acute *vs.* chronic separations, with repeated stress leading to loss of dopamine increases observed in response to pup separation, as well as elevated baseline dopamine in both dHpc and NAc tissues (Fig. 4L, Fig. S4O). Loss of induction following chronic stress may stem from elevated baseline levels, which facilitate engagement of autoreceptors and negative feedback mechanisms^46^. Similarly, increased release of dopamine in dHpc tissues were not observed in NP females 30-minutes following separation from a littermate (Fig. S4P), though this likely lacks the same salience as pup separation. These findings align well with our snRNA-seq observations, in which the reduced dopamine receptor expression resulting from parity reflects an adaptive process to reduce basal dopamine (supported by our transcriptional data in Figs. 2 and 3 showing downregulation of dopamine processes), thereby enabling robust responsiveness to relevant stimuli. In contrast, elevated receptor expression in NP and Stress PP animals may be a downstream effect of greater basal dopamine tone, thereby dampening the efficiency of stimulus-dependent signaling.

### Chemogenetic suppression of dopamine release into dHpc recapitulates key features of persistent parity-induced plasticity

**Figure 5.**
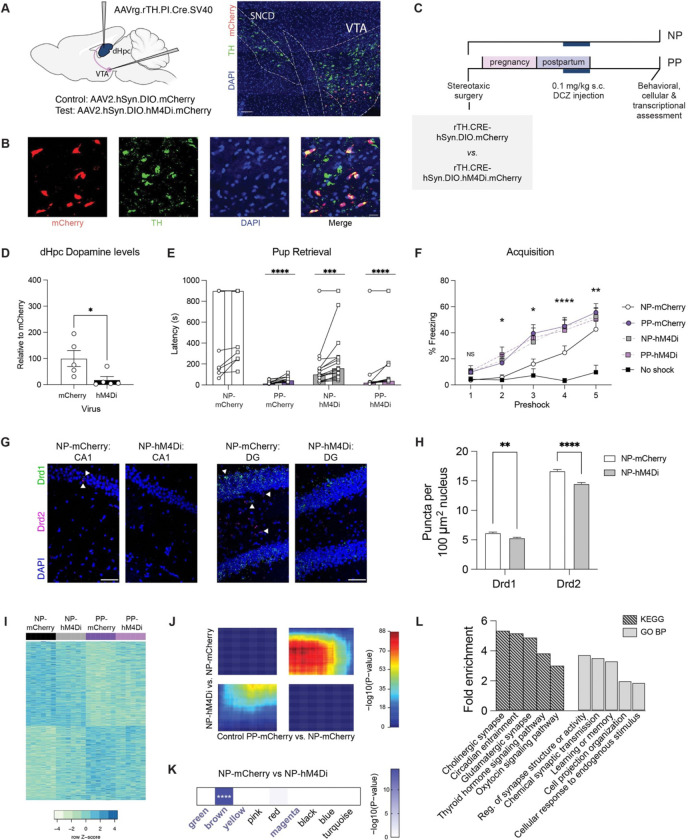
Chronic dopamine suppression is sufficient to phenocopy persistent maternal dHpc plasticity. **A)** Experimental design (left) and representative image (right) of AAV2.hSyn.DIO.mCherry targeting to VTA projection neurons following dHpc injection of retrograde (rg) Cre under control of the tyrosine hydroxylase (TH) promoter. Scale bar, 100 μm. **B)** Representative images showing overlap of virally-induced mCherry with TH+ cells in VTA. Scale bar, 20 μm. **C)** Timeline of stereotaxic surgeries and s.c. deschloroclozapine (DCZ) injections from 10–20 dpp in NP and PP female mice, followed by experimental assessments starting at 49 dpp. **D)** Significant reduction in dHpc dopamine levels following inhibition of VTA projection neurons (Student’s t-test:t(8) = 2.477, *p = 0.0383). N=5/group. **E)** PP-mCherry, NP-hM4Di, and PP-hM4Di retrieved pups faster than NP-mCherry (two-way rmANOVA, main effect of group (F(3,47) = 14.39, p < 0.0001), main effect of pup number (F(1, 47) = 18.96, p < 0.0001; Sidak’s multiple comparisons test, ****p < 0.0001, ***p < 0.001). N =11–15/group. Bar represents mean. **F)** PP-mCherry, NP-hM4Di, and PP-hM4Di freeze more compared to no shock controls at earlier phases of contextual fear acquisition, compared to NP-mCherry (two-way rmANOVA, main effect of group (F(4,41) = 4.739, p = 0.0031), main effect of preshock (F(2.818,115.5) = 61.92, p < 0.0001), interaction (F(16,164) = 2.606, p = 0.0012; Tukey’s multiple comparisons test, *p ≤ 0.05, **p < 0.01, ****p < 0.0001). N = 9–12/group. Error bars represent mean ± SEM. **G)** Representative images of RNAScope for *Drd1* and *Drd2* mRNAs in dorsal CA1 (top) and DG (bottom). Scale bars, 50 μm. **H)** Quantification of *Drd1* and *Drd2* mRNA puncta in CA1 and DG nuclei (two-way ANOVA, main effect of group (F(2,33404) = 64.43, p < 0.0001), main effect of gene (F(1,33404) = 2770, p < 0.0001), interaction (F(2,33404) = 12.52, p = 0.0004); Sidak’s multiple comparisons test, **p < 0.01, ****p < 0.0001). N = 3 animals/group. **I)** Bulk dHpc differential expression profiles of top 500 genes (by ascending p-values) between NP-mCherry and PP-mCherry. N=6/group. **J)** Threshold-free comparison by rank-rank hypergeometric overlap, showing concordant gene regulation (bottom left and upper right quadrants) between the comparisons indicated. **K)** Overlap of DEGs (NP-hM4Di vs. NP-mCherry, adj. p < 0.05) with gene co-expression modules (****p < 0.0001). High parity sensitivity modules are indicated in bold. **L)** Select GO terms and KEGG pathways significantly enriched for NP-mCherry vs. NP-hM4Di DEGs (FDR < 0.05).

Based on these observations, we hypothesized that the maintenance of low basal dopamine tone may drive adaptations of the dopamine-sensitive machinery in dHpc to promote long-term behavioral plasticity. To assess this, we chemogenetically suppressed the dopaminergic VTA projection to dHpc by bilaterally expressing a floxed inhibitory neuronal hM4Di-DREADD fused to mCherry (*vs.* floxed mCherry controls) in the VTA, coupled with retrograde AAVs expressing a tyrosine hydroxylase (TH)-dependent Cre recombinase into the dHpc (Fig. 5A). Selection of the VTA was guided by our findings that pup separation stress drives coordinated regulation in the dHpc and NAc, along with its established role as a key upstream modulator of dopaminergic signaling in the maternal NAc^47^ (also supported by our data; Fig. S4Q). We validated this approach by confirming selective mCherry expression in TH+ neurons of the VTA, as well as reduced *Fos* expression following administration of the selective DREADD agonist deschloroclozapine (DCZ; Fig. 5B, Fig. S5A–B.) To minimize injection-related stress in this paradigm, we sought to restrict the window of chronic DCZ administrations. Since maternal stress from 10–20 dpp was sufficient to disrupt parity-induced plasticity, we posited that dHpc dopamine suppression during this period may correspondingly be sufficient to promote neuronal adaptations in virgin NP (NP-mCherry, NP-hM4Di; Fig. 5C). As our comparisons aimed to determine if chronic dopamine reduction in virgin females can phenocopy parity-induced adaptations, we administered DCZ to postpartum dams in parallel (PP-mCherry, PP-hM4Di).

After confirming that our approach effectively suppressed dHpc dopamine (Fig. 5D) – and did not induce gross metabolic changes (Fig. S5C) – we assessed behavioral outcomes 1-month after the final DCZ injection (49 dpp). In the pup retrieval test, NP-hM4Di females exhibited significantly reduced latency to retrieve pups compared to NP-mCherry, with no differences observed relative to PP dams (Fig. 5E). Notably, while 8/13 NP-mCherry females failed to retrieve either pup, this occurred in only 1/15 NP-hM4Di animals, comparable to PP-mCherry (0/12) and PP-hM4di (1/11) dams. In contextual fear conditioning, NP-hM4Di females demonstrated enhanced acquisition, mirroring the heightened learning observed in PP dams (Fig. 1F). In the context recall test, NP-hM4Di females showed no significant differences from PP dams (Fig. S5D), though subtle effects were observed, suggesting that additional neuromodulatory mechanisms may interact with dopamine to modulate the consolidation and retrieval of conditioned fear responses underlying maternal behavioral plasticity.

Next, we examined whether such dopamine suppression influences cellular receptor expression in the dHpc. Similar to Control PP, NP-hM4Di females exhibited sustained downregulation of *Drd1* and *Drd2* in both CA1 and dentate gyrus (Fig. 5G–H, Fig. S5E–H), thus highlighting the lasting impact of prior dopamine modulation on dHpc receptor dynamics. Finally, we conducted transcriptional profiling of the dHpc in virally infected groups to assess the degree to which dopamine suppression mirrors parity-associated gene expression patterns. Comparing NP-mCherry *vs.* PP-mCherry expression profiles from bulk RNA-seq data, we observed an intermediate expression pattern in NP-hM4Di dHpc tissues (Fig. 5I, Table S7). RRHO analysis further supported transcriptional concordance between PP-mCherry and NP-hM4Di groups in comparison to NP-mCherry (Fig. 5J). To next assess whether the long-term transcriptional effects of prior baseline dopamine suppression align with changes in parity-associated transcriptional programs, we compared NP-mCherry *vs.* NP-hM4Di DEGs to previously identified parity-sensitive modules. Consistent with our behavioral findings, DEGs from NP-hM4Di overlapped with the brown module but not other parity-sensitive modules (Fig. 5K), thereby highlighting a specific mechanistic contributor to behavioral adaptations within the broader transcriptional and neuromodulatory landscape shaped by parity. Functional annotation of these DEGs revealed enrichment in pathways governing synaptic plasticity and responses to endogenous stimuli (Fig. 5J), which mirrored those found to be altered in Control PP dHpc and reinforced dopamine modulation as a key driver of long-lasting maternal brain adaptations (Fig. S5I).

## DISCUSSION

Despite decades of evidence that matrescence induces long-lasting behavioral adaptations across species, the mechanisms underlying these phenomena remain poorly understood. Through controlled brain-wide, time-course, and cell type-specific transcriptomic analyses – paired with robust behavioral outputs, – we identified gene networks, reproductive events, and neuromodulatory pathways that dynamically shape regional sensitivity across pregnancy and postpartum experiences, thereby promoting plasticity long after these stages have ended. Furthermore, using a maternal-pup separation paradigm during postpartum, we found that modulation of dopamine dynamics is essential for sustaining long-term neural and behavioral adaptations in maternal dHpc. Given dopamine’s role in reinforcing maternal behavior and reward, the observed downregulation of dopamine pathways in maternal brain was unexpected, though supported by a prior study^48^. Interestingly, reducing baseline dopamine may enhance the signal-to-noise ratio (SNR) of maternal responses to relevant cues, whereas elevated baseline dopamine levels may impair this process. Indeed, in this study we established the necessity and sufficiency of maintaining appropriate dopamine tone for parity-related dHpc plasticity, demonstrating that chronic dopamine elevation through maternal stress, and chemogenetic suppression within virgin dHpc, bidirectionally modulate parity-related changes at transcriptional, behavioral, and cellular levels. Such findings thus implicate dopamine as a key driver of long-term maternal brain remodeling. These findings expand upon the prevailing view that maternal dopamine function is solely driven by transient surges during maternal behaviors, instead revealing a concurrent downregulation of dopamine tone in critical regions, such as the dHpc, to optimize stimuli sensitivity and behavioral responsiveness.

### Ethological significance of maternal dHpc plasticity

Why the dHpc? Improvement of classic dHpc functions, such as spatial cognition, represents evolutionarily advantageous modes for maternal success by enabling more efficient resource foraging and nest navigation^49,50^. Additional dHpc functions, including novelty detection and sensory integration, may also heighten sensitivity to salient environmental cues that are essential for offspring survival^51,52^. Indeed, heightened hippocampal activation in response to one’s own infant’s cry in human fMRI studies underscores the diverse functional adaptations of this region^53^, suggesting a broad range of dHpc modifications that may converge to enhance maternal success. Although chronic maternal stress disrupts the normal trajectory of dHpc adaptations, it remains unclear whether such disruptions are maladaptive or represent evolved coping mechanisms in response to repeated separations. Furthermore, whether stress effects arise from reduced pup interactions, chronic stress exposures, or increased licking, grooming, and/or nursing upon pup reunion is difficult to disentangle within the current model^54^. Notably, unexpected parenting disruptions and resulting adjustments generate significant stress, with parenting-related psychosocial stress uniquely contributing to perinatal and postpartum disorders^55^. Moreover, as pup separation only reduces total pup exposure by ~6.5%, it is unlikely that reduced pup interactions alone account for these effects. Notably, chronic restraint stress during pregnancy similarly disrupts parity-induced spatial memory enhancements^27^. Moreover, human studies show that maternal early life stress alters hippocampal responses to infant cues^56,57^, supporting chronic stress as a common interferer in the normal trajectory of maternal dHpc neuroadaptations.

Interestingly, we observed natural variation in maternal responses to both acute and chronic stress, as reflected by variability in dHpc transcriptional changes, behavioral outcomes, and dopamine release following pup separation. This suggests that individual differences in maternal motivation or caregiving may influence dopaminergic responses to pup separation, as previously reported^45,58^. Interestingly, while the spectrum of outcomes in Stress PP dams indicate variability in stress effects, corticosterone levels were uniformly elevated across all dams following pup separation. This suggests that while chronic stress perturbs parity adaptations, these disruptions are mediated by individual dopamine dynamics downstream of maternal stress responses.

### Dopaminergic regulation of parity adaptations

Our findings, which reveal largescale transcriptional re-programming across the maternal brain, suggest that regions beyond the dHpc also undergo dopamine-mediated adaptations. Indeed, pup separation similarly increases dopamine release in the NAc^44,45^, while chronic separations dysregulate this induction by elevating basal dopamine concentrations, thereby facilitating engagement of negative feedback mechanisms^46^. Importantly, this perturbation offers insight into normal maternal dopamine regulation, emphasizing the need for low postpartum dopamine tone to enable signaling induction to relevant stimuli^27,59^. Moreover, as restraint stress enhances dopaminergic burst firing, and chronic gestational restraint impairs spatial learning, we propose that dopamine dysregulation serves as a common mode of dysregulating parity-related neuroadaptations across reproductive stages.

How do postpartum alterations drive sustained behavioral changes? Our findings reveal that downregulation of dopamine transcriptional networks in Control PP dHpc corresponds with reduced *Drd1/Drd2* expression, whereas elevated baseline dopamine in Stress PP associates with increased receptor expression, likely reflecting compensations to tonic dopamine dynamics during matrescence and/or chronic stress, rather than loss of dopaminoceptive cells. Notably, receptor downregulation may also contribute to structural plasticity, as parity is linked to dendritic remodeling in CA1 and CA3^7^, paralleling gray matter reductions observed in mothers years after parturition^1,2,4,22^. Thus, our work points to two mechanisms potentially underlying maternal dHpc adaptations: alterations in *Drd1/Drd2* receptor expression and shifts in baseline dopamine tone.

Given that *Drd1/Drd2*-expressing GABAergic neurons contribute to both aversive contextual memory formation and maternal behavior^45,60–64^, receptor expression changes likely intersect with these functions. Alternatively, receptor shifts may not directly influence behavior but instead reflect downstream consequences of altered dopamine tone. Within this framework, maintenance of low dopamine tone in Control PP dHpc may serve to reduce background noise, enhancing the processing of relevant cues by increasing SNR^65^. In addition to limiting negative feedback, low dopamine tone also prolongs and amplifies phasic dopamine responses to stimuli^66^. As phasic dopamine responses are essential for memory formation^67,68^, this process may underlie parity-induced enhancements in fear conditioning outcomes. While not traditionally considered a maternal behavior-regulating region, the dHpc integrates sensory, novelty, and salience information to shape pup retrieval responses. Consistent with our findings, lesion studies corroborate hippocampal involvement in pup retrieval behavior^69,70^. Moreover, hippocampal activity modulates downstream circuits via the polysynaptic VTA-hippocampal loop, which projects to the NAc and ventral pallidum - key regulators of pup retrieval^52,63^. Enhanced SNR within the dHpc may thus enable more efficient processing of offspring cues, facilitating robust behavioral responses to both rewarding and aversive stimuli through signal processing across this broader network.

These findings expand upon a broader maternal neuroadaptive process in which enhanced SNR optimizes responsiveness to offspring-related stimuli. Similar mechanisms have been described for oxytocin signaling, which enhances sensory salience by increasing SNR across multiple brain regions^71^. For example, in the left auditory cortex, oxytocin disinhibits pyramidal neuron activation in response to pup calls, enabling pup retrieval behaviors in previously non-maternal virgins^72^. In humans, disinhibition of cognitive and affective regions has also been associated with more responsiveness in maternal brains^73^. Similarly, estradiol and progesterone modulate SNR by reducing baseline activity of galanin-expressing mPOA neurons, leading to a reduced depolarization block that enhances responsiveness to pup stimuli^18^. Although these effects occur acutely in response to pregnancy hormones, they persist beyond postpartum^18^, mirroring the long-term neural adaptations observed in our study.

### Broader implications of maternal brain remodeling

While our study focused largely on dopamine, it is notable that our chemogenetic manipulations recapitulated key transcriptional and behavioral outcomes of parity, albeit not to the same extent. This underscores dopamine as a crucial but not exclusive driver of parity-induced adaptations. Indeed, our data also implicate hormonal contributions, including estrogen, progesterone, and oxytocin signaling, in shaping transcriptional programming. Consistent with this, previous studies have linked parity to estradiol-driven neuronal alterations^12^, while others have demonstrated that blocking oxytocin signaling disrupts parity-induced spatial learning enhancements^30^. These findings suggest that parity programming arises from integration of multiple neuromodulatory pathways to induce long-term effects. However, given that estrogen signaling during postpartum regulates oxytocin release, which in turn modulates VTA signaling, we propose that dopamine adaptations serve as a key downstream mechanism within a broader network driving sustained maternal neuroplasticity^74^.

What upstream mechanisms may regulate dopamine adaptations in maternal brain? As discussed, oxytocin regulates VTA dopamine release, yet whether it contributes to enduring changes in VTA-dHpc tone remains unknown. Additionally, the mechanisms by which chronic maternal stress alters this dopaminergic tone warrant further investigation. While this study focused on the VTA as an upstream provider of dHpc dopamine, the LC also provides substantial dopaminergic input to the dHpc, suggesting broader brain region involvement in dopamine-mediated adaptations^68,75^. Given its additional involvement in stress signaling, the LC, alongside the VTA, may contribute to signaling processes underlying chronic maternal stress effects. Furthermore, other brain regions, such as hypothalamic inputs, may modulate dHpc dopamine tone in response to stress, representing important avenues for future investigation.

Together, this work demonstrates that transient neuromodulatory processes during pregnancy and postpartum drive lasting maternal neuroplasticity. Importantly, this fundamental shift in brain state may interact with future experience-dependent plasticity. As parity is implicated in both risk and resilience to brain disorders^15^, future research should account for parity status as a key variable shaping differential outcomes, particularly in its interactions with other risk factors. In particular, our findings highlight an interplay between parity and stress, a key risk factor for brain disorder vulnerability, in shaping maternal brain outcomes. Thus, this study provides novel insights into the gene networks, dynamics, and neuromodulatory pathways that drive long-lasting parity-induced adaptations, while underscoring the importance of stress mitigation during pregnancy and postpartum.

## Supplementary Material

Supplement 1

Supplement 2

## INCLUSION & ETHICS STATEMENT

All collaborators associated with this work have fulfilled the criteria for authorship required by Cell Press journals. To obtain authorship, their participation in this study was deemed to be essential for the design and implementation of the work presented. Roles and responsibilities were agreed upon among collaborators ahead or during the research.
