# Supplementary material for: Persistent dopamine-dependent remodeling of the neural transcriptome in response to pregnancy and postpartum
Source: bioRxiv. 2025 Jun 2:2025.02.20.639313. Originally published 2025 Feb 25. Preprint. [Version 2] doi: 10.1101/2025.02.20.639313 (PMC11888212; doi:10.1101/2025.02.20.639313)
Supplement: 1 [file NIHPP2025.02.20.639313V2-supplement-1.pdf]

bioRxiv preprint doi: https://doi.org/10.1101/2025.02.20.639313; this version posted June 2, 2025. The copyright holder for this preprint (which was not certified by peer review) is the author/funder, who has granted bioRxiv a license to display the preprint in perpetuity. It is made available under aCC-BY 4.0 International license.

SUPPLEMENTARY MATERIALS

Materials and Methods
Extended Data Figs. 1 to 7
Extended Data Tables 1 to 9

bioRxiv preprint doi: https://doi.org/10.1101/2025.02.20.639313; this version posted June 2, 2025. The copyright holder for this preprint (which was not certified by peer review) is the author/funder, who has granted bioRxiv a license to display the preprint in perpetuity. It is made available under aCC-BY 4.0 International license.

MATERIALS AND METHODS

Human subjects

Brain tissue used in this study was provided by the Douglas-Bell Canada Brain Bank (DBCBB; www.douglasbrainbank.ca; RRID:SCR_025991). Informed consent from next-of-kin was obtained for each individual included in this study. Psychological autopsies, considered the gold standard for obtaining information on deceased individuals[66,67], were conducted. Briefly, these consist of a series of proxy-based, structured interviews assessing psychopathology with next-of-kin and complemented by reviews of medical records, as previously described[66]. Groups were matched for depression diagnosis, and were otherwise neurotypical individuals who died suddenly without prolonged agonal periods and did not have evidence of axis I disorders. Groups were matched for postmortem interval (PMI), tissue pH, and RNA Integrity number. Frozen histological grade samples of gray and white matter were dissected from the subiculum by expert neuroanatomists and stored at –80 °C. Dissections were performed on 0.5 cm-thick coronal sections with the guidance of a human brain atlas[68] (see also http://www.thehumanbrain.info/brain/bn_brain_atlas/brain.html). Subiculum samples were obtained from sections equivalent to plate 43 of the atlas (level of lateral geniculate nucleus), by dissecting through the hippocampal fissure with a slight upward angle, up to the beginning of the CA1 region.

Animals

Wild-type C57BL6/J mice were purchased from Jackson Laboratories at 8-weeks old, and maintained on a 12-h/12-h light/dark cycle throughout the entirety of the experiments. Mice were provided with *ad libitum* access to water and food throughout the entirety of the experiments. All behavioral testing occurred during the animals' light cycle. Experimenters were blind to

bioRxiv preprint doi: https://doi.org/10.1101/2025.02.20.639313; this version posted June 2, 2025. The copyright holder for this preprint (which was not certified by peer review) is the author/funder, who has granted bioRxiv a license to display the preprint in perpetuity. It is made available under aCC-BY 4.0 International license.

experimental group, and the order of testing was counterbalanced during behavioral experiments. All animal procedures were performed in accordance with NIH guidelines and with the approval of the Institutional Animal Care and Use Committee of the Icahn School of Medicine at Mount Sinai.

Breeding

Adult virgin female mice were pair bred in-house with age-matched males. Males were removed after a maximum of 5 days, and pregnant females were singly-housed at least 2 days before parturition. Pups were counted on the day of birth (0 dpp) and weaned at 21 dpp. Only dams with litters between 4-10 pups were used for all experiments. On the day of weaning, dams were group-housed into cages of 3-5 mice with animals of the same experimental condition. For timed breedings, copulation plugs were checked every morning within 1-hour after lights on, where confirmation of a plug was designated as E0.5, signaling the immediate removal of the female to her own cage with a nestlet. Virgin NP females were age-matched for each experimental cohort. Mating-experienced NP females were confirmed for the presence of a copulation plug. For Pregnancy Only dams, pups were removed at 0 dpp within 3 hours after lights-on to minimize maternal-offspring interactions. For comparison of PP dams to pup sensitized virgin females, litters were culled to 4 pups to equate litter size with the number of pups used for each sensitized female. In all other experiments, litters were not culled.

Postpartum stress paradigm

Stress PP females were subjected to limited nesting and maternal separation from 10-20 dpp, as previously described[69–71]. On each day of separation, the entire litter was removed to a clean cage with Sani-Chip bedding for 3-4 hours. Separations occurred during the light cycle, and the timing

bioRxiv preprint doi: https://doi.org/10.1101/2025.02.20.639313; this version posted June 2, 2025. The copyright holder for this preprint (which was not certified by peer review) is the author/funder, who has granted bioRxiv a license to display the preprint in perpetuity. It is made available under aCC-BY 4.0 International license.

varied each day to minimize predictability and acclimatization. EnviroDri nesting material was depleted to 1/3 of control cages during the days of separation. Following pup weaning on 21 dpp, the nesting material was restored to normal levels, and dams were group-housed into cages of 3-5 with animals of the same experimental condition.

Pup sensitization

Pup sensitization was conducted as previously described[22,72]. On the first day of pup sensitization, virgin NP females were presented with four pups (postnatal day 5) from a cage consisting of a lactating donor dam and her litter. Donor pups were exchanged for satiated pups from the same litter every 8-12 hours for 21 days. Donor pups were weighed daily to ensure continual weight-gain throughout the experiment, and only pups that exhibited consistent weight-gain were used. Behavioral observations were conducted during the first four days of sensitization. Each dam was observed for 30 minutes during the light phase for the following maternal behaviors: licking/grooming, crouching, and nestbuilding. During these observation periods, females were closely monitored to ensure that they did not display aggressive behavior toward the donor pups. On the last day of sensitization, donor pups were weaned from the cage, and sensitized females were group-housed into cages of 3-5 mice with animals of the same experimental condition.

Brain tissue collections

Animals were sacrificed by rapid decapitation. Whole brains were flash frozen with cold 2-methylbutane and stored at −80°C until further processing. Flash-frozen brains were sectioned at −20°C using a 1 mm mouse coronal brain matrix (Stoelting Co.). Tissues enriched for the brain region of interest were micropunched using a hollow needle (Ted Pella) according to the Allen Brain Atlas (see **Fig. S1A**).

bioRxiv preprint doi: https://doi.org/10.1101/2025.02.20.639313; this version posted June 2, 2025. The copyright holder for this preprint (which was not certified by peer review) is the author/funder, who has granted bioRxiv a license to display the preprint in perpetuity. It is made available under aCC-BY 4.0 International license.

Behavioral analyses

All animals (18-30 weeks, depending on the time from pup weaning) were handled for 2 minutes for two consecutive days prior to initial behavioral testing. Animals were habituated to the testing room for 1 hour prior to each behavioral assay. All testing occurred during the light phase.

Pup retrieval: All animals were individually housed for 24-hours prior to testing. Following habituation, 2 pups from a donor litter with a lactating dam (aged 4-6 dpp) were placed in different corners opposite to the nest in the home cage. Pup-directed interactions were recorded from above for 15-minutes or until both pups were successfully retrieved into the nest. Retrieval latency was calculated as [time pup was first placed into the nest by animal - time first pup was placed into the cage by experimenter].

Contextual fear conditioning: On day 1, mice were habituated for 10 minutes to the testing chamber, which consisted of a square plexiglass box with a metal grid inside a sound-attenuating cabinet wiped with 70% ethanol (Med Associates, VT). On day 2, following a 3-minute baseline measurement, mice were trained with five 2.0-second 0.7 mA foot shocks delivered with an intertrial interval of 90-seconds. Testing for conditioned fear responses (freezing) for a total of 5-minutes occurred 24-hours following training. Freezing was measured using ANY-maze software connected to a camera positioned above the testing chamber. Freezing is expressed as a percentage of the total test time or as a percentage of the 60-seconds prior to each shock during conditioning. Animals with freezing levels exceeding 40% prior to the first shock were excluded to remove potential confounding effects of heightened baseline responsivity that could interfere with accurately assessing conditioned fear.

bioRxiv preprint doi: https://doi.org/10.1101/2025.02.20.639313; this version posted June 2, 2025. The copyright holder for this preprint (which was not certified by peer review) is the author/funder, who has granted bioRxiv a license to display the preprint in perpetuity. It is made available under aCC-BY 4.0 International license.

Open Field: Mice were placed in a 16x16 square arena under dim lighting for 5 minutes. A camera positioned overhead recorded the total distance and time spent in the center *vs.* periphery using Ethovision software.

Object location task: For training, animals were allowed to freely explore two identical objects placed equidistant from adjacent corners of a 16x16 cm square arena for 5 or 10 minutes, before being returned to the home cage. Following a 1-hour delay, the animals were returned to the arena for testing, during which time one object was moved to the opposing corner. During the test, the animals were allowed to freely explore the objects for 5-minutes. A camera positioned overhead recorded time spent exploring each object using Ethovision software. The discrimination score was calculated as [(time spent with moved object) – (time spent with unmoved object)/[(time spent with moved object) + (time spent with unmoved object)].

Light-dark box: Anxiety-like behavior was tested in an apparatus containing two interconnected 20x20 cm compartments (Omnitech Electronics Inc.). One compartment was illuminated during the session (“light” side), while the other was covered by an opaque black perspex lid (“dark” side). The distance, time spent, and number of crossovers in each compartment were automatically recorded by Fusion software during the 10-minute test.

Forced Swim Test: Mice were placed in a 4 liter glass beaker with 2 liters of room temperature water for 8 minutes. Each session was recorded and scored by a blinded observer. The total number of seconds that mice were immobile during the last 5 minutes of the test were recorded, as previously described[73].

bioRxiv preprint doi: https://doi.org/10.1101/2025.02.20.639313; this version posted June 2, 2025. The copyright holder for this preprint (which was not certified by peer review) is the author/funder, who has granted bioRxiv a license to display the preprint in perpetuity. It is made available under aCC-BY 4.0 International license.

Observation of pup-directed behaviors in the home cage: Confirmation of maternal behavior in pup sensitized females was conducted based on prior studies[74]. Animals were observed from the first to fourth day of pup sensitization, based on published work that maternal sensitization in C57BL6/J females occurs following 4-days of pup exposures[29,75]. Observations occurred in the light period in the first 30-minutes after pups were placed in the cage. Each animal was scored every 3-minutes, with the observed behavior recorded as one or more of the following categories: nestbuilding, grooming, sniffing, crouching, nursing, eating, drinking, self-grooming, no-contact. PP dams were scored for the first 4-days following birth (0-3 dpp) concurrently for comparison.

Estrous cycle testing

Vaginal samples were taken on each day of behavioral testing. 15 µL of sterile PBS was gently pipetted into the vagina and mounted on a glass slide. Vaginal smears were stained with crystal violet dye, washed twice with water, and cover slipped with glycerol. Three 20x images per sample were acquired on a light microscope. Estrous stage was determined using the pretrained network of EstrousNet[76], and confirmed afterwards by a trained experimenter based on cytology of nucleated, cornified, or leukocytic cells.

Viral Transduction

Mice were anesthetized with ketamine (100mg/kg) and xylazine (10 mg/kg) i.p. and positioned in a stereotaxic frame (Kopf instruments). Given the widespread changes in dopamine receptor expression across dHpF subregions, we did not restrict our viral manipulations to a specific subregion. 2 µl of retrograde AAV.rTH.PI.Cre.SV40 (titer ≥ 7×10^{12} vg/mL, Addgene #107788-AAVrg) was infused bilaterally into the dHpF at 0.2 uL/min using the following coordinates: 7° angle; anterior-posterior (AP) -2.2 mm, medial-lateral (ML) ±2.0 mm, dorsal-ventral (DV) -2.0

bioRxiv preprint doi: https://doi.org/10.1101/2025.02.20.639313; this version posted June 2, 2025. The copyright holder for this preprint (which was not certified by peer review) is the author/funder, who has granted bioRxiv a license to display the preprint in perpetuity. It is made available under aCC-BY 4.0 International license.

mm. 1 µl of pAAV-hSyn-DIO-mCherry (titer ≥ 4×10^{12} vg/mL, Addgene #50459-AAV2) or pAAV-hSyn-DIO-hM4D(Gi)-mCherry (titer ≥ 5×10^{12} vg/mL; Addgene #44362-AAV2) was bilaterally infused into the VTA using the following coordinates: 7° angle; AP -3.3 mm, ML ±0.9 mm; DV -4.6 mm. Needles remained in place for 7 minutes following injection to minimize virus diffusion. Viral validations were conducted at least 21 days post-surgery to allow for optimal viral expression and recovery.

Chemogenetic manipulation

To directly manipulate dopamine signaling in dHpF, we selectively targeted the VTA-dHpF projection during a window established as being critical for dopaminergic downregulation. Following 7 days of recovery, surgerized female mice were randomly assigned to NP or PP groups. PP females were pair bred with naïve male mice for a maximum of 5 days, and individually housed prior to parturition. From 10-20 dpp, PP females were injected subcutaneously with deschloroclozapine (DCZ, Tocris #7193) – to minimize off-target effects[77] - at 1ug/kg in 1% DMSO or vehicle (saline). NP females were injected with DCZ concurrently. Following weaning on 21 dpp, PP dams were group-housed with animals of the same experimental condition. Behavioral testing occurred beginning at 28-days post-weaning (49 dpp), and brain tissues were collected following contextual fear conditioning for further analyses.

RNAscope in situ hybridization and analysis

Fresh frozen brains were cut into 12-16 µm thick slices in the coronal plane with a cryostat (Leica CM3050-S), mounted on charged Superfrost Plus microscope slides (Thermofisher #P36934), and stored at -80°C until processing. Sections were post-fixed with 4% PFA for 1.5 hours at 4°C, and permeabolized with hydrogen peroxide (10 minutes RT) and Protease III (30 minutes RT). The

bioRxiv preprint doi: https://doi.org/10.1101/2025.02.20.639313; this version posted June 2, 2025. The copyright holder for this preprint (which was not certified by peer review) is the author/funder, who has granted bioRxiv a license to display the preprint in perpetuity. It is made available under aCC-BY 4.0 International license.

RNAscope Multiplex Fluorescent Reagent Kit v2 (ACD Bio) was used according to manufacturer's instructions to sequentially stain sections with the following probes: Drd1a (Mm-Drd1a-C1, #461901) and Drd2 (Mm-Drd2-C3, #406501-C3). RNAscope probes were visualized using TSA Vivid Fluorophores (Tocris) at 1:750 dilution. Sections were counterstained with DAPI (ACD Bio) and mounted using ProLong Gold Antifade Mountant (Thermo). Confocal images (3 images per animal, 1024 × 1024 pixels) were acquired on a Zeiss LSM 780 upright microscope using a 40X objective with Zen Black software, with 5x1 tiled images. Images were averaged across 8 consecutive acquisitions at a bit depth of 16 bits, with 2 z-stacks acquired per image. Subcellular quantification of individual puncta per 100 μm nucleus - identified by cellular detection of DAPI staining - was performed for each maximum intensity projected image using QuPath software (v.0.5.1)[78]. Regions of interest (CA1 and dentate gyrus) were annotated and determined by superimposing images onto the Allen Brain Atlas.

Immunofluorescence and analysis

Mice were anesthetized with isoflurane and perfused with cold 1×PBS followed by 4% PFA. Brains were post-fixed in 4% PFA overnight and then transferred to a 30% sucrose/PBS solution for 2 days. Brains were sectioned at 40 μm thickness using a Leica CM3050-S cryostat, with serial sections collected from the VTA. For each subject, 2–3 brain slices were blocked for 2 hours (0.1% Triton X-100, 10% normal donkey serum), followed by overnight incubation at 4 °C with primary antibodies: chicken anti-tyrosine hydroxylase (1:500, Aves Labs #TYH) and rabbit anti-Fos (1:2000, Synaptic Systems #226-008). The next day, slices were incubated for 2 hours at RT with fluorescent-conjugated secondary antibodies (donkey anti-chicken Alexa Fluor 488, Invitrogen #A78948; donkey anti-goat Alexa Fluor 680, Invitrogen #A10043). Slices were counterstained with DAPI (1:10000, Thermo Scientific #62248) and mounted with ProLong Gold Antifade

bioRxiv preprint doi: https://doi.org/10.1101/2025.02.20.639313; this version posted June 2, 2025. The copyright holder for this preprint (which was not certified by peer review) is the author/funder, who has granted bioRxiv a license to display the preprint in perpetuity. It is made available under aCC-BY 4.0 International license.

Mountant (Thermo Fisher #P36934). Confocal images (2-3 replicates per animal, 1024 x 1024 pixels) were acquired on a Zeiss LSM 780 upright microscope using a 40x objective with Zen Black software. Images were averaged across 8 consecutive acquisitions at a bit depth of 16 bits. Image analysis was conducted using FIJI software (NIH). The Fos and TH channels were thresholded using the MaxEntropy method to define regions of interest. Following identification of colocalized Fos+/TH+ signal, Fos intensity was measured and averaged from 4-6 images per animal. Brightness and contast were adjusted for representative images.

Dopamine ELISA

Brain tissue dopamine levels were assessed in response to 3 hours of pup separation in the home cage. Independent biological replicates were collected at each time point, with separate animals used for each measurement. Brains were rapidly harvested (see Brain Tissue Collection, above) at baseline (prior to pup removal, 0 min), during pup removal (30 and 180 min), and 30 min after pups were returned to the home cage (210 min). For assessment of NP females, samples were collected in the home cage 30-min following removal of a littermate. Equal weights of micropunched brain tissues were homogenized in lysis buffer (0.01 N HCl, 1 mM EDTA, 4 mM sodium metabisulfite). Tissue dopamine levels were assessed using the Dopamine (Research) ELISA Kit (ALPCO Diagnostics) according to manufacturer's instruction.

HPA axis assessment

Plasma corticosterone levels were assessed in response to 3-hours of pup separation in the home cage. Testing was initiated within 2-hours after lights on. Tail blood was collected prior to pup removal (0 min), during pup removal (30 and 180 min), and 120 min after pups were returned to the cage (300 min) from the same animals. Samples for control animals in the home cage, used to

bioRxiv preprint doi: https://doi.org/10.1101/2025.02.20.639313; this version posted June 2, 2025. The copyright holder for this preprint (which was not certified by peer review) is the author/funder, who has granted bioRxiv a license to display the preprint in perpetuity. It is made available under aCC-BY 4.0 International license.

account for the effects of handling-induced stress, were collected concurrently. Blood samples were immediately mixed with 50 mM EDTA and centrifuged at 5000 rpm for 10 minutes. Plasma was collected and stored at −80 °C until analysis. Corticosterone levels were quantified using a Corticosterone ELISA kit (ENZO Life Sciences) according to manufacturer's instruction.

Clonal *TGM2* Knockout in HeLa cells

HeLa cells (ATCC, CCL-2) were cultured at 37 °C with 5% CO_2 in DMEM medium (high-glucose, ThermoFisher 11965118) supplemented with 10% FBS (Sigma-Aldrich) and 500 U ml−1 penicillin and streptomycin. The CRISPR guide RNA targeting exon 5 of *TGM2* was purchase from IDT, containing the sequence ACGCTGGGACAACAACTACG. 120 pmol of guide RNA and 100 pmol of Alt-R™ S.p. Cas9 Nuclease V3 (IDT, 1081058) were premixed for 15 minutes in 5 µL total (with PBS). 200k HeLa cells were washed in PBS, before resuspending in 20 µL of nucleofector solution (SE Cell Line 4D-Nucleofector® X Kit S, Lonza V4XC-1032). The 5 µL Cas9/sgRNA mix was added, as well as 1 µL of Alt-R Cas9 Electroporation Enhancer (IDT, NC1395977), and all mixed gently. The entire mixture was transferred to a 16-well Nucleocuvette strip (Lonza, PDH-2104) gently, and nucleofected using the Lonza 4D-Nucleofector® X Unit, using the default settings for HeLa cells. Directly after nucleofection, 80 uL of pre-warmed culture media was added to the cuvette. The entire mixture was immediately transferred to a 6 well plate with 2 mL of prewarmed culture media, and incubated cultured at 37 °C with 5% CO_2 for 48 hours. Nucleofection efficiency was assessed by using a second positive control reaction with a pMax-GFP plasmid (Addgene, 177825). The pool of cells was diluted to a concentration of 1 cell per 200 uL, and 100 uL aliquoted into each well of 10 96 well plates, and cultured at 37 °C with 5% CO_2. After 14 days, single-clones were identified using a light microscope. Single-clone containing wells were expanded and targeting assessed by extracting genomic DNA and performing PCR and

bioRxiv preprint doi: https://doi.org/10.1101/2025.02.20.639313; this version posted June 2, 2025. The copyright holder for this preprint (which was not certified by peer review) is the author/funder, who has granted bioRxiv a license to display the preprint in perpetuity. It is made available under aCC-BY 4.0 International license.

PCR sequencing over the targeted site (Fwr: GGCTCCAGCCCCCACCATCTGCCGCAC, Rev: GCCACATAGCGCATTGAGAGTGTTGGT). PCR sequencing results were assessed using Synthego's ICE analysis. Clones which were identified as introducing premature stop codons were expanded further.

Assessment of *TGM2* KO Cell Line

Western Blot for TG2: Briefly, HeLa cells were collected and lysed using high-salt buffer (20 mM HEPES pH 7.9, 500 mM KCl, 10 mM MgCl2, and 1 % NP-40), followed by brief pulse-sonication. 100 ug of protein was run on a 4-12% Bis-Tris gel (Invitrogen, NW04122) for 45 minutes at 150V. Protein was transferred to a 0.2 µm nitrocellulose membrane using a Trans-Blot Turbo Transfer System (BioRad) following the manufacturers protocol. The membrane was blocked in 5% milk in TBS for 1 hour, and primary antibody (anti-TG2, Abcam 2386, 1:500 in 1.5% milk in TBS) overnight at 4° C. The blot was washed 3 x 15 minutes in TBS-T, and then incubated with secondary antibody (Goat anti-Mouse IgG (H+L) Cross-Adsorbed Secondary Antibody, Alexa Fluor™ 647, Invitrogen A-21235) at room temperature for 1 hour, followed by washing 3 x 15 minutes in TBS-T. The blot was imaged using a BioRad ChemiDoc MP, and then the blot was stained with amido-black stain to assess total protein loading.

Transamidation Activity Assay: 100 ug of HeLa extract was diluted in low-salt buffer (20 mM HEPES pH 7.9, 150 mM KCl, 10 mM MgCl2), and 1X final transamidation assay buffer was added (25 mM Tris-HCl, pH 8, 10 mM CaCl2, 10 mM DTT, 10 mM KCl). Biotin-cadaverine (Millipore Sigma A5348) was added to a final concentration of 1 mM, and reactions incubated at 30° C for 2 hours. A western blot was run as described above, using a Streptavidin-Alexa Fluor™ 488 Conjugate (ThermoFisher S32354) to measure incorporation of biotin-cadaverine.

bioRxiv preprint doi: https://doi.org/10.1101/2025.02.20.639313; this version posted June 2, 2025. The copyright holder for this preprint (which was not certified by peer review) is the author/funder, who has granted bioRxiv a license to display the preprint in perpetuity. It is made available under aCC-BY 4.0 International license.

Statistics

Statistical analyses for behavioral and immunoassay data were conducted using Prism software (GraphPad, v.10.4.1). Data distribution was assessed for normality. Data that met assumptions of normality were analyzed using parametric tests, while non-normally distributed data were analyzed using non-parametric alternatives. For experiments involving multiple conditions, one-way or two-way ANOVAs were performed, followed by *post hoc* analyses when appropriate. For time course analyses where multiple measurements were taken from the same animal, repeated measures ANOVAs were performed. Two-tailed Student's t-tests were used for comparisons between two conditions. Behavioral data derived from manual observations and pregnancy/litter outcomes were analyzed using chi-square tests. Grubb's test (alpha = 0.05) was applied to detect outliers where necessary. Statistical significance was defined as $p \leq 0.05$.

Bulk RNA-seq and analysis

RNA isolation and library preparation: Total mRNA was extracted from frozen brain tissues after homogenization in Trizol Reagent (Thermo Fisher) and cleaned using RNeasy Microcolumns (Qiagen) following the manufacturer's instructions. For RNA-seq library preparation, 150 ng of mRNA per sample was used with either the Illumina Stranded mRNA Prep Kit (Illumina, #20040534) or the TruSeq RNA Library Prep Kit v2 (Illumina, #RS-122-2001), according to the manufacturer's protocols. Library quality was assessed using a Qubit Fluorometer 2.0 (Thermo Fisher) and a High Sensitivity D5000 TapeStation assay (Agilent) before sequencing on a NovaSeq 6000 or NovaSeq X system.

Differential expression analysis: Raw fastq files, containing an average of 20–30 million reads per sample, were processed for pseudoalignment and abundance quantification using Kallisto (v.

bioRxiv preprint doi: https://doi.org/10.1101/2025.02.20.639313; this version posted June 2, 2025. The copyright holder for this preprint (which was not certified by peer review) is the author/funder, who has granted bioRxiv a license to display the preprint in perpetuity. It is made available under aCC-BY 4.0 International license.

0.46.1) against the Ensembl Mus musculus reference (v. 79)[79]. To filter lowly-expressed genes, only those with a total read count of at least 10 across all samples were retained. To account for unwanted variation among samples within each sequencing experiment that could arise from technical or biological factors unrelated to the conditions of interest (including litter size, estrous stage, day of sample collection, etc.), RUVs (v1.32.0) was applied with a negative control gene set derived from the total genes identified per sequencing experiment, after ensuring that unwanted variation did not correlate with covariates of interest, as described previously[80,81]. Differential expression analysis was performed using DESeq2 (v1.38.3)[82], with significant genes defined by an adjusted p-value < 0.05. For brain-wide transcriptome comparisons in which samples were processed across multiple sequencing runs and stemming from separate cohorts, pairwise comparisons were performed independently for each brain region. For all other experiments, where subjects came from the same cohort and were processed in a single sequencing run, all groups were analyzed together to maintain consistent normalization within the experiment. Gene expression time course analyses examining the periods before, during, and after pregnancy and postpartum were performed on normalized count data using the ImpulseDE2 package (v0.99.10)[31] for each brain region. Significant genes exhibiting transient regulation or monotonous changes in expression were identified using case-only differential expression analysis, with a Q-value threshold of 0.05.

Weighted gene co-expression analysis: To identify brain-wide gene co-expression networks, normalized count data for all brain regions were compiled and analyzed using the WGCNA package (v1.73)[20]. Co-expression networks were constructed from the 7,500 most variable genes, determined by ranking gene variance. A soft threshold power of 12 was identified with the pickSoftThreshold function to ensure scale-free network properties. Modules were identified

bioRxiv preprint doi: https://doi.org/10.1101/2025.02.20.639313; this version posted June 2, 2025. The copyright holder for this preprint (which was not certified by peer review) is the author/funder, who has granted bioRxiv a license to display the preprint in perpetuity. It is made available under aCC-BY 4.0 International license.

based on dissimilarity of a signed topological overlap matrix (TOM), and named with an arbitrary color. The "gray" module encompassed genes that did not segregate into any specific module, and was therefore removed from further analyses. To assess the enrichment of differentially expressed genes (DEGs) within each brain region per module, Fisher's exact tests were performed using the fisher.test() function in R (v4.3.0). To analyze the correlation between gene modules and brain regional sensitivity to parity, brain regions were categorized into two groups based on the top five regions with significant DEG overlap. These regions were designated as "High Sensitivity" regions, while all other regions were classified as "Low Sensitivity." Pearson correlation coefficients were calculated to examine the relationship between each module and the trait (regional sensitivity × parity status), with statistical significance determined by Student's t-tests ($p < 0.05$). Heatmaps were generated to visualize these module-trait correlations, and module-specific gene lists were exported for pathway enrichment analysis.

Pathway and predicted upstream regulator analyses: Functional annotation of DEGs was conducted using ShinyGO (v0.81)[83], with all protein-coding genes in the mm10 genome used as the background. Pathways with an FDR < 0.05 were considered significant. All significant pathways and associated statistics are provided in the Supplementary Tables. For figures, relevant pathways were selected from the top 10 or 1/3 of significant terms, ranked by FDR, to emphasize processes consistent with hypotheses informed by published literature. For GO term selection, Revigo[84] was used to reduce redundancy of overlapping GO terms when needed. Ingenuity Pathway Analysis (IPA; Qiagen, Inc., v.01-23-01) was used to predict upstream regulators for DEG lists[85]. For "High Sensitivity" and "Low Sensitivity" regions (see *WGCNA* section), DEGs shared by at least two or three brain regions were extracted, irrespective of the direction of change. The IPA software was used to identify upstream regulators associated with each DEG list, with

bioRxiv preprint doi: https://doi.org/10.1101/2025.02.20.639313; this version posted June 2, 2025. The copyright holder for this preprint (which was not certified by peer review) is the author/funder, who has granted bioRxiv a license to display the preprint in perpetuity. It is made available under aCC-BY 4.0 International license.

statistical significance defined as $p < 0.05$. To investigate the mechanisms underlying parity programming of dHpF plasticity, significant upstream regulators were systematically prioritized based on one or more of the following criteria: (1) multiple molecules involved in the same signaling pathways (e.g., progesterone and its receptor, PGR); (2) structurally similar molecules that engage analogous signaling cascades (e.g., levodopa and dopamine); (3) molecules known to be influenced by reproductive exposures; (4) molecules demonstrated to play a role in hippocampal plasticity; (5) molecules identified as significant in both "High Sensitivity" and "Low Sensitivity" regulator lists; and (6) molecules with high statistical significance values. Following selection, upstream regulators were categorized into general molecular classes (e.g., "Hormone," "Transcription Factor," "Lipid") and grouped for visualization. Data are presented as a bubble plot, with marker shape representing number of brain regions sharing DEGs and marker size corresponding to significance.

Gene expression overlap analyses: Jaccard indices were calculated for overlapping gene lists using the GeneOverlap package (v1.36.0)[86], with significance defined by $p < 0.05$. Fisher's exact tests were calculated using the base *fisher.test()* function in R (v. 4.3.0) following construction of contingency tables for each comparison. Transcriptome-wide, threshold-free gene expression overlap was visualized using Rank-Rank Hypergeometric Overlap (RRHO) heatmaps generated with the RRHO2 package (v1.0)[30]. Gene lists were ranked by signed p-values, calculated as the log10-transformed nominal p-value multiplied by the sign of the fold change, without applying differential expression thresholds.

Cell-type deconvolution: Brain cell-type proportion was estimated from normalized expression data using the BRETIGEA package (v.1.0.4)[18] with default markers. For comparisons between

bioRxiv preprint doi: https://doi.org/10.1101/2025.02.20.639313; this version posted June 2, 2025. The copyright holder for this preprint (which was not certified by peer review) is the author/funder, who has granted bioRxiv a license to display the preprint in perpetuity. It is made available under aCC-BY 4.0 International license.

groups, surrogate proportion variables were normalized using the Normalize function in Prism (GraphPad, v. 10.4.1). Each sub column was normalized separately, with the smallest value set to 0% and the largest value set to 100%. To calculate log_2(fold change) between groups, normalized values per cell-type per group were averaged and expressed as the log2 ratio of the group means.

Single nuclei RNA-seq

Nuclei isolation and library preparation: For each animal, 2 mm dHpF-enriched tissue micropunches were collected bilaterally from consecutive 1 mm slices from -0.80 to -2.80 mm relative to bregma for a total of 4 punches (Ted Pella). Samples were processed in batches of 4-6, with each group represented within each batch. Nuclei were isolated using a modified version of a sucrose density gradient isolation protocol[87]. Briefly, thawed tissues were placed in 1 mL of lysis buffer (0.32 M sucrose, 5 mM $CaCl_2$, 3 mM magnesium acetate, 0.1 mM EDTA, 10 mM Tris-HCl pH 8, 1 mM DTT, 0.1% Triton X-100) with 50 µL of 25 U/mL RNase inhibitor (Takara #2313B) in a dounce homogenizer (Wheaton #357538). Homogenization was performed with 20 strokes using a tight pestle. Another 1 mL of lysis buffer was added, followed by an additional 10 strokes. The resulting 2 mL homogenate was transferred to a 15 mL Open-Top Thinwall Polypropylene Tube (Beckman-Coulter #361707). The homogenizer and pestle were rinsed with 2 mL of lysis buffer, and this wash was combined with the homogenate for a total of 4 mL. The homogenate was carefully underlaid with 9 mL of sucrose solution (1.8 M sucrose, 3 mM magnesium acetate, 1 mM DTT, 10 mM Tris-HCl pH 8) and ultracentrifuged at 24,000 rpm for 1 hour at 4 °C using a Sorvall™ WX+ centrifuge. After centrifugation, the supernatant and debris at the interface were gently removed. The nuclear pellet was resuspended in 1 mL of resuspension buffer (0.02% bovine serum albumin in DPBS with 25 µL of 25 U/mL RNase inhibitor) and incubated on ice for 10 minutes. The suspension was passed through a 35 µm nylon mesh filter (Corning #352235) into a

bioRxiv preprint doi: https://doi.org/10.1101/2025.02.20.639313; this version posted June 2, 2025. The copyright holder for this preprint (which was not certified by peer review) is the author/funder, who has granted bioRxiv a license to display the preprint in perpetuity. It is made available under aCC-BY 4.0 International license.

1.5 mL RNase/DNase-free microcentrifuge tube and centrifuged at 2600 x g for 10 minutes at 4 °C. Supernatants were discarded, and nuclei were resuspended in 200 µL of resuspension buffer. A 10 µL aliquot of the nuclei suspension was stained with Trypan Blue to assess quality and concentration using a Countess 3 Automated Cell Counter. Nuclei suspensions were loaded onto a Chromium Single Cell 3′ chip (10X Genomics, v3) and processed according to the manufacturer's protocol, targeting 10,000 nuclei. Single-nuclei libraries were generated using the 10X Chromium Next GEM Single Cell 3′ v3.1 (Dual Index) protocol (CG000315 Rev A). Libraries were pooled, loaded onto a single 10B 100 Cycle Flowcell, and sequenced using an Illumina NovaSeq 6000 system to generate 25-30,000 paired-end 2 × 100 bp reads.

Data analysis: FastQ files were processed with the 10X Genomics Cell Ranger pipeline (v7.1.0) to demultiplex reads, align them to the mouse genome (mm10-2020-A), remove PCR duplicates, and generate gene expression matrices. Cell Ranger filtered outputs were analyzed using Seurat v4.3.0[88], and mitochondrial RNA content per cell was calculated using the GRCm39 (mm10) genome annotation and regressed out using SCTransform normalization protocol included in the Seurat toolkit with 20 principal components (PCs) and a resolution of 0.1. To estimate ambient RNA and correct for background contamination, the SoupX (v1.6.2) package[89] was used for each sample using raw and filtered feature matrices from the Cell Ranger output. Heterotypic doublets were identified and removed using DoubletFinder (v2)[90] to ensure the integrity of singlet datasets. Filtered singlet datasets were then re-normalized and integrated using the same Seurat SCTransform v2 workflow mentioned above. Cell clusters were annotated using a combination of expert curation based on published marker genes[37,91–96], and label transfer from hippocampal reference datasets, including the Allen Brain Map and Broad Institute resources[36,37]. Clusters with contaminant cell populations expressing markers for choroid plexus (*Ttr*), ependymal (*Tmem212*),

bioRxiv preprint doi: https://doi.org/10.1101/2025.02.20.639313; this version posted June 2, 2025. The copyright holder for this preprint (which was not certified by peer review) is the author/funder, who has granted bioRxiv a license to display the preprint in perpetuity. It is made available under aCC-BY 4.0 International license.

and vascular leptomeningeal cells (*Vtn, Col1a2*) were removed from the analysis[96–99]. Additionally, as the sequential 2mm micropunches encompassed portions of cortical, thalamic and vHpF regions, clusters characterized by enrichment of published non-dHpF neuronal markers using Seurat's FindMarkers function (layer 5/6 cortical: *Rorb, Foxp2*[99]; ventral granule neurons: *Tox3*[93]) were also removed from the analysis. Cell cluster proportion analyses were conducted using the scProportionTest package[100], which employs a Monte Carlo permutation test to evaluate whether observed differences result from random sampling. Proportional differences between conditions were compared to a null distribution generated by resampling, and statistical significance was determined by permutation-based p-values with confidence intervals estimated via bootstrapping. Differential expression analysis was conducted using pseudobulk analysis, where gene counts were summed across all cells within each sample for each cell type cluster using the AggregateExpression() function. DESeq2 was then applied at the sample level to conduct differential expression. To explore pathways underlying cluster-specific differences across conditions, pathway analysis was conducted using ShinyGO on genes meeting the following criteria: $\log_2 FC > 1.5$ and $p < 0.05$.

CUT&RUN-seq

Cleavage Under Targets and Release Using Nuclease: The procedure was adapted from established protocols[46,101]. For mouse brain tissue, two unpooled 1.5mm dHpF punches were used for each biological replicate and split across three reactions (H3K4me3Q5ser, H3K4me3, IgG). For human brain tissue, ~10mg of tissue dissected from individual frozen subiculum samples was collected from each subject, and split across the indicated antibodies. Samples were dounce homogenized in nuclear extract (NE) buffer (20mM HEPES-KOH pH 7.9, 10mM KCl, 0.5mM

bioRxiv preprint doi: https://doi.org/10.1101/2025.02.20.639313; this version posted June 2, 2025. The copyright holder for this preprint (which was not certified by peer review) is the author/funder, who has granted bioRxiv a license to display the preprint in perpetuity. It is made available under aCC-BY 4.0 International license.

spermidine, 0.1% Triton-X, 20% glycerol with protease inhibitors) and passed through a 21 gauge needle 10x. Nuclei were pelleted at 1,100g for 5 min at 4 °C in a swinging-bucket rotor, passed through a 40 µM strainer (pluriSelect USA), washed again in 500 µl NE buffer and counted. BioMag Plus Concanavalin A beads (Polysciences) were prepared per reaction by washing three times with binding buffer (20 mM HEPES-KOH pH 7.9, 10 mM KCl, 1 mM $CaCl_2$, 1 mM $MnCl_2$). Beads (15 µl) were aliquoted into 1.7 ml DNA low-bind tubes (Eppendorf) containing 500 µl NE buffer and 100,000 nuclei per reaction. Samples were rotated at room temperature for 10 min, then bead-bound nuclei were washed three times with wash buffer (20 mM HEPES pH 7.5, 150 mM NaCl, 0.1% Triton X-100, 0.1% Tween-20, 0.5 mM spermidine, 0.1% BSA, and protease inhibitors), resuspended in 100 µl antibody buffer (wash buffer with 2 mM EDTA), and mixed. 2 µl antibodies were added to the corresponding tubes: H3K4me3 (Active Motif, 39159), H3K4me3Q5dopaminyl (Millipore, ABE2590), or rabbit IgG (Invitrogen, 10500c). Samples were incubated overnight at 4 °C on a rotating mixer angled upward at 20 degrees. The next day, nuclei were washed twice with cold wash buffer, and incubated with 2.5 µl pAG-MNase (Epicypher, 15-1016) for 1 h at 4 °C. After four washes with cold wash buffer and one with low-salt rinse buffer (20 mM HEPES pH 7.5, 0.5 mM spermidine, 0.1% Tween-20, 0.1% Triton X-100), nuclei were resuspended in calcium incubation buffer (3.5 mM HEPES pH 7.5, 10 mM $CaCl_2$, 0.1% Tween-20, 0.1% Triton X-100) and placed into an ice-cold block at 4 °C. MNase digestion was stopped by adding 100 µl of 2x Stop Buffer (340 mM NaCl, 20 mM EDTA, 5 mM EGTA, 0.1% Tween-20, 0.1% Triton X-100, 25 µg/ml RNase A, and 0.05 ng/100 µl E. coli spike-in DNA), followed by a 15 min incubation at 37 °C without shaking. Beads were then placed on a magnet and 200 µl of supernatant was collected. DNA was purified using the Zymo ChIP DNA Clean & Concentrator kit (D5205), eluted in 30 µl, and stored at −20 °C for library preparation. Libraries were generated

bioRxiv preprint doi: https://doi.org/10.1101/2025.02.20.639313; this version posted June 2, 2025. The copyright holder for this preprint (which was not certified by peer review) is the author/funder, who has granted bioRxiv a license to display the preprint in perpetuity. It is made available under aCC-BY 4.0 International license.

using the NEBNext Ultra II DNA library kit, quantified using a Qubit fluorometer with the HS DNA kit, checked for size distribution on the Agilent TapeStation, pooled equimolarly, and sequenced on an Illumina NovaSeq X.

Data analysis: Raw fastq files were aligned to the hg19 or mm10 genome using bowtie2 (v2.5.0)[102]. Low-quality reads were filtered using Samtools (v.1.9) with a MAPQ cut-off score of 30[103]. Only unique, deduplicated reads were retained for further processing. Bigwig files were produced using the deepTools package (v.3.5.1), using an ENCODE hg19 or mm10 v2 blacklist file to discard regions with consistently non-specific signal, and scaled using *E. coli* spike-in controls to normalize sequencing depth. To determine normalization factors based on *E. coli* reads, each sample was aligned to the *E. coli* genome (MG1655), and the unique deduplicated reads were compared across groups per antibody per experiment. The sample with the lowest number of *E. coli* reads was determined ("minimum"), and all samples were scaled by dividing their corresponding *E. coli* read count by this minimum number[104]. For each group, bigwig files were merged and peak callling was conducted using MACS2 (v2.1.0) with the corresponding merged IgG file as control, filtered for peaks with FDR < 0.05[105]. Peak annotation was conducted using HOMER (v4.1.1)[106]. Heatmaps were made either using the DiffBind (v3.8.4) or deepTools (v3.5.5) packages[53,107]. For deepTools, heatmaps were made by merging DEGs from RNA-seq data with TSSs downloaded from the UCSC genome browser using the canonically annotated transcript for each gene. Profiles were generated and statistically analyzed using the deepStats package[108] by using the dsCompareCurves function to perform Wilcoxon Rank-sum tests per-bin. For DiffBind analysis, heatmaps were made for peaks identified by DiffBind's differential peak algorithm, where differential peaks were first filtered using a $\log_2$(fold change) threshold > 0.1 and defined

bioRxiv preprint doi: https://doi.org/10.1101/2025.02.20.639313; this version posted June 2, 2025. The copyright holder for this preprint (which was not certified by peer review) is the author/funder, who has granted bioRxiv a license to display the preprint in perpetuity. It is made available under aCC-BY 4.0 International license.

at $p < 0.05$, where $\log_2$(fold change) was calculated as log2(parity) − log2(NP), based on prior empirical observations used to define thresholds for differential peaks[49]. ChEA analysis on annotated loci was conducted using EnrichR with a significance threshold of adjusted $p < 0.05$[109].

DATA AND MATERIALS AVAILABILITY

The genomics data generated in this study have been deposited in the National Center for Biotechnology Information Gene Expression Omnibus (GEO) database. We declare that the data supporting findings for this study are available within the article and Supplementary Information. Related data are available from the corresponding author upon reasonable request. No restrictions on data availability apply.

bioRxiv preprint doi: https://doi.org/10.1101/2025.02.20.639313; this version posted June 2, 2025. The copyright holder for this preprint (which was not certified by peer review) is the author/funder, who has granted bioRxiv a license to display the preprint in perpetuity. It is made available under aCC-BY 4.0 International license.

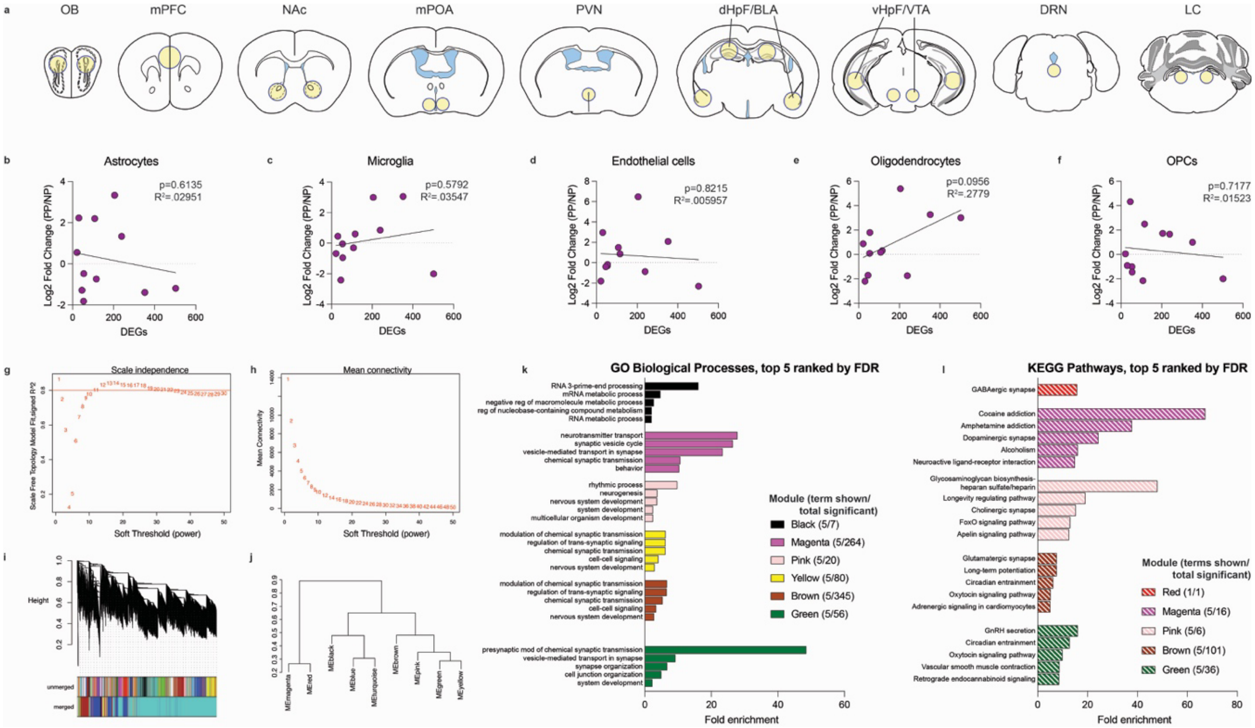

Extended Data 1: Brain-wide transcriptomic analysis of cell markers and gene expression networks. a) Brain regions selected for bulk transcriptional profiling: olfactory bulb (OB), medial prefrontal cortex (mPFC), nucleus accumbens (NAc), medial preoptic area (mPOA), paraventricular nucleus (PVN), dorsal hippocampal formation (dHF), basolateral amygdala (BLA), ventral hippocampus (vHF), ventral tegmental area (VTA), dorsal raphe nucleus (DRN), and locus coeruleus (LC). Yellow circles indicate area selected for tissue micropunching. **B-F)** Nonsignificant correlations between the fold change (NP *vs.* PP) in normalized surrogate proportion variables generated from cell-type deconvolution of bulk RNA-seq data for **B)** astrocytic, **c)** microglia, **d)** endothelial, **e)** mature oligodendrocyte, and **f)** oligodendrocyte precursor cell markers with the number of DEGs identified from each brain region. **g)** Scale independence plot for soft-threshold power selection for WGCNA analysis, illustrating the relationship between β and the scale-free topology fit index, identifying the optimal β value = 12. **h)** Mean connectivity plot illustrating the average network density for each evaluated soft-thresholding power. **i** Dendrogram of gene clustering based on topological overlap, highlighting the hierarchical clustering process used for module detection and merging of similar modules. **j)** Dendrogram of eigengene network adjacency depicting similarity of modules. **k, l)** Significantly enriched **k)** GO Biological Processes and **l)** KEGG pathways, sorted by FDR values, from module gene sets. Bars are color-coded to indicate modules. The total number of significant terms for each analysis is provided.

bioRxiv preprint doi: https://doi.org/10.1101/2025.02.20.639313; this version posted June 2, 2025. The copyright holder for this preprint (which was not certified by peer review) is the author/funder, who has granted bioRxiv a license to display the preprint in perpetuity. It is made available under aCC-BY 4.0 International license.

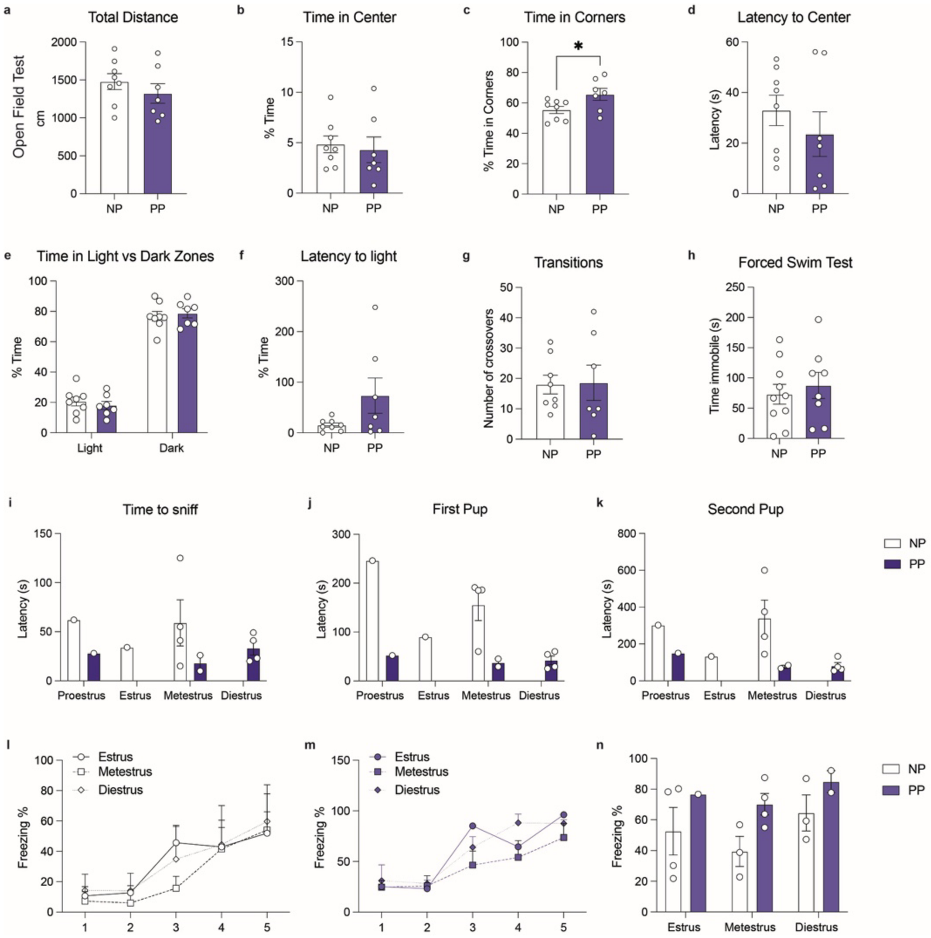

Extended Data 2: Parity associated behavioral adaptations are not influenced by locomotion, anxiety-/depressive-like behaviors, or estrous stage. a-d) In the open field test, there was no change in total distance, time spent in the center, or latency to the center. While there was a significant increase in time spent in corners for PP dams (Student's t-test, $t(13) = 2.307$, $^*p = 0.0382$), this did not impact any other outcomes on the open field test. **e-g)** In the light-dark box, there was no difference in time spent in lights *vs.* dark zones, latency to enter the light zone, or transitions between zones. **h)** There was no difference in time spent immobile on the forced swim test. **i-k)** Stratification of pup retrieval behaviors by estrous stage on the day of testing did not reveal a significant effect of estrous stage. **l-n)** Similarly, stratification of contextual fear conditioning outcomes did not identify a significant effect of estrous stage on acquisition or context recall in NP or PP groups. Error bars represent mean ± SEM. N=6-11 animals/group.

bioRxiv preprint doi: https://doi.org/10.1101/2025.02.20.639313; this version posted June 2, 2025. The copyright holder for this preprint (which was not certified by peer review) is the author/funder, who has granted bioRxiv a license to display the preprint in perpetuity. It is made available under aCC-BY 4.0 International license.

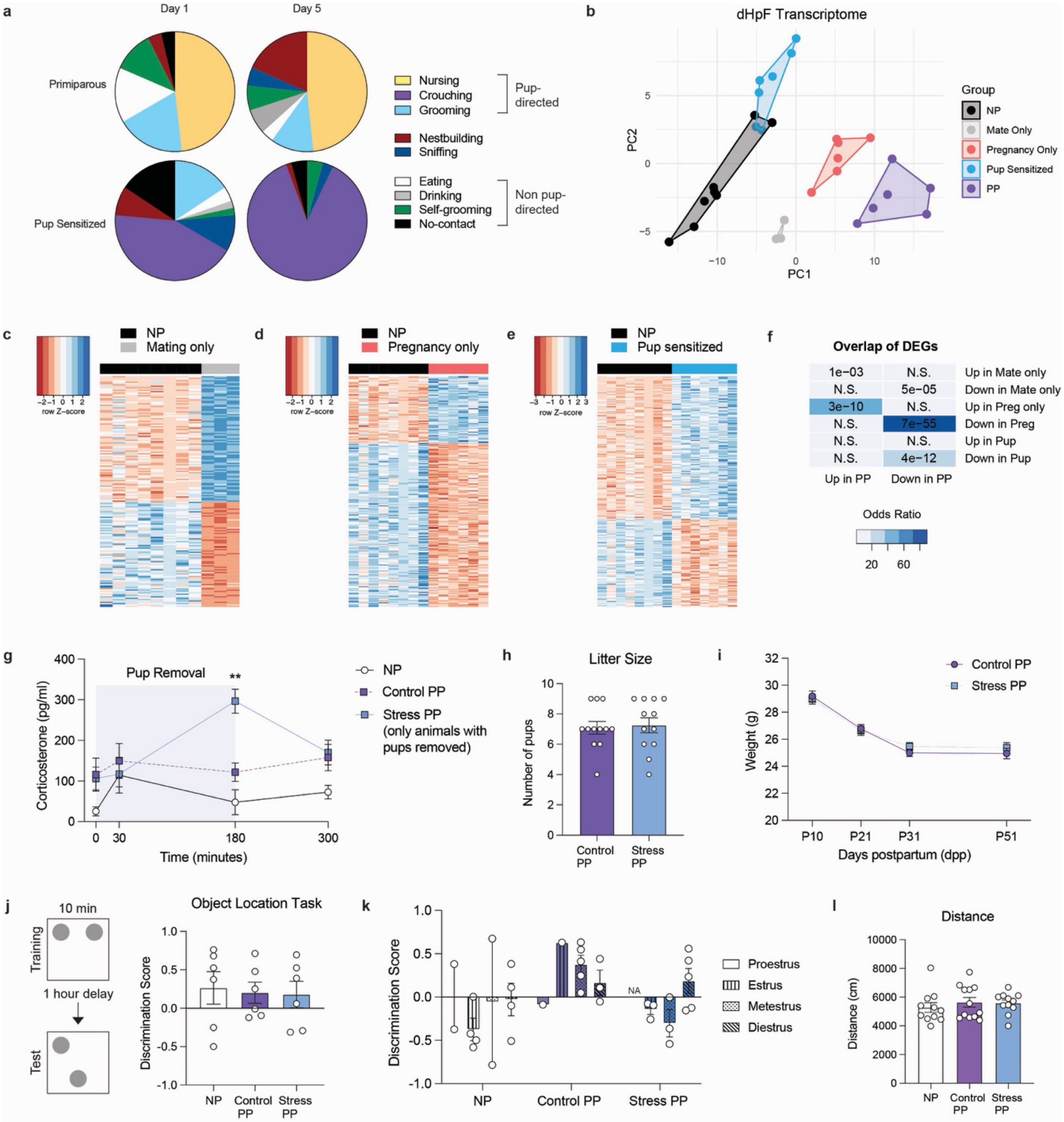

Extended Data 3: Postpartum experiences modulate the extent of parity-associated dHF transcriptomic and behavioral adaptations. a) Pie charts showing significant shifts towards pup-directed behaviors, representing maternal behavior initiation after four days of pup exposure in Pup Sensitized virgin females ($\chi^2(7) = 33.52$, $p = 2.11\times10^{-5}$). **b)** Principle components analysis of NP *vs.* PP DEGs (adj $p < 0.05$) from dHF, showing the Pregnancy Only group clusters most closely with PP. **c)** Heatmaps of all significant DEGs comparing NP *vs.* Mating only, **d)** NP *vs.* Pregnancy only, **e)** NP *vs.* Pup sensitized dHF transcriptomes. **f)** Odds ratio analysis of DEG overlap for all comparisons (*vs.* NP). Insert numbers indicate respective *p* values for each association (N.S., $p > 0.05$). **g)** Pup separation increases maternal corticosterone levels over 3-hours (RM two-way ANOVA, group: $F(2,15) = 12.86$, $p = 0.0006$, time: $F(2.125, 31.87) = 2.759$,

bioRxiv preprint doi: https://doi.org/10.1101/2025.02.20.639313; this version posted June 2, 2025. The copyright holder for this preprint (which was not certified by peer review) is the author/funder, who has granted bioRxiv a license to display the preprint in perpetuity. It is made available under aCC-BY 4.0 International license.

p = 0.0756, interaction: $F(6,45) = 3.294$, $p = 0.009$; Tukey's multiple comparisons test: Control *vs.* Stress PP, $**p < 0.01$). N = 6 animals/group. **h)** No effect of postpartum stress on litter size. N = 12/group. **i)** Postpartum stress did not alter maternal weights. **j)** All groups discriminated the novel location following 10-minutes of training on the object location task (one way ANOVA, $F(2,15) = 0.06452$, $p = 0.9378$) N=6 animals/group. **k)** Stratification of object location test scores did not show a significant effect of estrous stage. **l)** There was no difference in the total distance travelled in the open field test across groups. Error bars represent mean ± SEM.

bioRxiv preprint doi: https://doi.org/10.1101/2025.02.20.639313; this version posted June 2, 2025. The copyright holder for this preprint (which was not certified by peer review) is the author/funder, who has granted bioRxiv a license to display the preprint in perpetuity. It is made available under aCC-BY 4.0 International license.

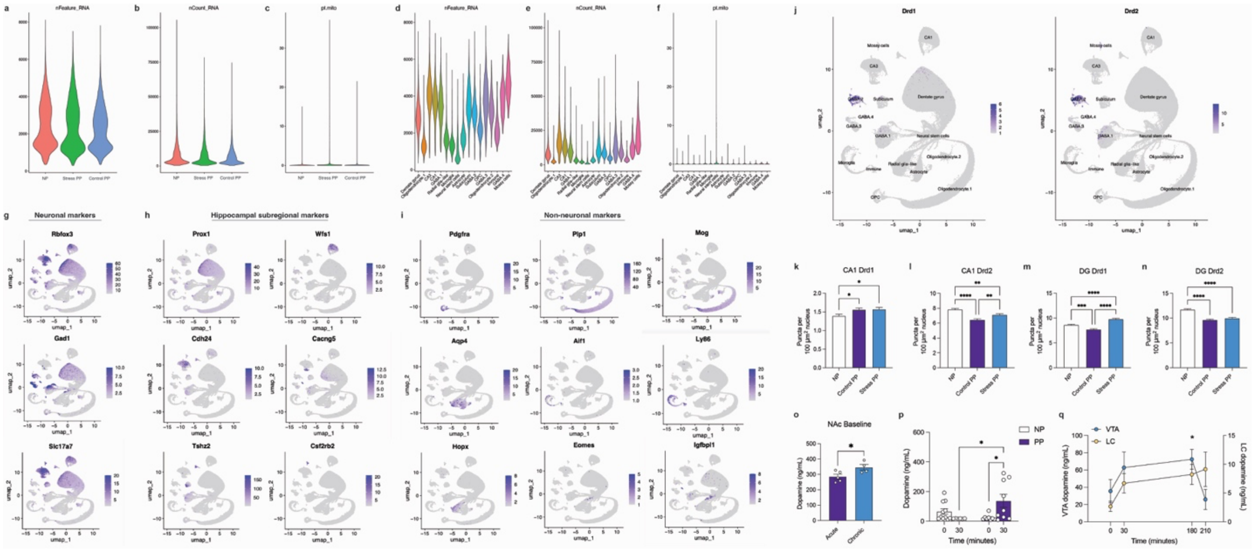

Extended Data 4: snRNA-seq quality control assessments, cell marker gene expression, and postpartum dopamine modulation. Violin plots showing the **(a)** number of detected genes, **(b)** UMI number, and **(c)** mitochondrial gene % by group following regression for mitochondrial gene mapping. **(d-f)** Breakdown of these metrics by annotated clusters show <10% mitochondrial gene across all cell-types, except for a minority of neural stem cells. Unsupervised clustering of snRNA-seq data with cells colored for **(g)** neuronal, excitatory, and inhibitory markers; **(h)** hippocampal subregional markers; and **(i)** non-neuronal markers including for glial and neural progenitor populations. **j)** Unsupervised clustering of snRNA-seq data with cells colored for *Drd1* (left) and *Drd2* (right) expression. **k)** Quantification of *Drd1* mRNA puncta in CA1 nuclei (one-way ANOVA, (F(2,19204) = 3.963, p = 0.019). **l)** Quantification of *Drd2* mRNA puncta in CA1 nuclei (one-way ANOVA, (F(2,19204) = 23.16, p < 0.0001). **m)** Quantification of *Drd1* mRNA puncta in dentate gyrus nuclei (one-way ANOVA, (F(2,21864) = 42.09, p < 0.0001). **n)** Quantification of *Drd2* mRNA puncta in dentate gyrus nuclei (one-way ANOVA, (2,21864) = 41.79, p < 0.0001). Tukey's multiple comparisons test, *p < 0.05, **p < 0.01, ***p < 0.001, ****p < 0.0001. **o)** Chronic postpartum stress resulted in higher baseline levels of dopamine in the nucleus accumbens (NAc; Student's t-test, t(7) = 2.568, *p = 0.0371). N= 4-5 animals/group. **p)** Dopamine response is not induced in NP dHF 30-minutes following littermate separation (two-way ANOVA, interaction of time x group (F(1,30) = 8.78, p = 0.0059, time (F(1,30) = 1.47, p = 0.2359, group, (F(1,30) = 2.054, p = 0.1621); Tukey's multiple comparisons test, *p < 0.05). N = 6-11 animals/group. **q)** Dopamine levels increased during pup separation in the VTA (left y-axis), but not in the locus coeruleus (right y-axis), resembling patterns of NAc and dHF dopamine dynamics (two-way ANOVA, effect of brain region (F(1,46) = 32.32, p < 0.0001), time (F(3,46) = 2.311, p = 0.0866), interaction (F(3,46) = 1.118, p = 0.1109); Dunnett's multiple comparisons test, *p < 0.05). N = 5-8 samples/group. Error bars represent mean ± SEM.

bioRxiv preprint doi: https://doi.org/10.1101/2025.02.20.639313; this version posted June 2, 2025. The copyright holder for this preprint (which was not certified by peer review) is the author/funder, who has granted bioRxiv a license to display the preprint in perpetuity. It is made available under aCC-BY 4.0 International license.

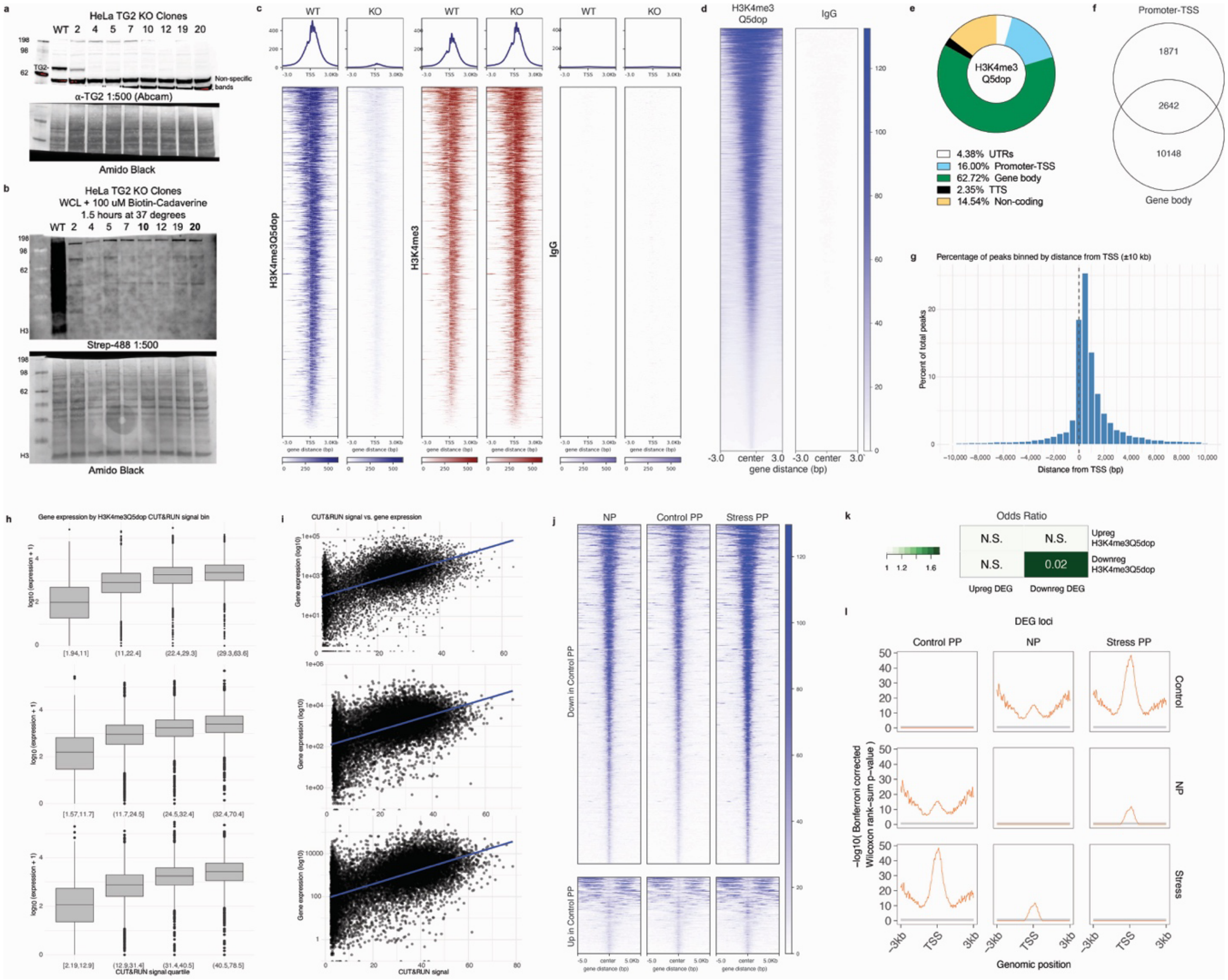

Extended Data 5: H3K4me3Q5dop enrichment corresponds with gene expression. a,b) Validation of TG2 knockout (KO) cell lines using **(a)** Western blot analysis to confirm the absence of TG2 protein, and **(b)** a transamidation activity assay to show loss of biotin-cadaverine incorporation in TG2 KO cells, indicating loss of enzymatic activity. **c)** TG2 KO results in a selective >90% reduction of H3K4me3Q5dop enrichment (*vs.* H3K4me3 alone) at transcription start sites (TSS) compared to WT controls. **d)** Heatmaps of H3K4me3Q5dop and IgG peak enrichment at all enriched loci in NP dHF tissue. **e)** Distribution of H3K4me3Q5dop enrichment occurs mainly at genic loci (83%), with **f)** overlap occurring between uniquely annotated promoter-TSS and gene body (exon/intron) regions and **g)** ~63.4% of total signal occurring within 2kB of the TSS. **h)** Boxplots displaying $\log_{10}$-transformed gene expression across quartiles of H3K4me3Q5dop signal for NP (top), Control PP (middle), and Stress PP (bottom). Significant differences were observed across bins (Kruskal–Wallis $p < 2.2 \times 10^{-16}$) for each group between each quartile. Boxes represent the interquartile range with the median indicated, and whiskers show the rangs with outliers plotted as individual points. **i)** Scatter plots showing the correlation between H3K4me3Q5dop CUT&RUN signal (maximum per gene) and mean gene expression ($\log_{10}$-transformed) for NP (top), Control PP (middle), and Stress PP (bottom). Significant positive correlations were observed for each group (NP: Spearman's $\rho = 0.55$, $p < 2.2 \times 10^{-16}$; Control PP: Spearman's $\rho = 0.52$, $p < 2.2 \times 10^{-16}$; Stress PP: Spearman's $\rho = 0.56$, $p < 2.2 \times 10^{-16}$), indicating

bioRxiv preprint doi: https://doi.org/10.1101/2025.02.20.639313; this version posted June 2, 2025. The copyright holder for this preprint (which was not certified by peer review) is the author/funder, who has granted bioRxiv a license to display the preprint in perpetuity. It is made available under aCC-BY 4.0 International license.

that increased H3K4me3Q5dop enrichment corresponds with higher transcriptional output. **j)** Heatmaps of all differential peaks ($p < 0.05$; log_2FoldChange ≥ |0.1|) between NP/Stress PP vs., separated by directionality and centered on genomic regions to show the majority of altered peaks decrease in Control PP dHF. **k)** Odds ratio analysis of differential H3K4me3Q5dop peaks and differentially expressed genes show significant association between downregulated histone dopaminylation and gene expression changes. Insert numbers indicate respective p values for each association (N.S., $p > 0.05$). **l)** Statistical comparison of H3K4me3Q5dop profiles across groups at loci associated with DEGs. Each panel shows the $-log_{10}$ Bonferroni-corrected *p*-values from bin-wise Wilcoxon rank-sum tests comparing CUT&RUN signal across ±3 kb surrounding TSSs of DEG-associated loci. Comparisons are shown for each pairwise group contrast. Gray bars indicate bins that did not reach statistical significance after multiple hypothesis correction. The orange line reflects the statistical significance of signal differences across the TSS window, with higher values indicating greater confidence in differential enrichment between groups.

bioRxiv preprint doi: https://doi.org/10.1101/2025.02.20.639313; this version posted June 2, 2025. The copyright holder for this preprint (which was not certified by peer review) is the author/funder, who has granted bioRxiv a license to display the preprint in perpetuity. It is made available under aCC-BY 4.0 International license.

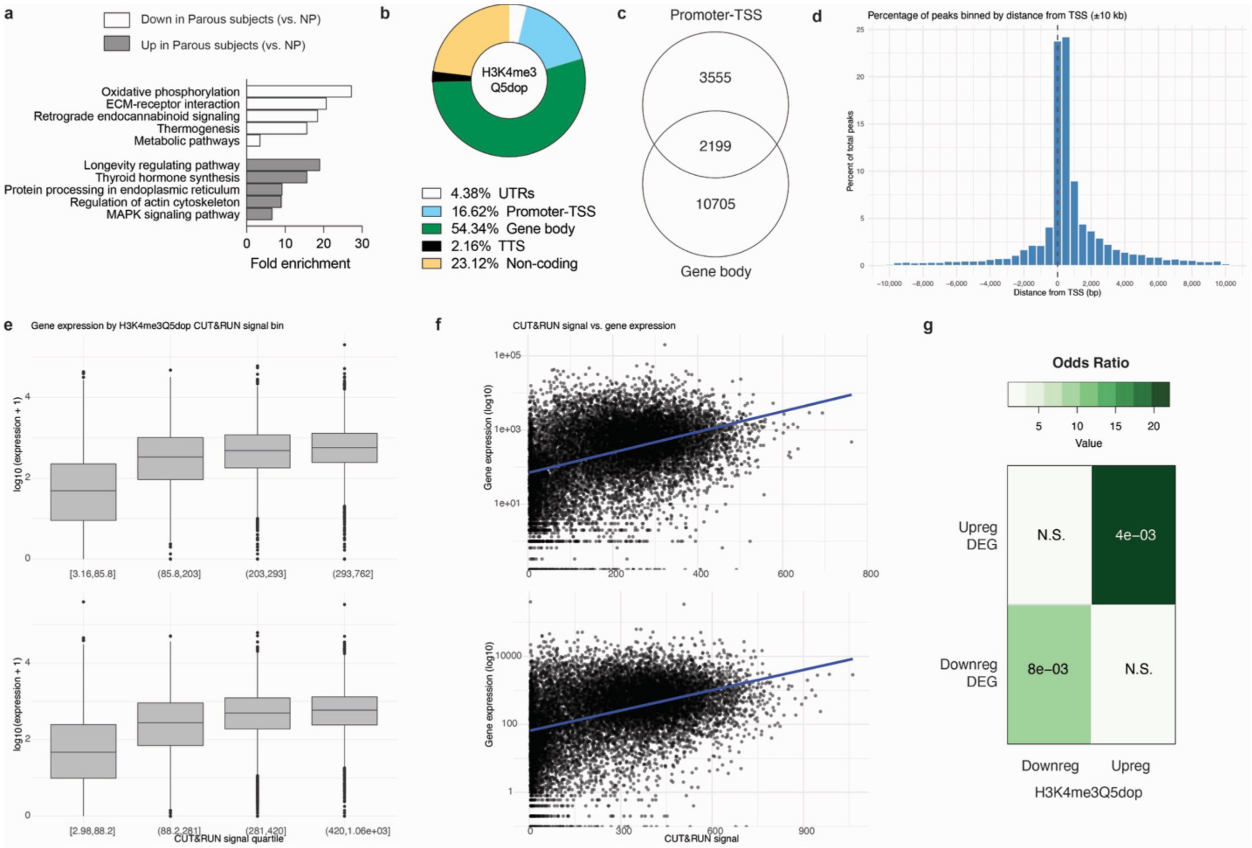

Extended Data 6: H3K4me3Q5dop enrichment in human brain. a) Pathway analysis for DEGs of NP vs. parous human dSub tissues by directionality (padj < 0.05). **b)** Distribution of H3K4me3Q5dop enrichment occurs mainly at genic loci (75.3%), with **c)** overlap occurring between uniquely annotated promoter-TSS and gene body (exon/intron) regions, similar to in mouse dHF, and **d)** ~52.0% of total signal occurring within 2kB of the TSS. **e)** Boxplots displaying $\log_{10}$-transformed gene expression across quartiles of H3K4me3Q5dop signal for NP (top) and parous subjects (bottom). Significant differences were observed across bins (Kruskal–Wallis $p < 2.2 \times 10^{-16}$) for each group and between each quartile. Boxes represent the interquartile range with the median indicated, and whiskers show the rangs with outliers plotted as individual points. **f)** Scatter plots showing the correlation between H3K4me3Q5dop CUT&RUN signal (maximum per gene) and mean gene expression ($\log_{10}$-transformed) for NP (top) and parous subjects (bottom). Significant positive correlations were observed for each group (NP: Spearman's $\rho = 0.41$, $p < 2.2 \times 10^{-16}$; Parous: Spearman's $\rho = 0.40$, $p < 2.2 \times 10^{-16}$), indicating that increased H3K4me3Q5dop enrichment corresponds with higher transcriptional output. **g)** Odds ratio analysis of differential H3K4me3Q5dop peaks and differentially expressed genes show significant association between changes in H3K4me3Q5dop signal and gene expression changes. Insert numbers indicate respective p values for each association (N.S., p > 0.05).

bioRxiv preprint doi: https://doi.org/10.1101/2025.02.20.639313; this version posted June 2, 2025. The copyright holder for this preprint (which was not certified by peer review) is the author/funder, who has granted bioRxiv a license to display the preprint in perpetuity. It is made available under aCC-BY 4.0 International license.

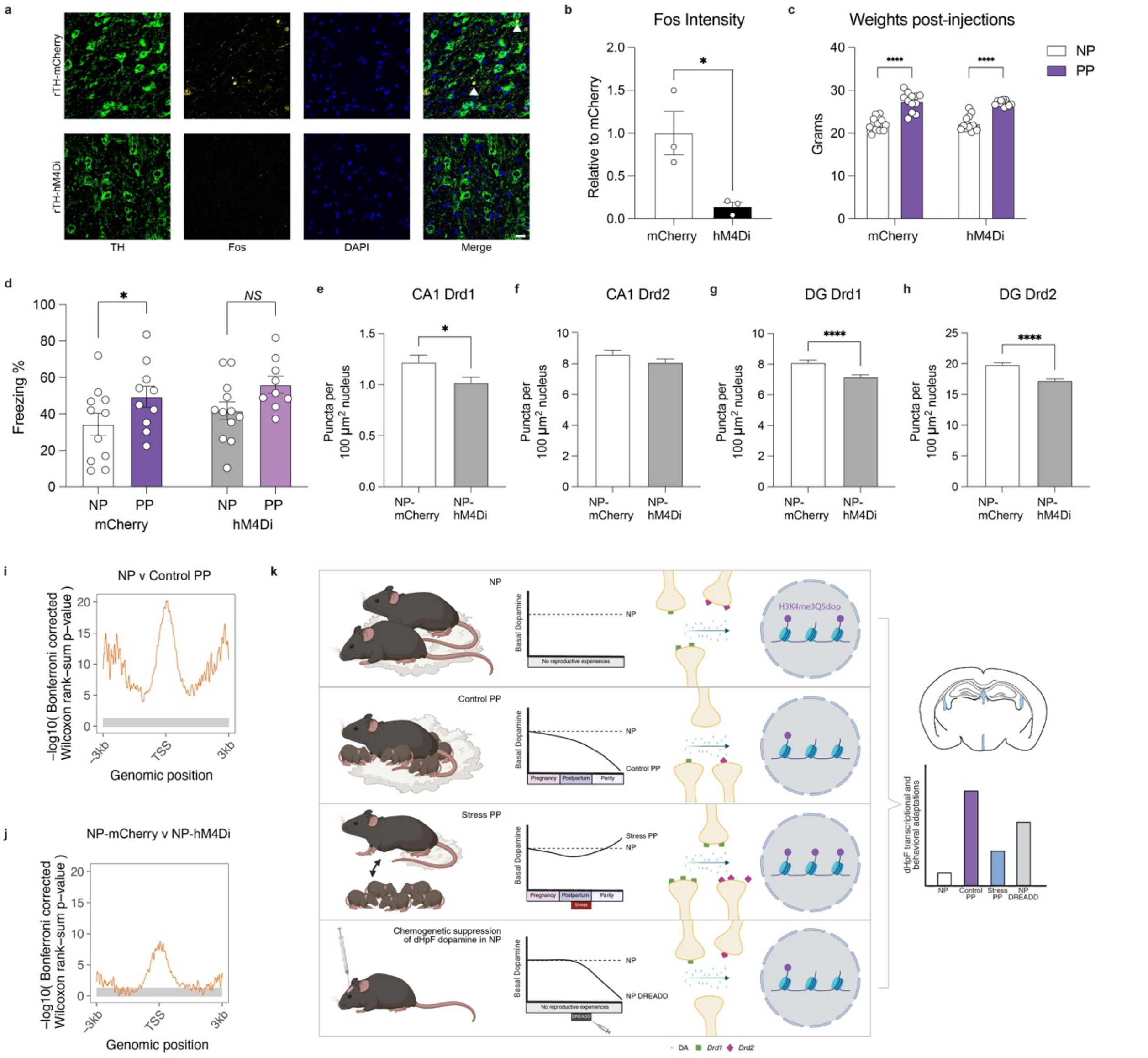

Extended Data 7: Inhibition of VTA-dHF projection mimics sustained changes in dopamine receptor dynamics. a) Representative images of c-Fos immunostaining in the VTA. Scale bars, 20 µm. **b)** Quantification of c-Fos immunoreactivity in VTA TH+ neurons (Student's t-test; $t(4) = 3.299$, $*p = 0.03$). N = 3 animals/group. **c)** Chronic DCZ injections did not alter weights at 21 dpp (two-way ANOVA; main effect of group ($F(1,48) = 128$, $p < 0.0001$), virus ($F(1,48) = 0.0862$, $p = 0.7702$); Tukey's multiple comparison's test, $****p < 0.0001$). **d)** Parity induced significantly greater freezing on the context recall text in mCherry dams, with trending effects between hM4Di groups (two-way ANOVA (effect of group, ($F(1,38) = 7.177$, $p = 0.0109$, virus ($F(1,38) = 1.631$, $p = 0.2093$, Fisher's LSD, *p £ 0.05). N = 9-12/group. **e)** Quantification of *Drd1* mRNA puncta in CA1 nuclei (Student's t-test, ($t(4854) = 2.249$, $*p = 0.00246$). **f)** Quantification of *Drd2* mRNA puncta in CA1 nuclei (Student's t-test, ($t(4854) = 1.502$, $p = 0.1132$). **g)** Quantification of *Drd1* mRNA puncta in dentate gyrus nuclei (Student's t-test, ($t(11946) = 3.900$, $****p < 0.0001$). **h)** Quantification of *Drd2* mRNA puncta in dentate gyrus nuclei (Student's t-test, ($t(11946) = 6.034$, $****p < 0.0001$). **i, j)** Statistical comparison of H3K4me3Q5dop profiles between NP-mCherry

bioRxiv preprint doi: https://doi.org/10.1101/2025.02.20.639313; this version posted June 2, 2025. The copyright holder for this preprint (which was not certified by peer review) is the author/funder, who has granted bioRxiv a license to display the preprint in perpetuity. It is made available under aCC-BY 4.0 International license.

and NP-hM4Di at loci associated with **(i)** NP *vs.* Control PP DEGs and **(j)** NP-mCherry *vs.* NP-hM4Di DEGs. Each panel shows the $-\log_{10}$ Bonferroni-corrected *p*-values from bin-wise Wilcoxon rank-sum tests comparing CUT&RUN signal across ±3 kb surrounding TSSs of DEG-associated loci. Comparisons are shown for each pairwise group contrast. Gray bars indicate bins that did not reach statistical significance after multiple hypothesis correction. The orange line reflects the statistical significance of signal differences across the TSS window, with higher values indicating greater confidence in differential enrichment between groups. **k)** Schematic of working model.
