# Supplementary material for: Persistent dopamine-dependent remodeling of the neural transcriptome in response to pregnancy and postpartum
Source: bioRxiv. 2025 Feb 25:2025.02.20.639313. Preprint. [Version 1] doi: 10.1101/2025.02.20.639313 (PMC11888212; doi:10.1101/2025.02.20.639313)
Supplement: Supplementary file 1 [file NIHPP2025.02.20.639313v1-supplement-2.pdf]

bioRxiv preprint doi: https://doi.org/10.1101/2025.02.20.639313; this version posted February 25, 2025. The copyright holder for this preprint (which was not certified by peer review) is the author/funder, who has granted bioRxiv a license to display the preprint in perpetuity. It is made available under aCC-BY 4.0 International license.

Supplementary Materials for

Persistent dopamine-dependent remodeling of the neural transcriptome in response to pregnancy and postpartum

Jennifer C Chan *et al.*

Corresponding author: Ian Maze, ian.maze@mssm.edu

MATERIALS AND METHODS

Animals

Wild-type C57BL6/J mice were purchased from Jackson Laboratories at 8-weeks old, and maintained on a 12-h/12-h light/dark cycle throughout the entirety of the experiments. Mice were provided with *ad libitum* access to water and food throughout the entirety of the experiments. All behavioral testing occurred during the animals' light cycle. Experimenters were blind to experimental group, and the order of testing was counterbalanced during behavioral experiments. All animal procedures were performed in accordance with NIH guidelines and with the approval of the Institutional Animal Care and Use Committee of the Icahn School of Medicine at Mount Sinai.

Breeding

Adult virgin female mice were pair bred in-house with age-matched males. Males were removed after a maximum of 5 days, and pregnant females were singly-housed at least 2 days before parturition. Pups were counted on the day of birth (0 dpp) and weaned at 21 dpp. Only dams with litters between 4-10 pups were used for all experiments. On the day of weaning, dams were group-housed into cages of 3-5 mice with animals of the same experimental condition. For timed breedings, copulation plugs were checked every morning within 1-hour after lights on, where confirmation of a plug was designated as E0.5, signaling the immediate removal of the female to

bioRxiv preprint doi: https://doi.org/10.1101/2025.02.20.639313; this version posted February 25, 2025. The copyright holder for this preprint (which was not certified by peer review) is the author/funder, who has granted bioRxiv a license to display the preprint in perpetuity. It is made available under aCC-BY 4.0 International license.

her own cage with a nestlet. Virgin NP females were age-matched for each experimental cohort. Mating-experienced NP females were confirmed for the presence of a copulation plug. For Pregnancy Only dams, pups were removed at 0 dpp within 3 hours after lights-on to minimize maternal-offspring interactions. For comparison of PP dams to pup sensitized virgin females, litters were culled to 4 pups to equate litter size with the number of pups used for each sensitized female. In all other experiments, litters were not culled.

Postpartum stress paradigm

Stress PP females were subjected to limited nesting and maternal separation from 10-20 dpp, as previously described[1–3]. On each day of separation, the entire litter was removed to a clean cage with Sani-Chip bedding for 3-4 hours. Separations occurred during the light cycle, and the timing varied each day to minimize predictability and acclimatization. EnviroDri nesting material was depleted to 1/3 of control cages during the days of separation. Following pup weaning on 21 dpp, the nesting material was restored to normal levels, and dams were group-housed into cages of 3-5 with animals of the same experimental condition.

Pup sensitization

Pup sensitization was conducted as previously described[4,5]. On the first day of pup sensitization, virgin NP females were presented with four pups (postnatal day 5) from a cage consisting of a lactating donor dam and her litter. Donor pups were exchanged for satiated pups from the same litter every 8-12 hours for 21 days. Donor pups were weighed daily to ensure continual weight-gain throughout the experiment, and only pups that exhibited consistent weight-gain were used. Behavioral observations were conducted during the first four days of sensitization. Each dam was observed for 30 minutes during the light phase for the following maternal behaviors:

bioRxiv preprint doi: https://doi.org/10.1101/2025.02.20.639313; this version posted February 25, 2025. The copyright holder for this preprint (which was not certified by peer review) is the author/funder, who has granted bioRxiv a license to display the preprint in perpetuity. It is made available under aCC-BY 4.0 International license.

licking/grooming, crouching, and nestbuilding. During these observation periods, females were closely monitored to ensure that they did not display aggressive behavior toward the donor pups. On the last day of sensitization, donor pups were weaned from the cage, and sensitized females were group-housed into cages of 3-5 mice with animals of the same experimental condition.

Brain tissue collections

Animals were sacrificed by rapid decapitation. Whole brains were flash frozen with cold 2-methylbutane and stored at −80°C until further processing. Flash-frozen brains were sectioned at −20°C using a 1 mm mouse coronal brain matrix (Stoelting Co.). Tissues enriched for the brain region of interest were micropunched using a hollow needle (Ted Pella) according to the Allen Brain Atlas (see **Fig. S1A**).

Behavioral analyses

All animals (18-30 weeks, depending on the time from pup weaning) were handled for 2 minutes for two consecutive days prior to initial behavioral testing. Animals were habituated to the testing room for 1 hour prior to each behavioral assay. All testing occurred during the light phase.

Pup retrieval: All animals were individually housed for 24-hours prior to testing. Following habituation, 2 pups from a donor litter with a lactating dam (aged 4-6 dpp) were placed in different corners opposite to the nest in the home cage. Pup-directed interactions were recorded from above for 15-minutes or until both pups were successfully retrieved into the nest. Retrieval latency was calculated as [time pup was first placed into the nest by animal - time first pup was placed into the cage by experimenter].

bioRxiv preprint doi: https://doi.org/10.1101/2025.02.20.639313; this version posted February 25, 2025. The copyright holder for this preprint (which was not certified by peer review) is the author/funder, who has granted bioRxiv a license to display the preprint in perpetuity. It is made available under aCC-BY 4.0 International license.

Contextual fear conditioning: On day 1, mice were habituated for 10 minutes to the testing chamber, which consisted of a square plexiglass box with a metal grid inside a sound-attenuating cabinet wiped with 70% ethanol (Med Associates, VT). On day 2, following a 3-minute baseline measurement, mice were trained with five 2.0-second 0.7 mA foot shocks delivered with an intertrial interval of 90-seconds. Testing for conditioned fear responses (freezing) for a total of 5-minutes occurred 24-hours following training. Freezing was measured using ANY-maze software connected to a camera positioned above the testing chamber. Freezing is expressed as a percentage of the total test time or as a percentage of the 60-seconds prior to each shock during conditioning. Animals with freezing levels exceeding 40% prior to the first shock were excluded to remove potential confounding effects of heightened baseline responsivity that could interfere with accurately assessing conditioned fear.

Open Field: Mice were placed in a 16x16 square arena under dim lighting for 5 minutes. A camera positioned overhead recorded the total distance and time spent in the center *vs.* periphery using Ethovision software.

Object location task: For training, animals were allowed to freely explore two identical objects placed equidistant from adjacent corners of a 16x16 cm square arena for 5 or 10 minutes, before being returned to the home cage. Following a 1-hour delay, the animals were returned to the arena for testing, during which time one object was moved to the opposing corner. During the test, the animals were allowed to freely explore the objects for 5-minutes. A camera positioned overhead recorded time spent exploring each object using Ethovision software. The discrimination score was calculated as [(time spent with moved object) – (time spent with unmoved object)/[(time spent with moved object) + (time spent with unmoved object)].

bioRxiv preprint doi: https://doi.org/10.1101/2025.02.20.639313; this version posted February 25, 2025. The copyright holder for this preprint (which was not certified by peer review) is the author/funder, who has granted bioRxiv a license to display the preprint in perpetuity. It is made available under aCC-BY 4.0 International license.

Light-dark box: Anxiety-like behavior was tested in an apparatus containing two interconnected 20x20 cm compartments (Omnitech Electronics Inc.). One compartment was illuminated during the session ("light" side), while the other was covered by an opaque black perspex lid ("dark" side). The distance, time spent, and number of crossovers in each compartment were automatically recorded by Fusion software during the 10-minute test.

Forced Swim Test: Mice were placed in a 4 liter glass beaker with 2 liters of room temperature water for 8 minutes. Each session was recorded and scored by a blinded observer. The total number of seconds that mice were immobile during the last 5 minutes of the test were recorded, as previously described[6].

Observation of pup-directed behaviors in the home cage: Confirmation of maternal behavior in pup sensitized females was conducted based on prior studies[7]. Animals were observed from the first to fourth day of pup sensitization, based on published work that maternal sensitization in C57BL6/J females occurs following 4-days of pup exposures[8,9]. Observations occurred in the light period in the first 30-minutes after pups were placed in the cage. Each animal was scored every 3-minutes, with the observed behavior recorded as one or more of the following categories: nestbuilding, grooming, sniffing, crouching, nursing, eating, drinking, self-grooming, no-contact. PP dams were scored for the first 4-days following birth (0-3 dpp) concurrently for comparison.

Estrous cycle testing

Vaginal samples were taken on each day of behavioral testing. 15 µL of sterile PBS was gently pipetted into the vagina and mounted on a glass slide. Vaginal smears were stained with crystal violet dye, washed twice with water, and cover slipped with glycerol. Three 20x images per sample

bioRxiv preprint doi: https://doi.org/10.1101/2025.02.20.639313; this version posted February 25, 2025. The copyright holder for this preprint (which was not certified by peer review) is the author/funder, who has granted bioRxiv a license to display the preprint in perpetuity. It is made available under aCC-BY 4.0 International license.

were acquired on a light microscope. Estrous stage was determined using the pretrained network of EstrousNet[10], and confirmed afterwards by a trained experimenter based on cytology of nucleated, cornified, or leukocytic cells.

Viral Transduction

Mice were anesthetized with ketamine (100mg/kg) and xylazine (10 mg/kg) i.p. and positioned in a stereotaxic frame (Kopf instruments). Given the widespread changes in dopamine receptor expression across dHpc subregions, we did not restrict our viral manipulations to a specific subregion. 2 µl of retrograde AAV.rTH.PI.Cre.SV40 (titer $\geq 7\times10^{12}$ vg/mL, Addgene #107788-AAVrg) was infused bilaterally into the dHpc at 0.2 uL/min using the following coordinates: 7° angle; anterior-posterior (AP) -2.2 mm, medial-lateral (ML) ±2.0 mm, dorsal-ventral (DV) -2.0 mm. 1 µl of pAAV-hSyn-DIO-mCherry (titer $\geq 4\times10^{12}$ vg/mL, Addgene #50459-AAV2) or pAAV-hSyn-DIO-hM4D(Gi)-mCherry (titer $\geq 5\times10^{12}$ vg/mL; Addgene #44362-AAV2) was bilaterally infused into the VTA using the following coordinates: 7° angle; AP -3.3 mm, ML ±0.9 mm; DV -4.6 mm. Needles remained in place for 7 minutes following injection to minimize virus diffusion. Viral validations were conducted at least 21 days post-surgery to allow for optimal viral expression and recovery.

Chemogenetic manipulation

To directly manipulate dopamine signaling in dHpc, we selectively targeted the VTA-dHpc projection during a window established as being critical for dopaminergic downregulation. Following 7 days of recovery, surgerized female mice were randomly assigned to NP or PP groups. PP females were pair bred with naïve male mice for a maximum of 5 days, and individually housed prior to parturition. From 10-20 dpp, PP females were injected subcutaneously with

bioRxiv preprint doi: https://doi.org/10.1101/2025.02.20.639313; this version posted February 25, 2025. The copyright holder for this preprint (which was not certified by peer review) is the author/funder, who has granted bioRxiv a license to display the preprint in perpetuity. It is made available under aCC-BY 4.0 International license.

deschloroclozapine (DCZ, Tocris #7193) – to minimize off-target effects[11] - at 1ug/kg in 1% DMSO or vehicle (saline). NP females were injected with DCZ concurrently. Following weaning on 21 dpp, PP dams were group-housed with animals of the same experimental condition. Behavioral testing occurred beginning at 28-days post-weaning (49 dpp), and brain tissues were collected following contextual fear conditioning for further analyses.

RNAscope in situ hybridization and analysis

Fresh frozen brains were cut into 12-16 μm thick slices in the coronal plane with a cryostat (Leica CM3050-S), mounted on charged Superfrost Plus microscope slides (Thermofisher #P36934), and stored at -80°C until processing. Sections were post-fixed with 4% PFA for 1.5 hours at 4°C, and permeabolized with hydrogen peroxide (10 minutes RT) and Protease III (30 minutes RT). The RNAscope Multiplex Fluorescent Reagent Kit v2 (ACD Bio) was used according to manufacturer’s instructions to sequentially stain sections with the following probes: Drd1a (Mm-Drd1a-C1, #461901) and Drd2 (Mm-Drd2-C3, #406501-C3). RNAscope probes were visualized using TSA Vivid Fluorophores (Tocris) at 1:750 dilution. Sections were counterstained with DAPI (ACD Bio) and mounted using ProLong Gold Antifade Mountant (Thermo). Confocal images (3 images per animal, 1024 × 1024 pixels) were acquired on a Zeiss LSM 780 upright microscope using a 40X objective with Zen Black software, with 5x1 tiled images. Images were averaged across 8 consecutive acquisitions at a bit depth of 16 bits, with 2 z-stacks acquired per image. Subcellular quantification of individual puncta per 100 μm nucleus - identified by cellular detection of DAPI staining - was performed for each maximum intensity projected image using QuPath software (v.0.5.1)[12]. Regions of interest (CA1 and dentate gyrus) were annotated and determined by superimposing images onto the Allen Brain Atlas.

bioRxiv preprint doi: https://doi.org/10.1101/2025.02.20.639313; this version posted February 25, 2025. The copyright holder for this preprint (which was not certified by peer review) is the author/funder, who has granted bioRxiv a license to display the preprint in perpetuity. It is made available under aCC-BY 4.0 International license.

Immunofluorescence and analysis

Mice were anesthetized with isoflurane and perfused with cold 1×PBS followed by 4% PFA. Brains were post-fixed in 4% PFA overnight and then transferred to a 30% sucrose/PBS solution for 2 days. Brains were sectioned at 40 µm thickness using a Leica CM3050-S cryostat, with serial sections collected from the VTA. For each subject, 2–3 brain slices were blocked for 2 hours (0.1% Triton X-100, 10% normal donkey serum), followed by overnight incubation at 4 °C with primary antibodies: chicken anti-tyrosine hydroxylase (1:500, Aves Labs #TYH) and rabbit anti-Fos (1:2000, Synaptic Systems #226-008). The next day, slices were incubated for 2 hours at RT with fluorescent-conjugated secondary antibodies (donkey anti-chicken Alexa Fluor 488, Invitrogen #A78948; donkey anti-goat Alexa Fluor 680, Invitrogen #A10043). Slices were counterstained with DAPI (1:10000, Thermo Scientific #62248) and mounted with ProLong Gold Antifade Mountant (Thermo Fisher #P36934). Confocal images (2-3 replicates per animal, 1024 x 1024 pixels) were acquired on a Zeiss LSM 780 upright microscope using a 40x objective with Zen Black software. Images were averaged across 8 consecutive acquisitions at a bit depth of 16 bits. Image analysis was conducted using FIJI software (NIH). The Fos and TH channels were thresholded using the MaxEntropy method to define regions of interest. Following identification of colocalized Fos+/TH+ signal, Fos intensity was measured and averaged from 4-6 images per animal. Brightness and contast were adjusted for representative images.

Dopamine ELISA

Brain tissue dopamine levels were assessed in response to 3 hours of pup separation in the home cage. Independent biological replicates were collected at each time point, with separate animals used for each measurement. Brains were rapidly harvested (see Brain Tissue Collection, above) at

bioRxiv preprint doi: https://doi.org/10.1101/2025.02.20.639313; this version posted February 25, 2025. The copyright holder for this preprint (which was not certified by peer review) is the author/funder, who has granted bioRxiv a license to display the preprint in perpetuity. It is made available under aCC-BY 4.0 International license.

baseline (prior to pup removal, 0 min), during pup removal (30 and 180 min), and 30 min after pups were returned to the home cage (210 min). For assessment of NP females, samples were collected in the home cage 30-min following removal of a littermate. Equal weights of micropunched brain tissues were homogenized in lysis buffer (0.01 N HCl, 1 mM EDTA, 4 mM sodium metabisulfite). Tissue dopamine levels were assessed using the Dopamine (Research) ELISA Kit (ALPCO Diagnostics) according to manufacturer's instruction.

HPA axis assessment

Plasma corticosterone levels were assessed in response to 3-hours of pup separation in the home cage. Testing was initiated within 2-hours after lights on. Tail blood was collected prior to pup removal (0 min), during pup removal (30 and 180 min), and 120 min after pups were returned to the cage (300 min). Samples for control animals in the home cage, used to account for the effects of handling-induced stress, were collected concurrently. Blood samples were immediately mixed with 50 mM EDTA and centrifuged at 5000 rpm for 10 minutes. Plasma was collected and stored at −80 °C until analysis. Corticosterone levels were quantified using a Corticosterone ELISA kit (ENZO Life Sciences) according to manufacturer's instruction.

Statistics

Statistical analyses for behavioral and immunoassay data were conducted using Prism software (GraphPad, v.10.4.1). For experiments involving multiple conditions, one-way or two-way ANOVAs were performed, followed by *post hoc* analyses when appropriate. For time course analyses where multiple measurements were taken from the same animal, repeated measures ANOVAs were performed. Two-tailed Student's t-tests were used for comparisons between two conditions. Behavioral data derived from manual observations and pregnancy/litter outcomes were

bioRxiv preprint doi: https://doi.org/10.1101/2025.02.20.639313; this version posted February 25, 2025. The copyright holder for this preprint (which was not certified by peer review) is the author/funder, who has granted bioRxiv a license to display the preprint in perpetuity. It is made available under aCC-BY 4.0 International license.

analyzed using chi-square tests. Grubb's test (alpha = 0.05) was applied to detect outliers where necessary. Statistical significance was defined as $p \leq 0.05$.

Bulk RNA-seq and analysis

RNA isolation and library preparation: Total mRNA was extracted from frozen brain tissues after homogenization in Trizol Reagent (Thermo Fisher) and cleaned using RNeasy Microcolumns (Qiagen) following the manufacturer's instructions. For RNA-seq library preparation, 150 ng of mRNA per sample was used with either the Illumina Stranded mRNA Prep Kit (Illumina, #20040534) or the TruSeq RNA Library Prep Kit v2 (Illumina, #RS-122-2001), according to the manufacturer's protocols. Library quality was assessed using a Qubit Fluorometer 2.0 (Thermo Fisher) and a High Sensitivity D5000 TapeStation assay (Agilent) before sequencing on a NovaSeq 6000 or NovaSeq X system.

Differential expression analysis: Raw fastq files, containing an average of 20–30 million reads per sample, were processed for pseudoalignment and abundance quantification using Kallisto (v. 0.46.1) against the Ensembl Mus musculus reference (v. 79)[13]. To filter lowly-expressed genes, only those with a total read count of at least 10 across all samples were retained. To account for unwanted variation among samples within each sequencing experiment that could arise from technical or biological factors unrelated to the conditions of interest (including litter size, estrous stage, day of sample collection, etc.), RUVs (v1.32.0) was applied with a negative control gene set derived from the total genes identified per sequencing experiment, after ensuring that unwanted variation did not correlate with covariates of interest, as described previously[14,15]. Differential expression analysis was performed using DESeq2 (v1.38.3)[16], with significant genes defined by an adjusted p-value < 0.05. For brain-wide transcriptome comparisons in which samples were

bioRxiv preprint doi: https://doi.org/10.1101/2025.02.20.639313; this version posted February 25, 2025. The copyright holder for this preprint (which was not certified by peer review) is the author/funder, who has granted bioRxiv a license to display the preprint in perpetuity. It is made available under aCC-BY 4.0 International license.

processed across multiple sequencing runs and stemming from separate cohorts, pairwise comparisons were performed independently for each brain region. For all other experiments, where subjects came from the same cohort and were processed in a single sequencing run, all groups were analyzed together to maintain consistent normalization within the experiment. Gene expression time course analyses examining the periods before, during, and after pregnancy and postpartum were performed on normalized count data using the ImpulseDE2 package (v0.99.10)[17] for each brain region. Significant genes exhibiting transient regulation or monotonous changes in expression were identified using case-only differential expression analysis, with a Q-value threshold of 0.05.

Weighted gene co-expression analysis: To identify brain-wide gene co-expression networks, normalized count data for all brain regions were compiled and analyzed using the WGCNA package (v1.73)[18]. Co-expression networks were constructed from the 7,500 most variable genes, determined by ranking gene variance. A soft threshold power of 12 was identified with the pickSoftThreshold function to ensure scale-free network properties. Modules were identified based on dissimilarity of a signed topological overlap matrix (TOM), and named with an arbitrary color. The "gray" module encompassed genes that did not segregate into any specific module, and was therefore removed from further analyses. To assess the enrichment of differentially expressed genes (DEGs) within each brain region per module, Fisher's exact tests were performed using the fisher.test() function in R (v4.3.0). To analyze the correlation between gene modules and brain regional sensitivity to parity, brain regions were categorized into two groups based on the top five regions with significant DEG overlap. These regions were designated as "High Sensitivity" regions, while all other regions were classified as "Low Sensitivity." Pearson correlation coefficients were calculated to examine the relationship between each module and the trait

bioRxiv preprint doi: https://doi.org/10.1101/2025.02.20.639313; this version posted February 25, 2025. The copyright holder for this preprint (which was not certified by peer review) is the author/funder, who has granted bioRxiv a license to display the preprint in perpetuity. It is made available under aCC-BY 4.0 International license.

(regional sensitivity × parity status), with statistical significance determined by Student's t-tests ($p < 0.05$). Heatmaps were generated to visualize these module-trait correlations, and module-specific gene lists were exported for pathway enrichment analysis.

Pathway and predicted upstream regulator analyses: Functional annotation of DEGs was conducted using ShinyGO (v0.81)[19], with all protein-coding genes in the mm10 genome used as the background. Pathways with an FDR < 0.05 were considered significant. All significant pathways and associated statistics are provided in the Supplementary Tables. For figures, relevant pathways were selected from the top 10 or 1/3 of significant terms, ranked by FDR, to emphasize processes consistent with hypotheses informed by published literature. For GO term selection, Revigo[20] was used to reduce redundancy of overlapping GO terms when needed. Ingenuity Pathway Analysis (IPA; Qiagen, Inc., v.01-23-01) was used to predict upstream regulators for DEG lists[21]. For "High Sensitivity" and "Low Sensitivity" regions (see *WGCNA* section), DEGs shared by at least two or three brain regions were extracted, irrespective of the direction of change. The IPA software was used to identify upstream regulators associated with each DEG list, with statistical significance defined as $p < 0.05$. To investigate the mechanisms underlying parity programming of dHpc plasticity, significant upstream regulators were systematically prioritized based on one or more of the following criteria: (1) multiple molecules involved in the same signaling pathways (e.g., progesterone and its receptor, PGR); (2) structurally similar molecules that engage analogous signaling cascades (e.g., levodopa and dopamine); (3) molecules known to be influenced by reproductive exposures; (4) molecules demonstrated to play a role in hippocampal plasticity; (5) molecules identified as significant in both "High Sensitivity" and "Low Sensitivity" regulator lists; and (6) molecules with high statistical significance values. Following selection, upstream regulators were categorized into general molecular classes (e.g., "Hormone,"

bioRxiv preprint doi: https://doi.org/10.1101/2025.02.20.639313; this version posted February 25, 2025. The copyright holder for this preprint (which was not certified by peer review) is the author/funder, who has granted bioRxiv a license to display the preprint in perpetuity. It is made available under aCC-BY 4.0 International license.

"Transcription Factor," "Lipid") and grouped for visualization. Data are presented as a bubble plot, with marker shape representing number of brain regions sharing DEGs and marker size corresponding to significance.

Gene expression overlap analyses: Jaccard indices were calculated for overlapping gene lists using the GeneOverlap package (v1.36.0)[22], with significance defined by $p < 0.05$. Fisher's exact tests were calculated using the base *fisher.test()* function in R (v. 4.3.0) following construction of contingency tables for each comparison. Transcriptome-wide, threshold-free gene expression overlap was visualized using Rank-Rank Hypergeometric Overlap (RRHO) heatmaps generated with the RRHO2 package (v1.0)[23]. Gene lists were ranked by signed p-values, calculated as the log10-transformed nominal p-value multiplied by the sign of the fold change, without applying differential expression thresholds.

Cell-type deconvolution: Brain cell-type proportion was estimated from normalized expression data using the BRETIGEA package (v.1.0.4)[24] with default markers. For comparisons between groups, surrogate proportion variables were normalized using the Normalize function in Prism (GraphPad, v. 10.4.1). Each sub column was normalized separately, with the smallest value set to 0% and the largest value set to 100%. To calculate $\log_2$(fold change) between groups, normalized values per cell-type per group were averaged and expressed as the log2 ratio of the group means.

Single nuclei RNA-seq

Nuclei isolation and library preparation: For each animal, 2 mm dHpc-enriched tissue micropunches were collected bilaterally from consecutive 1 mm slices from -0.80 to -2.80 mm relative to bregma for a total of 4 punches (Ted Pella). Samples were processed in batches of 4-6, with each group represented within each batch. Nuclei were isolated using a modified version of

bioRxiv preprint doi: https://doi.org/10.1101/2025.02.20.639313; this version posted February 25, 2025. The copyright holder for this preprint (which was not certified by peer review) is the author/funder, who has granted bioRxiv a license to display the preprint in perpetuity. It is made available under aCC-BY 4.0 International license.

a sucrose density gradient isolation protocol[25]. Briefly, thawed tissues were placed in 1 mL of lysis buffer (0.32 M sucrose, 5 mM $CaCl_2$, 3 mM magnesium acetate, 0.1 mM EDTA, 10 mM Tris-HCl pH 8, 1 mM DTT, 0.1% Triton X-100) with 50 µL of 25 U/mL RNase inhibitor (Takara #2313B) in a dounce homogenizer (Wheaton #357538). Homogenization was performed with 20 strokes using a tight pestle. Another 1 mL of lysis buffer was added, followed by an additional 10 strokes. The resulting 2 mL homogenate was transferred to a 15 mL Open-Top Thinwall Polypropylene Tube (Beckman-Coulter #361707). The homogenizer and pestle were rinsed with 2 mL of lysis buffer, and this wash was combined with the homogenate for a total of 4 mL. The homogenate was carefully underlaid with 9 mL of sucrose solution (1.8 M sucrose, 3 mM magnesium acetate, 1 mM DTT, 10 mM Tris-HCl pH 8) and ultracentrifuged at 24,000 rpm for 1 hour at 4 °C using a Sorvall™ WX+ centrifuge. After centrifugation, the supernatant and debris at the interface were gently removed. The nuclear pellet was resuspended in 1 mL of resuspension buffer (0.02% bovine serum albumin in DPBS with 25 µL of 25 U/mL RNase inhibitor) and incubated on ice for 10 minutes. The suspension was passed through a 35 µm nylon mesh filter (Corning #352235) into a 1.5 mL RNase/DNase-free microcentrifuge tube and centrifuged at 2600 x g for 10 minutes at 4 °C. Supernatants were discarded, and nuclei were resuspended in 200 µL of resuspension buffer. A 10 µL aliquot of the nuclei suspension was stained with Trypan Blue to assess quality and concentration using a Countess 3 Automated Cell Counter. Nuclei suspensions were loaded onto a Chromium Single Cell 3′ chip (10X Genomics, v3) and processed according to the manufacturer's protocol, targeting 10,000 nuclei. Single-nuclei libraries were generated using the 10X Chromium Next GEM Single Cell 3′ v3.1 (Dual Index) protocol (CG000315 Rev A). Libraries were pooled, loaded onto a single 10B 100 Cycle Flowcell, and sequenced using an Illumina NovaSeq 6000 system to generate 25-30,000 paired-end 2 × 100 bp reads.

bioRxiv preprint doi: https://doi.org/10.1101/2025.02.20.639313; this version posted February 25, 2025. The copyright holder for this preprint (which was not certified by peer review) is the author/funder, who has granted bioRxiv a license to display the preprint in perpetuity. It is made available under aCC-BY 4.0 International license.

Data analysis: FastQ files were processed with the 10X Genomics Cell Ranger pipeline (v7.1.0) to demultiplex reads, align them to the mouse genome (mm10-2020-A), remove PCR duplicates, and generate gene expression matrices. Cell Ranger filtered outputs were analyzed using Seurat v4.3.0[26], and mitochondrial RNA content per cell was calculated using the GRCm39 (mm10) genome annotation and regressed out using SCTransform normalization protocol included in the Seurat toolkit with 20 principal components (PCs) and a resolution of 0.1. To estimate ambient RNA and correct for background contamination, the SoupX (v1.6.2) package[27] was used for each sample using raw and filtered feature matrices from the Cell Ranger output. Heterotypic doublets were identified and removed using DoubletFinder (v2)[28] to ensure the integrity of singlet datasets. Filtered singlet datasets were then re-normalized and integrated using the same Seurat SCTransform v2 workflow mentioned above. Cell clusters were annotated using a combination of expert curation based on published marker genes[29–35], and label transfer from hippocampal reference datasets, including the Allen Brain Map and Broad Institute resources[34,36]. Clusters with contaminant cell populations expressing markers for choroid plexus (*Ttr*), ependymal (*Tmem212*), and vascular leptomeningeal cells (*Vtn, Col1a2*) were removed from the analysis[35,37–39]. Additionally, as the sequential 2mm micropunches encompassed portions of cortical, thalamic and vHpc regions, clusters characterized by enrichment of published non-dHpc neuronal markers using Seurat's FindMarkers function (layer 5/6 cortical: *Rorb, Foxp2*[39]; ventral granule neurons: *Tox3*[31]) were also removed from the analysis. Cell cluster proportion analyses were conducted using the scProportionTest package[40], which employs a Monte Carlo permutation test to evaluate whether observed differences result from random sampling. Proportional differences between conditions were compared to a null distribution generated by resampling, and statistical significance was determined by permutation-based p-values with confidence intervals estimated via bootstrapping.

bioRxiv preprint doi: https://doi.org/10.1101/2025.02.20.639313; this version posted February 25, 2025. The copyright holder for this preprint (which was not certified by peer review) is the author/funder, who has granted bioRxiv a license to display the preprint in perpetuity. It is made available under aCC-BY 4.0 International license.

Differential expression analysis was conducted using pseudobulk analysis, where gene counts were summed across all cells within each sample for each cell type cluster using the AggregateExpression() function. DESeq2 was then applied at the sample level to conduct differential expression. To explore pathways underlying cluster-specific differences across conditions, pathway analysis was conducted using ShinyGO on genes meeting the following criteria: $log2FC > 1.5$ and $p < 0.05$.

DATA AND MATERIALS AVAILABILITY

The RNA-seq data generated in this study have been deposited in the National Center for Biotechnology Information Gene Expression Omnibus (GEO) database under accession numbers GSE289991 and GSE289992. We declare that the data supporting findings for this study are available within the article and Supplementary Information. Related data are available from the corresponding author upon reasonable request. No restrictions on data availability apply.

bioRxiv preprint doi: https://doi.org/10.1101/2025.02.20.639313; this version posted February 25, 2025. The copyright holder for this preprint (which was not certified by peer review) is the author/funder, who has granted bioRxiv a license to display the preprint in perpetuity. It is made available under aCC-BY 4.0 International license.

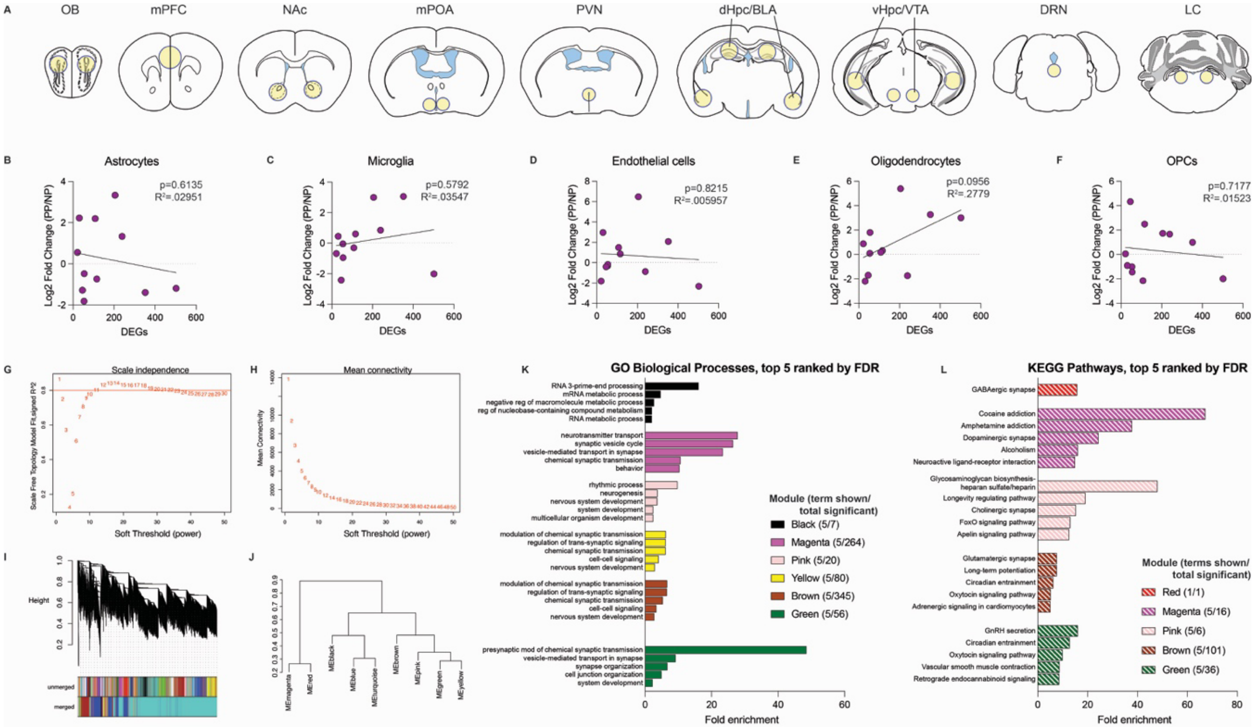

Figure S1: Brain-wide transcriptomic analysis of cell markers and gene expression networks. **A)** Brain regions selected for bulk transcriptional profiling: olfactory bulb (OB), medial prefrontal cortex (mPFC), nucleus accumbens (NAc), medial preoptic area (mPOA), paraventricular nucleus (PVN), dorsal hippocampus (dHpc), basolateral amygdala (BLA), ventral hippocampus (vHpc), ventral tegmental area (VTA), dorsal raphe nucleus (DRN), and locus coeruleus (LC). Yellow circles indicate area selected for tissue micropunching. **B-F)** Nonsignificant correlations between the fold change (NP *vs.* PP) in normalized surrogate proportion variables generated from cell-type deconvolution of bulk RNA-seq data for **B)** astrocytic, **C)** microglia, **D)** endothelial, **E)** mature oligodendrocyte, and **F)** oligodendrocyte precursor cell markers with the number of DEGs identified from each brain region. **G)** Scale independence plot for soft-threshold power selection for WGCNA analysis, illustrating the relationship between β and the scale-free topology fit index, identifying the optimal β value = 12. **H)** Mean connectivity plot illustrating the average network density for each evaluated soft-thresholding power. **I)** Dendrogram of gene clustering based on topological overlap, highlighting the hierarchical clustering process used for module detection and merging of similar modules. **J)** Dendrogram of eigengene network adjacency depicting similarity of modules. **K, L)** Significantly enriched **K)** GO Biological Processes and **L)** KEGG pathways, sorted by FDR values, from module gene sets. Bars are color-coded to indicate modules. The total number of significant terms for each analysis is provided.

bioRxiv preprint doi: https://doi.org/10.1101/2025.02.20.639313; this version posted February 25, 2025. The copyright holder for this preprint (which was not certified by peer review) is the author/funder, who has granted bioRxiv a license to display the preprint in perpetuity. It is made available under aCC-BY 4.0 International license.

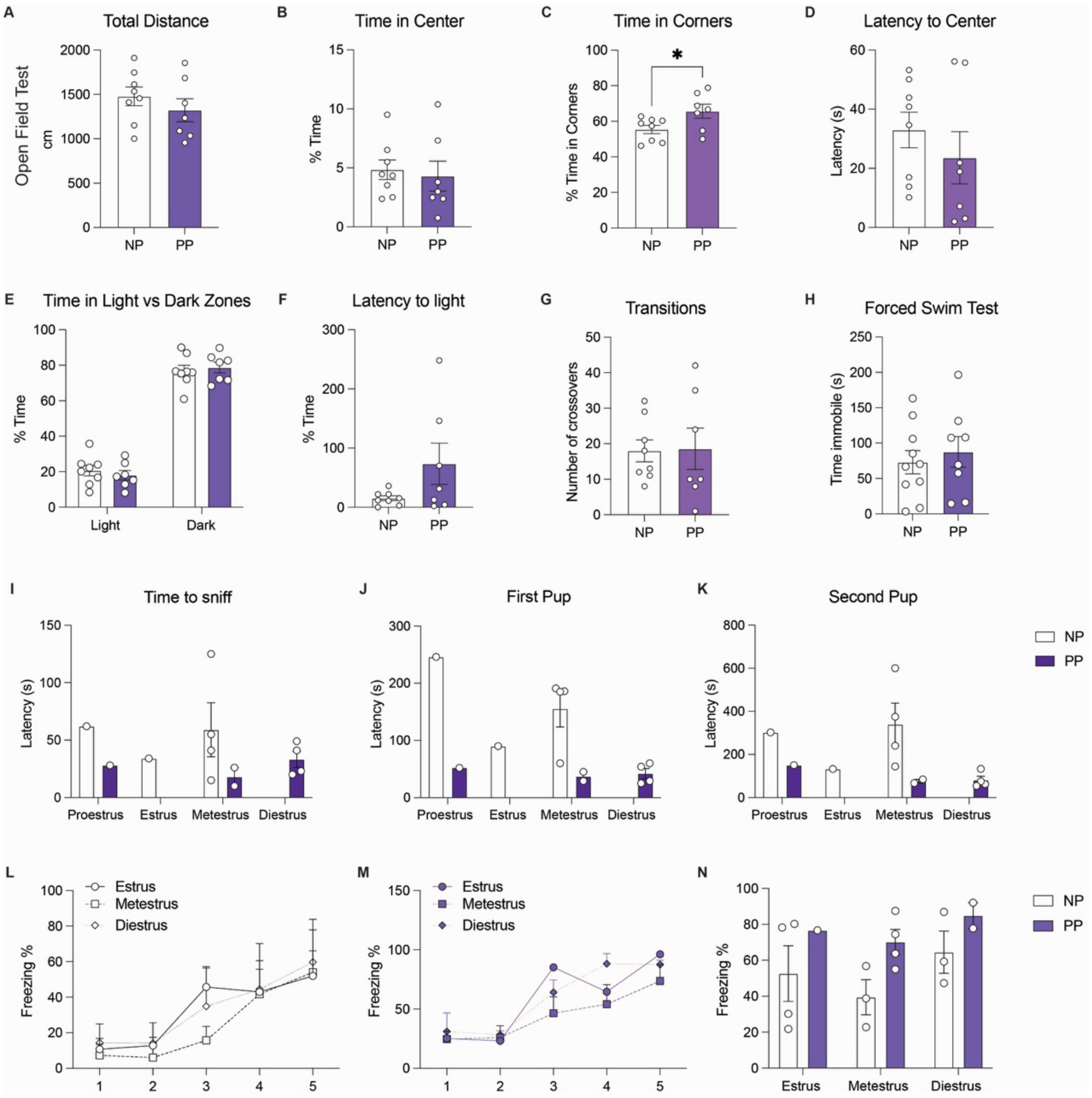

Figure S2: Parity associated behavioral adaptations are not influenced by locomotion, anxiety-/depressive-like behaviors, or estrous stage. A-D) In the open field test, there was no change in total distance, time spent in the center, or latency to the center. While there was a significant increase in time spent in corners for PP dams (Student's t-test, $t(13) = 2.307$, $*p = 0.0382$), this did not impact any other outcomes on the open field test. **E-G)** In the light-dark box, there was no difference in time spent in lights *vs.* dark zones, latency to enter the light zone, or transitions between zones. **H)** There was no difference in time spent immobile on the forced swim test. **I-K)** Stratification of pup retrieval behaviors by estrous stage on the day of testing did not reveal a significant effect of estrous stage. **L-N)** Similarly, stratification of contextual fear conditioning outcomes did not identify a significant effect of estrous stage on acquisition or context recall in NP or PP groups. Error bars represent mean ± SEM. N=6-11 animals/group.

bioRxiv preprint doi: https://doi.org/10.1101/2025.02.20.639313; this version posted February 25, 2025. The copyright holder for this preprint (which was not certified by peer review) is the author/funder, who has granted bioRxiv a license to display the preprint in perpetuity. It is made available under aCC-BY 4.0 International license.

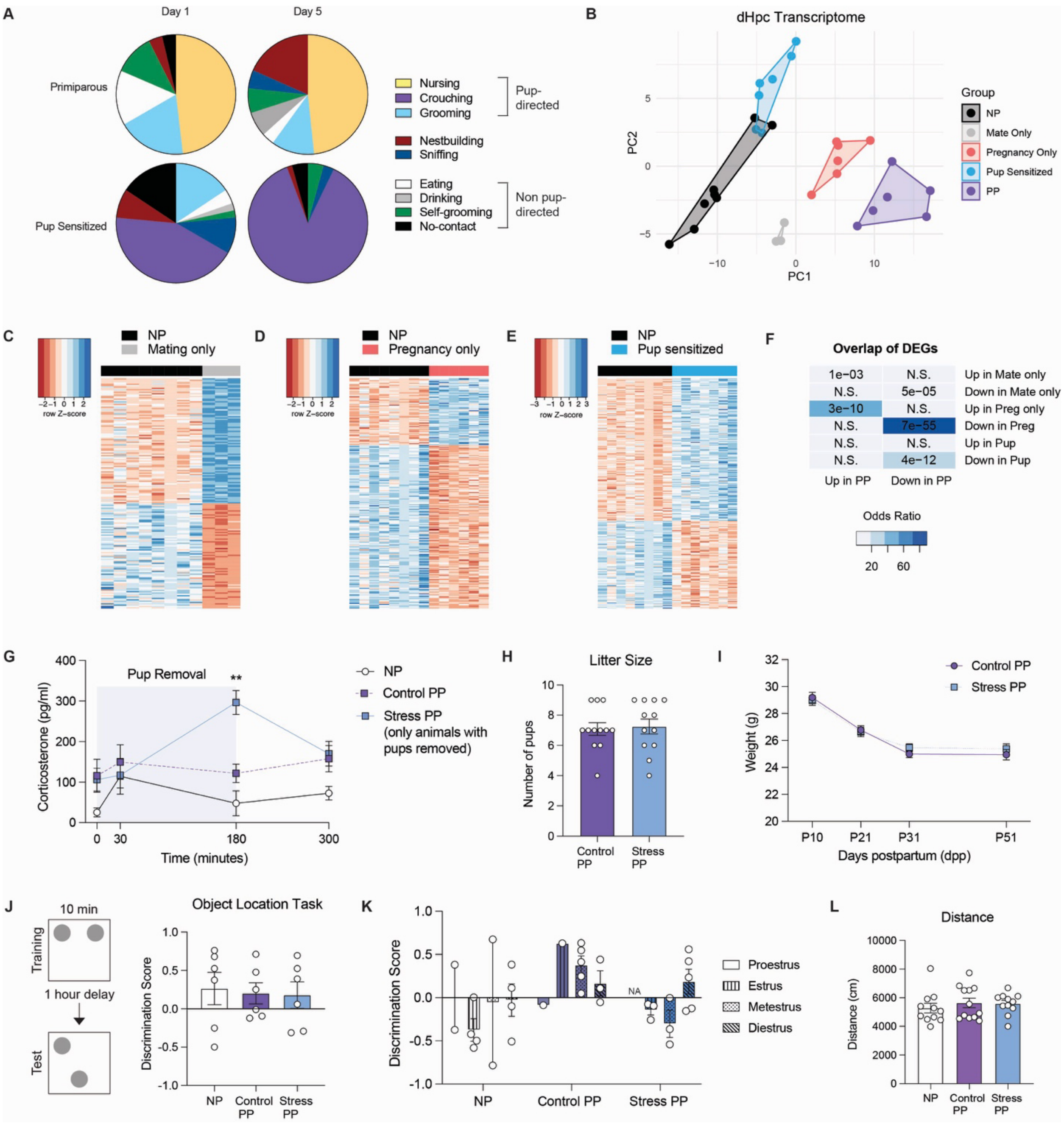

Figure S3: Postpartum experiences modulate the extent of parity-associated dHpc transcriptomic and behavioral adaptations. A) Pie charts showing significant shifts towards pup-directed behaviors, representing maternal behavior initiation after four days of pup exposure in Pup Sensitized virgin females ($\chi^2(7) = 33.52$, $p = 2.11 \times 10^{-5}$). **B)** Principle components analysis of NP *vs.* PP DEGs (adj $p < 0.05$) from dHpc, showing the Pregnancy Only group clusters most closely with PP. **C)** Heatmaps of all significant DEGs comparing NP *vs.* Mating only, **D)** NP *vs.* Pregnancy only, **E)** NP *vs.* Pup sensitized dHpc transcriptomes. **F)** Odds ratio analysis of DEG overlap for all comparisons (*vs.* NP). Insert numbers indicate respective *p* values for each association (N.S., $p > 0.05$). **G)** Pup separation increases maternal corticosterone levels over 3-

bioRxiv preprint doi: https://doi.org/10.1101/2025.02.20.639313; this version posted February 25, 2025. The copyright holder for this preprint (which was not certified by peer review) is the author/funder, who has granted bioRxiv a license to display the preprint in perpetuity. It is made available under aCC-BY 4.0 International license.

hours (RM two-way ANOVA, group: $F(2,15) = 12.86$, $p = 0.0006$, time: $F(2.125, 31.87) = 2.759$, $p = 0.0756$, interaction: $F(6,45) = 3.294$, $p = 0.009$; Tukey's multiple comparisons test: Control *vs.* Stress PP, **$p < 0.01$). N = 6 animals/group. **H)** No effect of postpartum stress on litter size. N = 12/group. **I)** Postpartum stress did not alter maternal weights. **J)** All groups discriminated the novel location following 10-minutes of training on the object location task (one way ANOVA, $F(2,15) = 0.06452$, $p = 0.9378$) N=6 animals/group. **K)** Stratification of object location test scores did not show a significant effect of estrous stage. **L)** There was no difference in the total distance travelled in the open field test across groups. Error bars represent mean ± SEM.

bioRxiv preprint doi: https://doi.org/10.1101/2025.02.20.639313; this version posted February 25, 2025. The copyright holder for this preprint (which was not certified by peer review) is the author/funder, who has granted bioRxiv a license to display the preprint in perpetuity. It is made available under aCC-BY 4.0 International license.

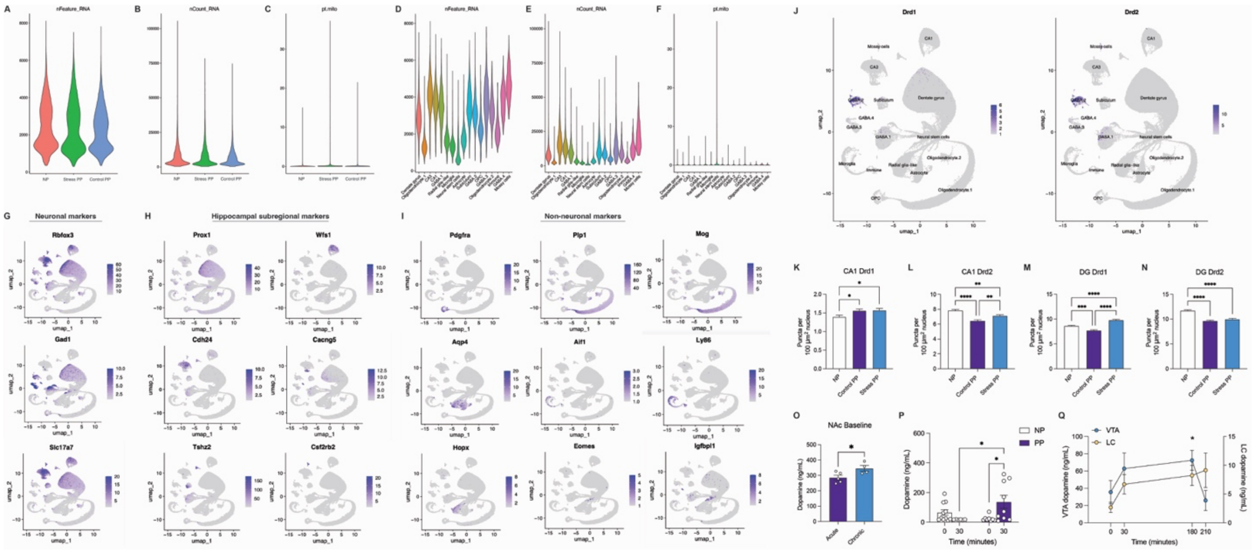

Figure S4: snRNA-seq quality control assessments, cell marker gene expression, and postpartum dopamine modulation. Violin plots showing the **(A)** number of detected genes, **(B)** UMI number, and **(C)** mitochondrial gene % by group following regression for mitochondrial gene mapping. **(D-F)** Breakdown of these metrics by annotated clusters show <10% mitochondrial gene across all cell-types, except for a minority of neural stem cells. Unsupervised clustering of snRNA-seq data with cells colored for **(G)** neuronal, excitatory, and inhibitory markers; **(H)** hippocampal subregional markers; and **(I)** non-neuronal markers including for glial and neural progenitor populations. **J)** Unsupervised clustering of snRNA-seq data with cells colored for *Drd1* (left) and *Drd2* (right) expression. **K)** Quantification of *Drd1* mRNA puncta in CA1 nuclei (one-way ANOVA, ($F(2,19204) = 3.963$, $p = 0.019$). **L)** Quantification of *Drd2* mRNA puncta in CA1 nuclei (one-way ANOVA, ($F(2,19204) = 23.16$, $p < 0.0001$). **M)** Quantification of *Drd1* mRNA puncta in dentate gyrus nuclei (one-way ANOVA, ($F(2,21864) = 42.09$, $p < 0.0001$). **N)** Quantification of *Drd2* mRNA puncta in dentate gyrus nuclei (one-way ANOVA, $(2,21864) = 41.79$, $p < 0.0001$). Tukey's multiple comparisons test, *$p < 0.05$, **$p < 0.01$, ***$p < 0.001$, ****$p < 0.0001$. **O)** Chronic postpartum stress resulted in higher baseline levels of dopamine in the nucleus accumbens (NAc; Student's t-test, $t(7) = 2.568$, *$p = 0.0371$). N= 4-5 animals/group. **P)** Dopamine response is not induced in NP dHpc 30-minutes following littermate separation (two-way ANOVA, interaction of time x group ($F(1,30) = 8.78$, $p = 0.0059$, time ($F(1,30) = 1.47$, $p = 0.2359$, group, ($F(1,30) = 2.054$, $p = 0.1621$); Tukey's multiple comparisons test, *$p < 0.05$). N = 6-11 animals/group. **Q)** Dopamine levels increased during pup separation in the VTA (left y-axis), but not in the locus coeruleus (right y-axis), resembling patterns of NAc and dHpc dopamine dynamics (two-way ANOVA, effect of brain region ($F(1,46) = 32.32$, $p < 0.0001$), time ($F(3,46) = 2.311$, $p = 0.0866$), interaction ($F(3,46) = 1.118$, $p = 0.1109$); Dunnett's multiple comparisons test, *$p < 0.05$). N = 5-8 samples/group. Error bars represent mean ± SEM.

bioRxiv preprint doi: https://doi.org/10.1101/2025.02.20.639313; this version posted February 25, 2025. The copyright holder for this preprint (which was not certified by peer review) is the author/funder, who has granted bioRxiv a license to display the preprint in perpetuity. It is made available under aCC-BY 4.0 International license.

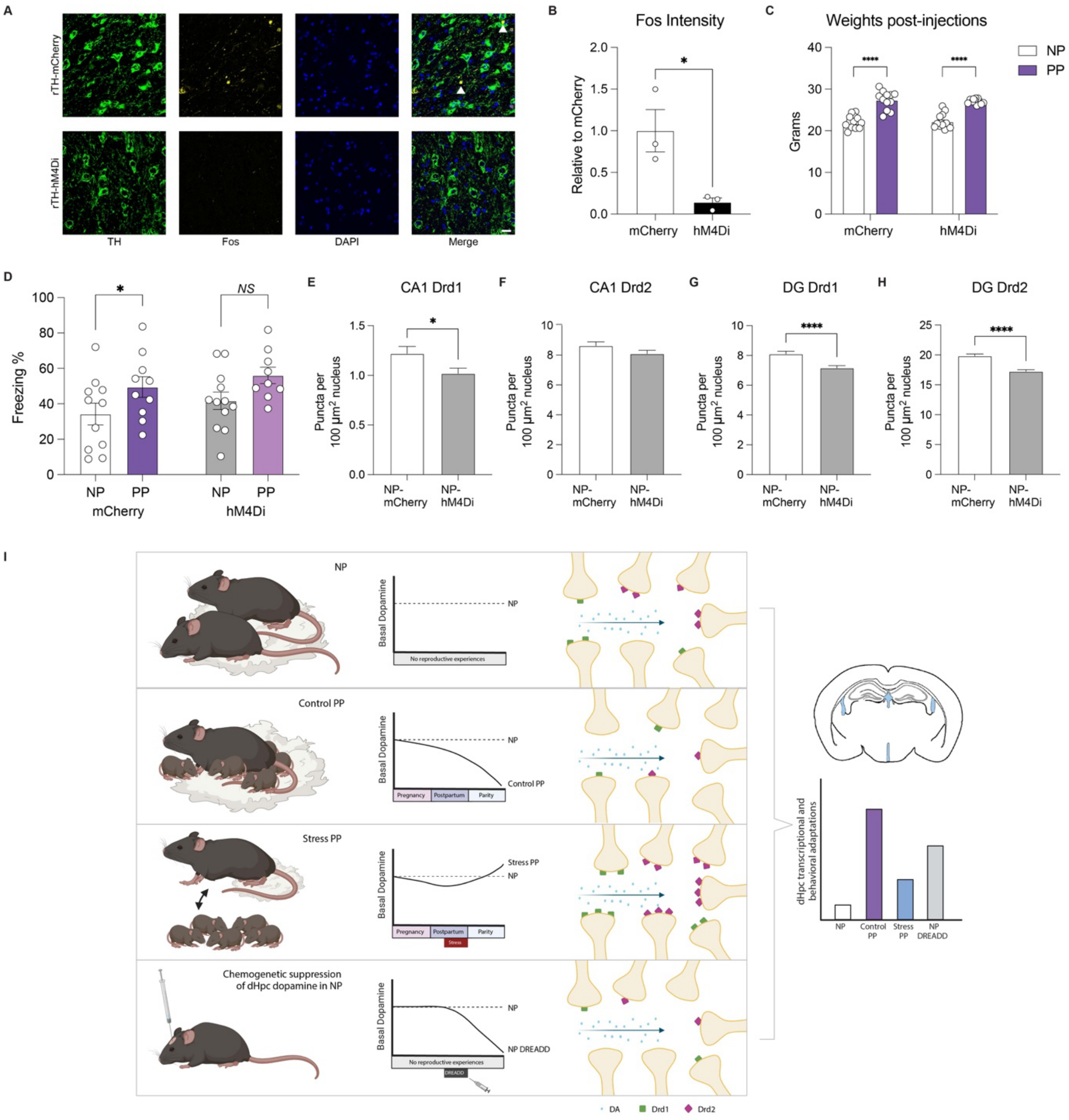

Figure S5. Inhibition of VTA-dHpc projection mimics sustained changes in dopamine receptor dynamics. A) Representative images of c-Fos immunostaining in the VTA. Scale bars, 20 µm. **B)** Quantification of c-Fos immunoreactivity in VTA TH+ neurons (Student's t-test; $t(4) = 3.299$, $*p = 0.03$). N = 3 animals/group. **C)** Chronic DCZ injections did not alter weights at 21 dpp (two-way ANOVA; main effect of group ($F(1,48) = 128$, $p < 0.0001$), virus ($F(1,48) = 0.0862$, $p = 0.7702$); Tukey's multiple comparison's test, $****p < 0.0001$). **D)** Parity induced significantly greater freezing on the context recall text in mCherry dams, with trending effects between hM4Di groups (two-way ANOVA (effect of group, ($F(1,38) = 7.177$, $p = 0.0109$, virus ($F(1,38) = 1.631$,

bioRxiv preprint doi: https://doi.org/10.1101/2025.02.20.639313; this version posted February 25, 2025. The copyright holder for this preprint (which was not certified by peer review) is the author/funder, who has granted bioRxiv a license to display the preprint in perpetuity. It is made available under aCC-BY 4.0 International license.

p = 0.2093, Fisher's LSD, *p £ 0.05). N = 9-12/group. **E)** Quantification of *Drd1* mRNA puncta in CA1 nuclei (Student's t-test, (t(4854) = 2.249, *p = 0.00246). **F)** Quantification of *Drd2* mRNA puncta in CA1 nuclei (Student's t-test, (t(4854) = 1.502, p = 0.1132). **G)** Quantification of *Drd1* mRNA puncta in dentate gyrus nuclei (Student's t-test, (t(11946) = 3.900, ****p < 0.0001). **H)** Quantification of *Drd2* mRNA puncta in dentate gyrus nuclei (Student's t-test, (t(11946) = 6.034, ****p < 0.0001). **I)** Schematic of working model.

bioRxiv preprint doi: https://doi.org/10.1101/2025.02.20.639313; this version posted February 25, 2025. The copyright holder for this preprint (which was not certified by peer review) is the author/funder, who has granted bioRxiv a license to display the preprint in perpetuity. It is made available under aCC-BY 4.0 International license.

SUPPLEMENTAL REFERENCES

76. Rice, C. J., Sandman, C. A., Lenjavi, M. R. & Baram, T. Z. A novel mouse model for acute and long-lasting consequences of early life stress. *Endocrinology* **149**, 4892–4900 (2008).
77. Gilles, E. E., Schultz, L. & Baram, T. Z. Abnormal corticosterone regulation in an immature rat model of continuous chronic stress. *Pediatr Neurol* **15**, 114–119 (1996).
78. Peña, C. J. *et al.* Early life stress confers lifelong stress susceptibility in mice via ventral tegmental area OTX2. *Science* **356**, 1185–1188 (2017).
79. Fleming, A. S. & Rosenblatt, J. S. Maternal behavior in the virgin and lactating rat. *Journal of Comparative and Physiological Psychology* **86**, 957–972 (1974).
80. Krishnan, V. *et al.* Molecular Adaptations Underlying Susceptibility and Resistance to Social Defeat in Brain Reward Regions. *Cell* **131**, 391–404 (2007).
81. Carcea, I. *et al.* Oxytocin neurons enable social transmission of maternal behaviour. *Nature* **596**, 553–557 (2021).
82. Wolcott, N. S. *et al.* Automated classification of estrous stage in rodents using deep learning. *Sci Rep* **12**, 17685 (2022).
83. Nagai, Y. *et al.* Deschloroclozapine, a potent and selective chemogenetic actuator enables rapid neuronal and behavioral modulations in mice and monkeys. *Nat Neurosci* **23**, 1157–1167 (2020).
84. Secci, M. E., Reed, T., Quinlan, V., Gilpin, N. W. & Avegno, E. M. Quantitative Analysis of Gene Expression in RNAscope-processed Brain Tissue. *Bio Protoc* **13**, e4580 (2023).
85. Bray, N. L., Pimentel, H., Melsted, P. & Pachter, L. Near-optimal probabilistic RNA-seq quantification. *Nature biotechnology* **34**, 525–527 (2016).
86. Risso, D., Ngai, J., Speed, T. P. & Dudoit, S. Normalization of RNA-seq data using factor analysis of control genes or samples. *Nat Biotechnol* **32**, 896–902 (2014).
87. Peixoto, L. *et al.* How data analysis affects power, reproducibility and biological insight of RNA-seq studies in complex datasets. *Nucleic Acids Res* **43**, 7664–7674 (2015).
88. Love, M. I., Huber, W. & Anders, S. Moderated estimation of fold change and dispersion for RNA-seq data with DESeq2. *Genome Biol* **15**, 550 (2014).
89. Ge, S. X., Jung, D. & Yao, R. ShinyGO: a graphical gene-set enrichment tool for animals and plants. *Bioinformatics* **36**, 2628–2629 (2020).
90. Supek, F., Bošnjak, M., Škunca, N. & Šmuc, T. REVIGO Summarizes and Visualizes Long Lists of Gene Ontology Terms. *PLOS ONE* **6**, e21800 (2011).
91. Krämer, A., Green, J., Pollard, J., Jr & Tugendreich, S. Causal analysis approaches in Ingenuity Pathway Analysis. *Bioinformatics* **30**, 523–530 (2014).
92. Shen, L. GeneOverlap: An R package to test and visualize gene overlaps.
93. Matevossian, A. & Akbarian, S. Neuronal Nuclei Isolation from Human Postmortem Brain Tissue. *J Vis Exp* 914 (2008) doi:10.3791/914.
94. Hao, Y. *et al.* Integrated analysis of multimodal single-cell data. *Cell* **184**, 3573-3587.e29 (2021).
95. Young, M. D. & Behjati, S. SoupX removes ambient RNA contamination from droplet-based single-cell RNA sequencing data. *Gigascience* **9**, giaa151 (2020).
96. McGinnis, C. S., Murrow, L. M. & Gartner, Z. J. DoubletFinder: Doublet Detection in Single-Cell RNA Sequencing Data Using Artificial Nearest Neighbors. *Cell systems* **8**, 329-337.e4 (2019).

bioRxiv preprint doi: https://doi.org/10.1101/2025.02.20.639313; this version posted February 25, 2025. The copyright holder for this preprint (which was not certified by peer review) is the author/funder, who has granted bioRxiv a license to display the preprint in perpetuity. It is made available under aCC-BY 4.0 International license.

97. Artegiani, B. *et al.* A Single-Cell RNA Sequencing Study Reveals Cellular and Molecular Dynamics of the Hippocampal Neurogenic Niche. *Cell Reports* **21**, 3271–3284 (2017).
98. Zhou, Z., Zhi, C., Chen, D., Cai, Z. & Jiang, X. Single-nucleus RNA sequencing reveals cell type-specific transcriptome alterations of Down syndrome hippocampus using the Dp16 mouse model. *Genes Genomics* **45**, 1305–1315 (2023).
99. Cembrowski, M. S., Wang, L., Sugino, K., Shields, B. C. & Spruston, N. Hipposeq: a comprehensive RNA-seq database of gene expression in hippocampal principal neurons. *eLife* **5**, e14997.
100. Harris, K. D. *et al.* Classes and continua of hippocampal CA1 inhibitory neurons revealed by single-cell transcriptomics. *PLoS Biol* **16**, e2006387–e2006387 (2018).
101. Haslinger, A., Schwarz, T. J., Covic, M. & Lie, D. C. Expression of Sox11 in adult neurogenic niches suggests a stage-specific role in adult neurogenesis. *Eur J Neurosci* **29**, 2103–2114 (2009).
102. Bandler, R. C. *et al.* Single-cell delineation of lineage and genetic identity in the mouse brain. *Nature* **601**, 404–409 (2022).
103. Olney, K. C. *et al.* Widespread choroid plexus contamination in sampling and profiling of brain tissue. *Mol Psychiatry* **27**, 1839–1847 (2022).
104. Xiang, M. *et al.* A Single-Cell Transcriptional Roadmap of the Mouse and Human Lymph Node Lymphatic Vasculature. *Front Cardiovasc Med* **7**, 52 (2020).
105. Tasic, B. *et al.* Adult Mouse Cortical Cell Taxonomy by Single Cell Transcriptomics. *Nat Neurosci* **19**, 335–346 (2016).
106. Miller, S. A. *et al.* LSD1 and aberrant DNA methylation mediate persistence of enteroendocrine progenitors that support BRAF mutant colorectal cancer. *Cancer Res* **81**, 3791–3805 (2021).
